# Vildagliptin and its metabolite M20.7 induce the expression of S100A8 and S100A9 in human hepatoma HepG2 and leukemia HL-60 cells

**DOI:** 10.1038/srep35633

**Published:** 2016-10-19

**Authors:** Mitsutoshi Asakura, Fumika Karaki, Hideaki Fujii, Koichiro Atsuda, Tomoo Itoh, Ryoichi Fujiwara

**Affiliations:** 1 Graduate School of Pharmaceutical Sciences (M.A.) and School of Pharmacy, Kitasato University, Tokyo, Japan

## Abstract

Vildagliptin is a potent, orally active inhibitor of dipeptidyl peptidase-4 (DPP-4) for the treatment of type 2 diabetes mellitus. It has been reported that vildagliptin can cause hepatic dysfunction in patients. However, the molecular-mechanism of vildagliptin-induced liver dysfunction has not been elucidated. In this study, we employed an expression microarray to determine hepatic genes that were highly regulated by vildagliptin in mice. We found that pro-inflammatory S100 calcium-binding protein (S100) a8 and S100a9 were induced more than 5-fold by vildagliptin in the mouse liver. We further examined the effects of vildagliptin and its major metabolite M20.7 on the mRNA expression levels of S100A8 and S100A9 in human hepatoma HepG2 and leukemia HL-60 cells. In HepG2 cells, vildagliptin, M20.7, and sitagliptin – another DPP-4 inhibitor – induced S100A9 mRNA. In HL-60 cells, in contrast, S100A8 and S100A9 mRNAs were significantly induced by vildagliptin and M20.7, but not by sitagliptin. The release of S100A8/A9 complex in the cell culturing medium was observed in the HL-60 cells treated with vildagliptin and M20.7. Therefore, the parental vildagliptin- and M20.7-induced release of S100A8/A9 complex from immune cells, such as neutrophils, might be a contributing factor of vildagliptin-associated liver dysfunction in humans.

Vildagliptin (LAF237) is a potent, orally active inhibitor of dipeptidyl peptidase-4 (DPP-4; EC 3.4.14.5, also known as CD26) for the treatment of type 2 diabetes mellitus^1^. DPP-4 inhibitors, so-called incretin enhancers, are attracting attention among therapeutic agents for type 2 diabetes mellitus, since they improve glucose control with a low risk of hypoglycemia^2^,^3^. To date, at least eleven DPP-4 inhibitors have been approved in the world^4^. While most DPP-4 inhibitors allow single oral administration per day for management of type 2 diabetes mellitus, twice-daily administration is recommended for vildagliptin because of its shorter half-life^3^. Major metabolic pathway of vildagliptin is hydrolysis at the cyano group to produce a carboxylic acid metabolite M20.7 (LAY151), which is pharmacologically inactive^5^. It has been reported that the parent compound and the major metabolite M20.7 account for the majority of vildagliptin-related materials in human plasma (approximately 25.7 and 55%, respectively) and the liver is the major site of vildagliptin metabolism in humans^5^,^6^. Human nitrilase-like proteins and cytochrome P450s did not exhibit the formation of M20.7^5^,^7^. Although the major metabolic enzyme responsible for vildagliptin hydrolysis in humans was unknown, we previously demonstrated that DPP-4, which is the target of the DPP-4 inhibitors, greatly contributed to the hydrolysis of vildagliptin in human livers^8^.

Drug-induced liver injury is a rare but serious adverse reaction and the most frequent reason for withdrawal from the market. Recently, it has been suggested that activation of the innate immune systems by drugs or their reactive metabolites is involved in the pathogenesis of the immune-mediated drug-induced liver injury as one of the factors^9^,^10^. A number of immune- and inflammation-related factors, such as S100 calcium-binding protein (S100), cytokines, and chemokines, have been implicated in the pathogenesis of drug-induced liver injury^10^,^11^,^12^,^13^. In several studies using human monocytic cell lines, such as THP-1 and HL-60 cells, and *in vivo* mouse models, it has been suggested that the induction of the inflammation-associated genes, including S100A8, S100A9, tumor necrosis factor-α (TNF-α), and interleukin-8, by drug and/or its metabolites is involved in drug-induced liver injury^11^,^12^,^14^,^15^,^16^,^17^.

S100A8 and S100A9 are members of the calcium-binding S100-protein family and are released at inflammatory sites by phagocytes as a complex (S100A8/A9; also called calprotectin or MRP8/14)^18^. Constitutive expression of S100A8 and S100A9 is largely restricted to phagocytic myeloid cells, in particular neutrophils and monocytes. S100A8/A9 complex, which is a ligand for Toll-like receptors, induces a variety of inflammatory reactions and the extent of S100A8/A9 expression correlates with disease activity in several inflammatory disorders^19^,^20^. Additionally, a previous report has shown that, based on findings from the *in vivo* data in the lipopolysaccharide (LPS)-treated wild-type and S100A9-deficient mice, S100A9 or S100A8/A9 complex was involved in the LPS-induced liver inflammation and injury^21^. Therefore, S100A8 and S100A9 are recently attracting attention as key factors in promoting inflammation and markers for inflammation. It has been reported that vildagliptin caused hepatic dysfunction in patients^3^,^22^,^23^. Although the molecular-mechanism of vildagliptin-induced liver injury remains to be elucidated, it was previously suggested that immune responses might play a predominant role in the vildagliptin-induced liver dysfunction^23^. Therefore, we hypothesized that immune-associated genes, such as S100A8 and S100A9, were induced by vildagliptin, causing the hepatotoxicity.

In the present study, we investigated the molecular-mechanism of vildagliptin-induced liver injury. First, we employed an expression microarray analysis to determine hepatic genes that were highly regulated by vildagliptin in mice. Second, we examined the effects of vildagliptin and M20.7 on mRNA expression levels of inflammation-associated genes, such as S100A8, S100A9, and TNF-α, in human hepatoma HepG2 and monocytic HL-60 cells. Finally, we examined the effects of vildagliptin and M20.7 on the release of S100A8/A9 complex from the human cell lines.

## Results

### Effect of vildagliptin on gene expression in mouse liver

**Figure 1:**
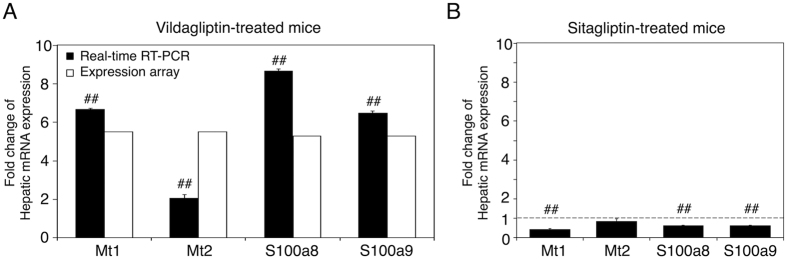
Effect of vildagliptin and sitagliptin on hepatic gene expression in mice. The expression levels of 4 selected genes (metallothionein (Mt) 1, Mt2, S100a8, and S100a9) in the liver of vildagliptin (1000 mg/kg)-treated mice were measured by real-time RT-PCR analysis, and vildagliptin-induced fold changes were compared with those obtained from expression array analysis (**A**). The expression levels of Mt1, Mt2, S100a8, and S100a9 in the liver of sitagliptin (1000 mg/kg)-treated mice were also measured by real-time RT-PCR analysis (**B**). In the real-time RT-PCR analysis, expression was normalized with the expression of cyclophilin (CPH) and the expression level in the liver of control mice was defined as 1. Data represent the means ± S.D. of three independent experiments. ^##^P < 0.01, compared with the liver of control mice.

We performed an expression microarray to determine hepatic genes that were highly regulated by vildagliptin in mice. Total RNA was isolated from the pooled livers of two control or vildagliptin-treated mice and was subjected to the microarray analysis, which contains 55,681 biological probes. The numbers of detected probes in the RNA from the liver of control and vildagliptin-treated mice were 28,006 and 29,146, respectively. Among probes that were detected in both the samples (the livers of control and vildagliptin-treated mice), the number of probes increased more than 2-fold was 276. Among probes that were detected in the liver of vildagliptin-treated mice and not detected in the liver of control mice, the number of probes increased more than 5-fold was 466. Table 1 shows the top 14 and 3 genes among the 276 and 466 genes that were up-regulated more than 2-fold and 5-fold, respectively. Among probes that were detected in both the samples (the livers of control and vildagliptin-treated mice), the number of probes decreased more than 2-fold was 292. Among probes that were detected in the liver of control mice and not detected in the liver of vildagliptin-treated mice, the number of probes decreased more than 5-fold was 478. The top 10 genes among the 292 and 478 genes that were down-regulated more than 2-fold and 5-fold are shown as Supplemental Table S1. To provide more information of our expression array, we added raw data of expression array as a Supplementary material. In this microarray analysis, we found that metallothionein (Mt) 1, Mt2, S100a8, and S100a9 were induced more than 5-fold by vildagliptin in mouse liver. The expressions of MT1, MT2, S100A8, and S100A9 were reported to be associated with drug-induced liver injury^11^,^12^,^24^,^25^. To evaluate the changes in gene expression determined by expression array analysis, we performed real-time reverse transcription-polymerase chain reaction (RT-PCR) for Mt1, Mt2, S100a8, and S100a9 mRNA using hepatic total RNA of control and vildagliptin (1000 mg/kg)-treated mice. The fold changes of mRNA expression of Mt1, Mt2, S100a8, and S100a9 in the liver of vildagliptin-treated mice were significantly higher than that in the liver of control mice (Fig. 1A). These results indicate that fold changes in gene expression determined by expression array analysis are reproducible by real-time RT-PCR and that the hepatic genes, including Mt1, Mt2, S100a8, and S100a9, were highly regulated by vildagliptin in mice. Furthermore, we performed a real-time RT-PCR for Mt1, Mt2, S100a8, and S100a9 mRNA using hepatic total RNA of control and sitagliptin (1000 mg/kg)-treated mice. Sitagliptin is another DPP-4 inhibitor. The mRNA expression levels of hepatic Mt1, Mt2, S100a8, and S100a9 were not induced by the sitagliptin treatment (Fig. 1B), suggesting that inhibition of DPP-4 was not the cause of the induction of Mt1, Mt2, S100a8, and S100a9 mRNA by vildagliptin.

### Effect of vildagliptin on gene expression in HepG2 cells

**Figure 2:**
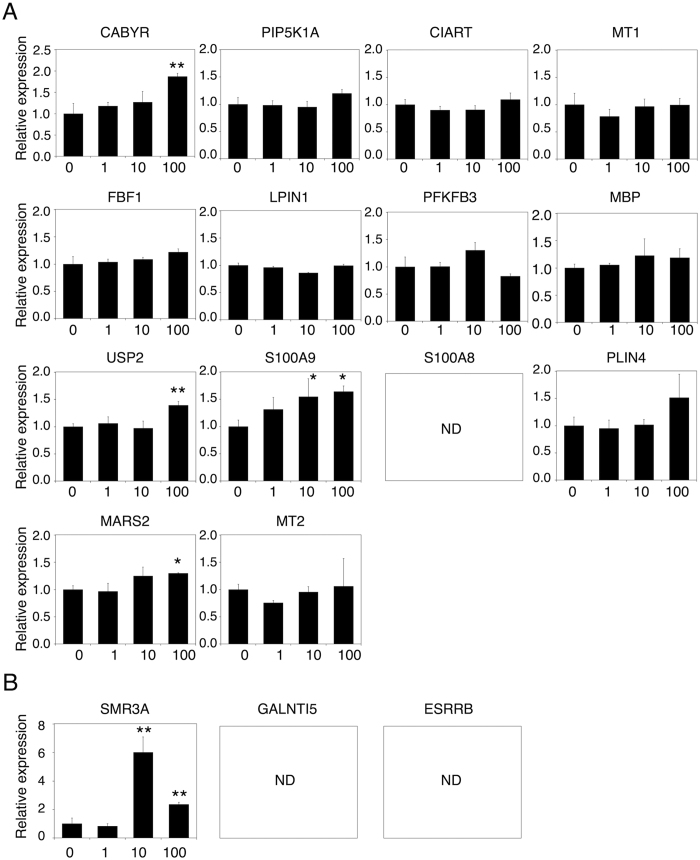
Effect of vildagliptin on gene expression in HepG2 cells. HepG2 cells were treated with 0 (water), 1, 10, or 100 μM of vildagliptin for 24 h. The mRNA expression levels of the 17 selected genes, which were increased more than 2-fold (**A**) or 5-fold (**B**) by vildagliptin in mouse liver, were measured in HepG2 cells by real-time RT-PCR analysis. Expression was normalized with the expression of glyceraldehyde-3-phosphate dehydrogenase (GAPDH). Data represent the means ± S.D. of three independent experiments. *P < 0.05; **P < 0.01, compared with vildagliptin 0 μM (water). ND, not detectable.

To investigate whether vildagliptin induces the mRNA expression levels of the 17 selected genes (Table 1) in human liver, HepG2 cells were treated with vildagliptin for 24 h and then mRNA expression levels were measured by real-time RT-PCR. Because the therapeutic maximum plasma concentration (C_max_) of vildagliptin after the therapeutic dose of 100 mg/day is approximately 1 μM^26^,^27^, HepG2 cells were treated with 1 μM vildagliptin (Fig. 2). Additionally, HepG2 cells were treated with 10 μM and 100 μM vildagliptin, which is 10-fold and 100-fold higher than the C_max_, respectively. Among the 17 genes in Table 1, the mRNA expression levels of calcium-binding tyrosine phosphorylation regulated protein (CABYR), ubiquitin specific peptidase 2 (USP2), S100A9, methionine-tRNA synthetase 2 (MARS2), and submaxillary gland androgen regulated protein 3A (SMR3A) were statistically higher in vildagliptin-treated HepG2 cells than those in control HepG2 cells (Fig. 2). In contrast, the mRNA expression levels of the 9 genes, including MT1 and MT2, were not induced by vildagliptin in HepG2 cells. The mRNA expression levels of S100A8, polypeptide N-acetylgalactosaminyltransferase-like 5 (GALNTL5), and estrogen-related receptor beta (ESRRB) in HepG2 cells could not be quantified by real-time RT-PCR. These results suggest that vildagliptin can induce the mRNA expression of CABYR, USP2, S100A9, MARS2, and SMR3A in human hepatocytes.

### Dose-dependent changes in the mRNA expression levels of S100A9 and TNF-α in HepG2 cells treated with vildagliptin, M20.7, and sitagliptin

**Figure 3:**
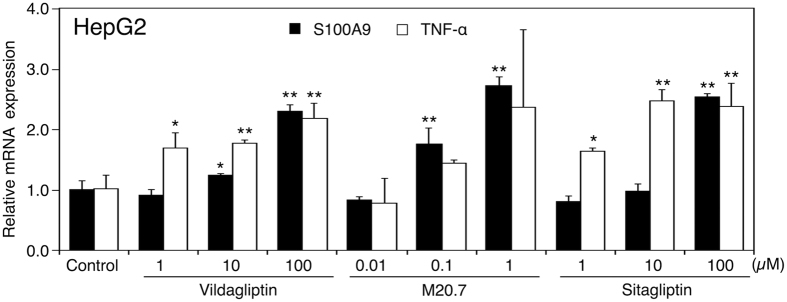
Effects of vildagliptin, M20.7, and sitagliptin on gene expression in HepG2 cells. HepG2 cells were treated with the indicated concentration (μM) of vildagliptin, M20.7, or sitagliptin for 24 h. The mRNA expression levels of S100A9 and TNF-α in HepG2 cells were measured by real-time RT-PCR analysis. Expression was normalized with the expression of GAPDH. Data represent the means ± S.D. of three independent experiments. *P < 0.05; **P < 0.01, compared with control (0.1% methanol).

S100A9 can be associated with drug-induced liver injury^11^,^12^. TNF-α was reported to induce the mRNA expression levels of S100A8 and S100A9^28^,^29^. Therefore, we further investigated the effects of vildagliptin, its major metabolite M20.7, and sitagliptin, on the mRNA expression levels of S100A9 and TNF-α in HepG2 cells. The C_max_ of M20.7 after the therapeutic dose of vildagliptin (100 mg/day) is approximately 1 μM^26^. The C_max_ of sitagliptin after the therapeutic dose of 100 mg/day is also approximately 1 μM^30^. In the HepG2 cells, vildagliptin increased the mRNA expression levels of S100A9 and TNF-α in a concentration-dependent manner (Fig. 3). M20.7 and sitagliptin also increased the mRNA expression levels of S100A9 and TNF-α in HepG2 in a concentration-dependent manner. Additionally, vildagliptin, M20.7, and sitagliptin increased the mRNA expression levels of S100A9 and TNF-α in HepG2 at concentration of 1 μM. These results suggest that vildagliptin, M20.7, and sitagliptin can increase the mRNA expression levels of S100A9 and TNF-α in human hepatocytes.

### Dose-dependent changes in the mRNA expression levels of S100A8/A9 and TNF-α in HL-60 cells treated with vildagliptin, M20.7, and sitagliptin

**Figure 4:**
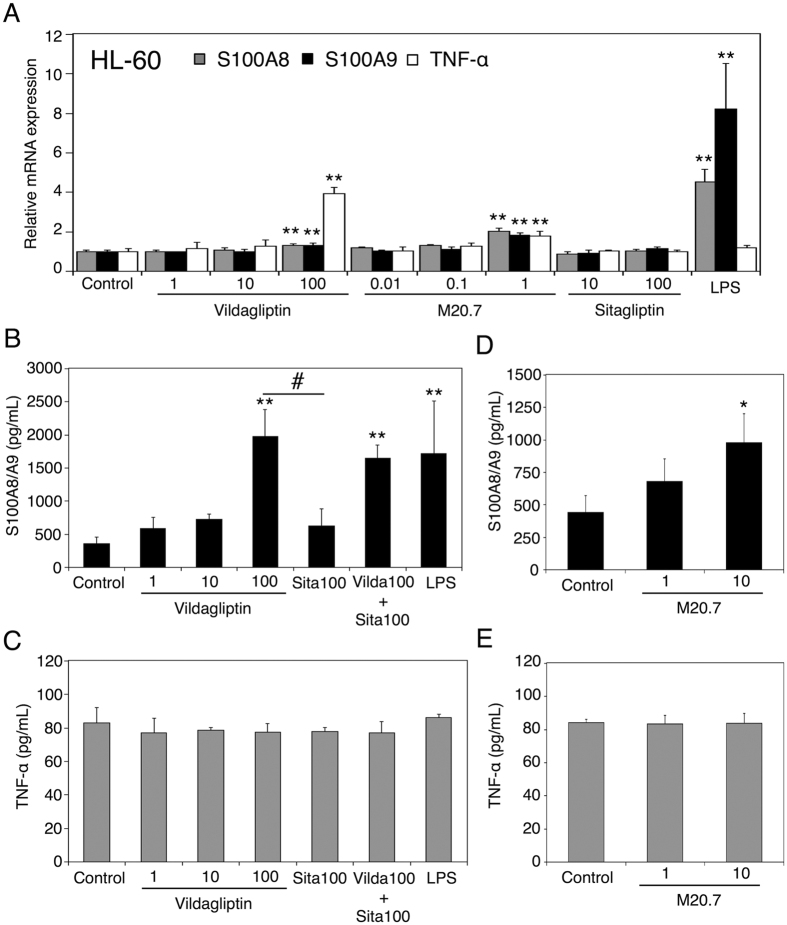
Effects of vildagliptin and M20.7 on gene expression in HL-60 cells. (**A**) HL-60 cells were treated with the indicated concentration (μM) of vildagliptin, M20.7, sitagliptin, or LPS for 24 h. The mRNA expression levels of S100A9 and TNF-α in HL-60 cells were measured by real-time RT-PCR analysis. Expression was normalized with the expression of GAPDH. (**B–E**) HL-60 cells were treated with the indicated concentration (μM) of vildagliptin, M20.7, sitagliptin, or LPS for 48 h. The release of S100A8/A9 complex (**B**,**D**) and TNF-α (**C**,**E**) in the culturing medium was measured by ELISA. LPS (10 μg/mL) was used as a positive control. Data represent the means ± S.D. of three independent experiments. *P < 0.05; **P < 0.01, compared with control (0.1% methanol). ^#^P < 0.05, compared with 100 μM sitagliptin. Sita 100, sitagliptin 100 μM; Vilda 100 + Sita 100, vildagliptin 100 μM + sitagliptin 100 μM.

While HL-60 cells, human monocytic leukemia cell lines, can be differentiated to mature neutrophil-like cells following a treatment with 1.3% DMSO for 3–4 days^31^,^32^,^33^, Yano *et al*.^17^ recently reported that untreated HL-60 cells responded well to hepatotoxic drugs treatment in terms of the induction of the aforementioned immune and inflammatory genes, including S100A8 and S100A9, indicating that untreated HL-60 cells are useful *in vitro* models for studying the immune-mediated drug-induced liver injury, particularly in the induction of S100A8 and S100A9 by drugs or their reactive metabolites. Therefore, we used untreated HL-60 cells to examine the effects of vildagliptin, M20.7, and sitagliptin on the mRNA expression levels of S100A8, S100A9, and TNF-α. HL-60 cells were treated with vildagliptin, M20.7, or sitagliptin for 24 h and then mRNA expression levels were measured by real-time RT-PCR. S100A8 and S100A9 were highly expressed in neutrophils and monocytes and were induced by LPS in those cells^28^,^29^,^34^. In the present study, LPS increased the mRNA expression levels of S100A8 and S100A9 in HL-60 cells (Fig. 4A), which is in agreement with the previous report^17^. Vildagliptin and M20.7 increased the mRNA expression levels of S100A8, S100A9, and TNF-α in HL-60 cells at concentration of 100 μM and 1 μM, respectively. However, the mRNA expression levels of S100A8, S100A9, and TNF-α in HL-60 cells were not changed by the treatment with sitagliptin at 10 μM and 100 μM, which is 10-fold and 100-fold higher than the C_max_, respectively. These findings indicate that not only vildagliptin, but also its metabolite M20.7 is involved in the induction of mRNA expression levels of S100A8, S100A9, and TNF-α in HL-60 cells. The data also suggest that the structure of vildagliptin and M20.7 rather than inhibition effect of DPP-4 activity is associated with the gene induction.

### Effects of vildagliptin and M20.7 on the release of S100A8/A9 complex and TNF-α from HL-60 cells

To investigate whether vildagliptin increases the releases of S100A8/A9 complex and TNF-α from human immune cells, HL-60 cells were treated with vildagliptin for 48 h and then the release of S100A8/A9 complex and TNF-α in the cell culturing medium was measured by ELISA. The concentrations of S100A8/A9 complex in the cell culturing medium of control-, 1 μM vildagliptin-, 10 μM vildagliptin-, and 100 μM vildagliptin-treated HL-60 cells were 355 ± 102, 588 ± 171, 731 ± 72, and 1982 ± 403 pg/mL, respectively (Fig. 4B), and those of 100 μM sitagliptin-, 100 μM vildagliptin with 100 μM sitagliptin-, and LPS-treated HL-60 cells were 624 ± 266, 1654 ± 191, and 1723 ± 790 pg/mL, respectively. The release of S100A8/A9 complex from HL-60 cells was significantly increased by the treatment with 100 μM vildagliptin or LPS but not by 100 μM sitagliptin compared with control. The concentration of S100A8/A9 complex in the cell culturing medium of 100 μM vildagliptin-treated HL-60 cells was more than 3-fold higher than that of 100 μM sitagliptin-treated HL-60 cells. On the other hand, the release of TNF-α from HL-60 cells was not changed by the treatment with vildagliptin, sitagliptin, and LPS compared with control (Fig. 4C).

We further investigated the effect of M20.7 on the releases of S100A8/A9 complex and TNF-α from HL-60 cells. The concentrations of S100A8/A9 complex in the cell culturing medium of control-, 1 μM M20.7-, and 10 μM M20.7-treated HL-60 cells were 444 ± 127, 681 ± 172, and 981 ± 220 pg/mL, respectively (Fig. 4D). M20.7 increased the release of S100A8/A9 complex from HL-60 cells in a concentration-dependent manner, whereas the release of TNF-α from HL-60 cells was not changed by the treatment with M20.7 compared with control (Fig. 4E). These results indicate that vildagliptin and M20.7 could induce the release of S100A8/A9 complex from HL-60 cells via TNF-α–independent mechanisms.

### DPP-4 activity in HL-60 cells and time-dependent changes of M20.7 concentration in the culturing medium of vildagliptin-treated HL-60 cells

**Figure 5:**
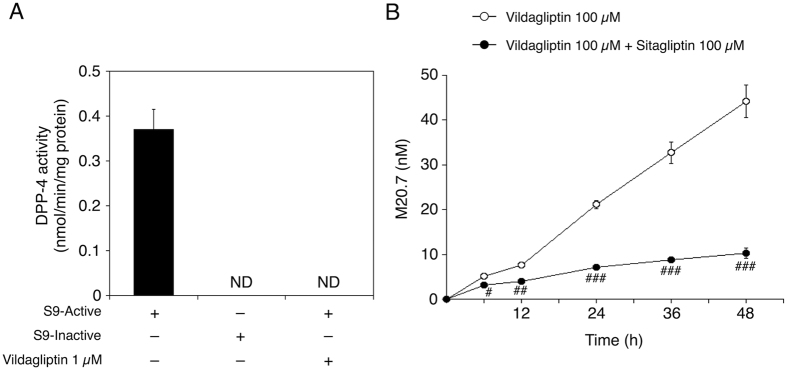
DPP-4 activity in HL-60 cells (**A**) and time-dependent changes of M20.7 concentration in the culturing medium of vildagliptin-treated HL-60 cells (**B**). (**A**) DPP-4 activity of S9 fraction of HL-60 cells was measured using a Gly-Pro-AMC as a substrate. Data represent the means ± S.D. of triplicate determinations. S9-active, active S9 fraction; S9-inactive, heat-inactivated S9 fraction (negative control); ND, not detectable. (**B**) HL-60 cells were treated with 100 μM vildagliptin with or without 100 μM sitagliptin for various durations. The M20.7 concentration in the culturing medium was measured by LC-MS/MS analysis. Data represent the means ± S.D. of three independent experiments. ^#^P < 0.05; ^##^P < 0.01; ^###^P < 0.001, compared with vildagliptin and sitagliptin-treated HL-60 cells of each time point.

M20.7 was involved in the induction of S100A8/A9 complex release from HL-60 cells (Fig. 4D), suggesting that M20.7, which was formed via the DPP-4-mediated hydrolysis, might be involved in the induction of S100A8 and S100A9 expression in vildagliptin-treated HL-60 cells. We next investigated whether the HL-60 cells had the vildagliptin-hydrolyzing activity. To investigate the protein expression of DPP-4 in HL-60 cells, we performed DPP-4 activity assay using S9 fraction of HL-60 cells. H-glycyl-prolyl-7-amino-4-methylcoumarin (Gly-Pro-AMC), which is a synthetic DPP-4-specific substrate, was used as a substrate. DPP-4 activity of S9 fraction of HL-60 cells was 0.37 ± 0.04 nmol/min/mg protein (Fig. 5A). DPP-4 activity of heat-inactivated S9 fraction of HL-60 cells was not detectable. Furthermore, DPP-4 activity of S9 fraction of HL-60 cells was completely inhibited by vildagliptin 1 μM. These data indicate that DPP-4 is functionally expressed in HL-60 cells.

To investigate whether the M20.7 is formed via the DPP-4-mediated hydrolysis in HL-60 cells, the M20.7 concentration in the culturing medium of 100 μM vildagliptin with or without 100 μM sitagliptin-treated HL-60 cells was quantified. Sitagliptin was used as a selective DPP-4 inhibitor. The M20.7 concentrations in the culturing medium of 100 μM vildagliptin-treated HL-60 cells at 6, 12, 24, 36, and 48 h were 5.0 ± 0.5, 7.6 ± 0.1, 21 ± 1.0, 33 ± 2.4, and 44 ± 3.6 nM, respectively (Fig. 5B), and that in the culturing medium of 100 μM vildagliptin with 100 μM sitagliptin-treated HL-60 cells at 6, 12, 24, 36, and 48 h were 3.1 ± 0.1, 3.9 ± 0.5, 7.1 ± 0.3, 8.7 ± 0.2, and 10 ± 1.2 nM, respectively. The M20.7 concentration in the culturing medium was increased in a time-dependent manner. Furthermore, the M20.7 concentration in HL-60 cells treated with vildagliptin and sitagliptin was significantly decreased compared with that in HL-60 cells treated with vildagliptin. These results indicate that M20.7 is formed via the DPP-4-mediated hydrolysis in the culturing medium of vildagliptin-treated HL-60 cells.

## Discussion

While drugs provide numerous benefits to the body, they can also exhibit various adverse reactions. In fact, not only mild adverse reactions, but also severe toxicity such as hepatic dysfunctions were observed in patients treated with vildagliptin. At the time of initial registration of vildagliptin in EU, elevations in alanine aminotransferase (ALT) and aspartate aminotransferase (AST) values were observed more in subjects who received 100 mg once-daily dose of vildagliptin than in placebo or 50 mg twice-daily-treated subjects^3^. For this reason, the recommended therapeutic dose of vildagliptin was fixed to 50 mg twice-daily. Additionally, it has been reported that a patient with diabetic nephropathy developed hepatotoxicity by 50 mg/day vildagliptin^23^. Vildagliptin and its metabolite M20.7 are ultimately excreted by the kidney^27^. While the actual serum concentrations of vildagliptin and M20.7 were not determined in this patient, there was the possibility that the blood concentrations of vildagliptin and M20.7 in the patient might have been significantly elevated. These observations indicated that individuals with a higher blood vildagliptin concentration might also develop vildagliptin-induced liver damage. There is only a study reporting the animal safety of vildagliptin, in which a long-term treatment of mice with vildagliptin (1000 mg/kg) did not increase the risk of developing liver injury in mice^35^. This indicates that experimental mice are relatively safe to vildagliptin, while there are still many cases of vildagliptin-induced liver injury in humans. To identify hepatic genes that were highly regulated by vildagliptin in mice, therefore, we employed the treatment method (1000 mg/kg for 24 h) in the present study. In the subsequent study, we employed a clinical concentration of vildagliptin in the treatment of human hepatic and monocytic cell lines.

HepG2 cells have been used as an *in vitro* human hepatocyte model for a wide variety of studies, including hepatotoxicity^36^,^37^,^38^. While various drug-metabolizing enzymes such as cytochrome P450s are highly expressed in the in liver, most of these enzymes are not expressed in cultured hepatoma HepG2 cells^39^, which has limited the detection of the metabolism-dependent toxicity of drugs^38^. However, cytochrome P450s did not catalyze the hydrolysis of vildagliptin^5^. Additionally, DPP-4, which is the major metabolic enzyme of vildagliptin^8^, is highly expressed in HepG2 cells^40^. On the other hand, recently, it has been suggested that activation of the innate immune systems by drugs or their reactive metabolites is involved in the pathogenesis of the immune-mediated drug-induced liver injury^9^,^10^. It has been reported that HL-60 cells can be differentiated to mature neutrophil-like cells following a treatment with 1.3% DMSO for 3–4 days^31^,^32^,^33^. In the present study, neutrophil-like HL-60 cells were treated with vildagliptin, M20.7, or sitagliptin for 24 h to investigate the effects of vildagliptin and M20.7 on the S100A8/A9 expression. The mRNA expression levels of S100A8 and S100A9 were induced more than 40-fold and 60-fold by the treatment with 1.3% DMSO for 3 days (Supplementary Fig. S1), confirming that HL-60 cells were differentiated to neutrophil-like HL-60 cells^33^. Vildagliptin increased the release of S100A8/A9 complex from neutrophil-like HL-60 cells at concentration of 10–100 μM (Supplementary Fig. S2). Although S100A8 and S100A9 were highly expressed in neutrophils and monocytes and were induced by LPS in those cells^28^,^29^,^34^, LPS did not increase the release of S100A8/A9 complex from neutrophil-like HL-60 cells (Supplementary Fig. S2). Furthermore, the release of S100A8/A9 complex from neutrophil-like HL-60 cells was induced by not only vildagliptin, but also sitagliptin, which was not consistent with the results of our *in vivo* study using vildagliptin (1000 mg/kg)-treated mice and sitagliptin (1000 mg/kg)-treated mice (Fig. 1). Although the neutrophil-like HL-60 cell is a good model to study the apoptotic processes involved in neutrophil programmed cell death, our results suggest that the neutrophil-like HL-60 cells are not appropriate to study the mechanisms of vildagliptin-specific immune-mediated liver injury. On the other hand, Yano *et al*.^17^ reported that untreated HL-60 cells responded well to hepatotoxic drug treatment in terms of the induction of the aforementioned immune and inflammatory genes, including S100A8 and S100A9. The main source of S100A8 and S100A9 is phagocytic myeloid cells, such as neutrophils and monocytes^18^. These findings indicate that untreated HL-60 cell line is a useful *in vitro* model for studying the immune-mediated drug-induced liver injury, particularly in the induction of S100A8 and S100A9 by drugs or their reactive metabolites. Therefore, we used HepG2 and untreated HL-60 cells to investigate the effects of vildagliptin and M20.7 on the induction of S100A8/A9.

The mRNA expression level of S100A9 was induced by vildagliptin and its major metabolite M20.7 in both HepG2 and HL-60 cells (Figs 3 and 4A). In contrast, the induction of release of S100A8/A9 complex by the treatment with vildagliptin and M20.7 was only observed in HL-60 cells (Fig. 4B,D). Our data indicated that *in vivo* hepatocytes might induce S100A9 mRNA, but not the release of S100A8/A9 complex. Our data also indicated that liver neutrophils and monocytes might induce both S100A8/A9 mRNA and the release of S100A8/A9 complex. We previously demonstrated that the contribution rate of DPP-4 to vildagliptin hydrolysis in human liver was approximately 60%^8^, whereas it was reported to be 20% in rats^41^. In the present study, the DPP-4 protein of HL-60 cells could catalyze the vildagliptin hydrolysis reaction (Fig. 5). Therefore, we believe that both HepG2 and HL-60 cells could produce the M20.7. To fully understand the molecular mechanism of vildagliptin-induced liver injury, species difference needs also to be considered.

It has been reported that TNF-α can promote the release of S100A8/A9 complex from neutrophils and monocytes^28^,^29^. However, our results indicated that vildagliptin and M20.7 induced the release of S100A8/A9 complex from immune cells TNF-α–independently (Fig. 4). It has been also reported that monosodium urate monohydrate crystals induced the release of S100A8/A9 complex from human neutrophils by activating CD11b, CD16, Src kinases, Syk, and tubulin polymerization^42^. Therefore, the activation of these factors by vildagliptin and M20.7 might be involved in the release of S100A8/A9 complex from vildagliptin- or M20.7-treated HL-60 cells.

DPP-4 inhibitors, including vildagliptin and sitagliptin, are oral anti-hyperglycemic agents for the treatment of type 2 diabetes. DPP-4 inhibitors that have been developed for therapeutic use are all competitive reversible inhibitors, which display high affinity for DPP-4, resulting in inhibition constants (K_i_) in the low nanomolar range^3^. The K_i_ values for inhibition of human DPP-4 by vildagliptin and sitagliptin are 13 nM and 18 nM, respectively^43^. There are differences in the way in which they interact with the DPP-4. For example, sitagliptin forms non-covalent interactions with residues in the catalytic site^3^,^44^. In contrast, it has been reported that vildagliptin forms a reversible covalent bounds with DPP-4 to inhibit DPP-4 enzymatic activity^44^,^45^. The covalent binding between vildagliptin and DPP-4 is a consequence of the formation of an unstable complex by reaction of the cyano group of vildagliptin with amino acid residue serine 630 within the catalytic domain of DPP-4^45^,^46^. It has been suggested that the amino acid residue serine 630 of DPP-4 is involved in formation of M20.7^5^,^45^. In the previous study, we demonstrated that the catalytic serine 630 residue is directly involved in the vildagliptin hydrolysis in human DPP-4 using S630A-expressing HEK293 cells^8^. Because both vildagliptin and sitagliptin interact with residues in the catalytic site of DPP-4, sitagliptin was used as not only a control of the inhibitory effect against DPP-4 activity but also a competitive inhibitor of the DPP-4-mediated hydrolysis of vildagliptin.

As shown Fig. 1, sitagliptin did not induce S100a8 or S100a9 in the liver, indicating that the induction of these genes by vildagliptin was not a result from inhibition of DPP-4. To support this, 1 μM vildagliptin did not induce S100A8 and S100A9 mRNA or their release in HL-60 cells (Fig. 4). Therefore, the relatively higher concentration of vildagliptin (100 μM) was required to investigate the mechanism of vildagliptin-induced liver injury. Previous studies have demonstrated the anti-inflammatory effect of vildagliptin, both *in vivo* and *in vitro* at the therapeutic blood concentration, which is approximately 1 μM^47^,^48^. In contrast, our data indicated that high blood or hepatic vildagliptin concentration (10–100 μM) might be associated with the development of liver inflammation. This inconsistency might be due to the DPP-4–independently reaction of vildagliptin. While vildagliptin has a selectivity for DPP-4, it has been reported that vildagliptin inhibits human DPP-8 and DPP-9 with K_i_ values of 5 μM and 0.3 μM, respectively^43^. Recently, it was shown that the vildagliptin-mediated inhibition of DPP-8 and DPP-9 was associated with induction of the leukemia stem cell death^49^. Therefore, the observed inflammatory effect by vildagliptin at high concentrations (Fig. 4) might have been a result from DPP-8 and DPP-9 inhibitions.

**Figure 6:**
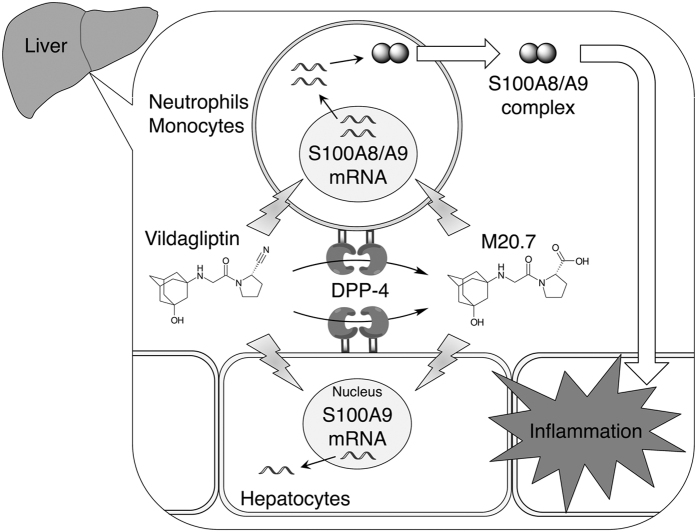
A proposed mechanism of vildagliptin-associated liver dysfunction. Vildagliptin is metabolized via DPP-4-mediated hydrolysis. Parental vildagliptin and its metabolite M20.7 induce the mRNA expressions of S100A8 and S100A9 and the release of S100A8/A9 complex from immune cells, such as neutrophils and monocytes, which result in inflammation in the liver.

M20.7 is a hydrolyzed metabolite of vildagliptin. Because M20.7 was involved in the induction of S100A8/A9 complex release from HL-60 cells in a concentration-dependent manner (Fig. 4D), M20.7 might have been involved in the induction of S100A8 and S100A9 expression in mice. Indeed, the DPP-4 activity was observed in HL-60 cells (Fig. 5A) and M20.7 was detected in the culturing medium of vildagliptin-treated HL-60 cells (Fig. 5B). Although we found that the formation of M20.7 in the culturing medium of HL-60 cells was significantly inhibited by sitagliptin (Fig. 5B), the release of S100A8/A9 complex from HL-60 cells treated with 100 μM vildagliptin and 100 μM sitagliptin was comparable with that from HL-60 cells treated with 100 μM vildagliptin alone (Fig. 4B). These results suggest that both parental vildagliptin and its major metabolite M20.7 are involved in the induction of S100A8/A9 complex release from neutrophils and monocytes (Fig. 6).

While vildagliptin is primarily metabolized via DPP-4-mediated hydrolysis, vildagliptin and M20.7 are ultimately excreted by the kidney^5^,^8^. Therefore, renal impairment, which is very common in patients with type 2 diabetes, can somewhat alter the pharmacokinetics of vildagliptin. In subjects with mild, moderate, and severe renal impairment, and end-stage renal disease patients on hemodialysis, systemic exposure to vildagliptin was increased (C_max_ 108–166%; area under the concentration-time curve (AUC) 132–234%) compared to subjects with normal renal function^2^. AUC of M20.7 was 1.6-, 2.4-, 5.4-, and 6.7-fold higher in subjects with mild, moderate, and severe renal impairment, and in those with end-stage renal disease, respectively, compared with healthy subjects^27^. These data indicate that the exposure to M20.7 rather than parental vildagliptin is increased according to renal impairment. Therefore, to avoid the onset of vildagliptin-associated liver injury, not only vildagliptin but also M20.7 needs to be carefully monitored in patients.

In conclusion, we found that S100a8 and S100a9 were induced more than 5-fold by vildagliptin in mouse liver, although higher dosage of vildagliptin was used. The induction of the mRNA expression levels of S100A8 and S100A9 by the treatment with vildagliptin and its major metabolite M20.7 was observed in human hepatic and monocytic cell lines. In the HL-60 cells, vildagliptin and M20.7 further induced the release of S100A8/A9 complex. The vildagliptin- and M20.7-induced release of S100A8/A9 complex from immune cells, such as neutrophils and monocytes, might be a contributing factor of vildagliptin-associated liver dysfunction.

## Materials and Methods

### Chemicals and reagents

Vildagliptin was synthesized in our laboratory using the standard technique^7^. Vildagliptin carboxylic acid metabolite (M20.7) was purchased from Santa Cruz Biotechnology (Delaware Avenue, CA, USA). Sitagliptin was obtained from LKT Laboratories (St. Paul, MN, USA). 7-Amino-4-methylcoumarin (AMC) was purchased from Setareh Biotech, LLC (Eugene, OR, USA). Gly-Pro-AMC was obtained from Bachem (Bubendorf, Switzerland). LPS was purchased from Sigma-Aldrich (St. Louis, MO, USA). Primers were commercially synthesized at Life Technologies (Carlsbad, CA, USA). All other chemicals were of the highest grade available.

### Animals and preparation of mouse livers

Male C57BL/6NCrSlc mice aged 8 weeks were purchased from Japan SLC (Shizuoka, Japan). All animals received food and water ad libitum, and mouse handling and experimental procedures were conducted in accordance with our animal care protocol approved by Kitasato University. Mice were orally treated with vehicle (water), vildagliptin (1000 mg/kg), and sitagliptin (1000 mg/kg), respectively. Twenty-four hours after the administration, the mice were anesthetized by diethyl ether inhalation, and the livers were perfused with ice-cold 1.15% KCl. The livers were rinsed in cold 1.15% KCl and stored at −80 °C until used for RNA extraction.

### Expression array

Total RNA was extracted from the pooled livers of two control or vildagliptin-treated mice using TRIzol reagent (Life Technologies). Complementary RNA (cRNA) was prepared from the total RNA using the Quick Amp Labeling Kit (Agilent Technologies, Santa Clara, CA, USA) following procedures recommended by the manufacturer. Briefly, 100 ng of the total RNA was reverse transcribed to complementary DNA (cDNA) followed by synthesis of cRNA incorporated with cyamine 3 (Cy3)-labeled nucleotide. cRNA was then purified using RNeasy mini columns (Qiagen, Hilden, Germany). Fluorescently labeled targets were hybridized to a SurePrint G3 Mouse GE 8 × 60 K DNA microarray containing 55,681 biological probes (Agilent Technologies). Hybridization and wash processes were performed according to the manufacturer’s instructions, and hybridized microarrays were scanned using an Agilent Microarray Scanner (Agilent Technologies). Feature Extraction software (ver 10.7.1.1) was employed for image analysis and data extraction processes.

### Cell culture

The human hepatoma HepG2 and promyelocytic leukemia HL-60 cells were obtained from DS Pharma Biomedical Co., Ltd. (Osaka, Japan). HepG2 cells were grown in Dulbecco’s modified Eagle’s medium containing 100 U/mL penicillin, 100 μg/mL streptomycin, and 10% fetal bovine serum. HL-60 cells were grown in RPMI 1640 medium containing 100 U/mL penicillin, 100 μg/mL streptomycin, and 20% fetal bovine serum. These cells were maintained at 37 °C in a humidified atmosphere containing 5% CO_2_.

### Drug treatment of HepG2 and HL-60 cells

HepG2 and HL-60 cells were seeded at a density of 1 × 10^5^ and 1 × 10^6^ cells/well, respectively, in 12-well plates with the culture medium containing the indicated concentration (μM) of vildagliptin, M20.7, sitagliptin, or LPS (10 μg/mL) and then incubated at 37 °C for 24 or 48 h. The final concentration of methanol in the culture medium was 0.1%. Total RNA from HepG2 or HL-60 cells was extracted using TRIzol reagent according to the manufacturer’s instructions. The supernatants of culture medium were separated from the cells by centrifugation and stored at −80 °C until assayed.

### Real-time RT-PCR

cDNA was synthesized from total RNA using ReverTra Ace qPCR RT Master Mix (Toyobo, Tokyo, Japan) according to the manufacturer’s protocol. Real-time RT-PCR was performed with THUNDERBIRD SYBR qPCR Mix (Toyobo), and the reactions were run in a CFX96 Real-Time PCR Detection System (Bio-Rad, Hercules, CA, USA). After an initial denaturation at 95 °C for 30 s, amplification was performed by denaturation at 95 °C for 5 s and annealing and extension at 60 °C for 30 s for 45 cycles. The primer sequences are summarized in Supplemental Table S2. Expression was normalized with the expression of cyclophilin (CPH) or glyceraldehyde-3-phosphate dehydrogenase (GAPDH).

### Enzyme-linked immunosorbent assay (ELISA)

The pro-inflammatory S100A8/A9 complex and cytokine TNF-α in the cell culturing medium were measured by Legend MAX Human MRP8/14 ELISA kit (BioLegend, San Diego, CA, USA) and Human TNF-α ELISA Max Deluxe sets (BioLegend), respectively, according to the manufacturer’s instructions.

### DPP-4 activity assays

HL-60 cells were suspended in cold phosphate buffered saline and homogenized with a teflon-glass homogenizer for 40 strokes. The total cell homogenate was centrifuged at 600 g for 10 min at 4 °C. The pellet, which contained nuclear fraction, was discarded. The supernatant, which contained mostly cell protein, was further centrifuged at 9,000 g for 20 min at 4 °C and the supernatant was used as the S9 fraction.

DPP-4 activity was determined by cleavage rate of AMC from the synthetic substrate Gly-Pro-AMC, as described previously^8^, with some modifications. Briefly, the diluted S9 fraction (100 μg/mL) of HL-60 cells was incubated for 15 min at room temperature in assay buffer (50 mM glycine, pH 8.7, 1 mM EDTA). Heat-inactivated (treated at 95 °C for 5 min) S9 fraction was used as a negative control. Fifteen min after the incubation, Gly-Pro-AMC was added at the final concentration of Gly-Pro-AMC was 50 μM and the plates were incubated for 10 min at 25 °C. Fluorescence was measured using a SpectraMax M5 96-well plate spectrophotometer (excitation, 360 nm; emission, 460 nm, Molecular Devices, Sunnyvale, CA, USA). The DPP-4 activity was expressed as the amount of cleaved AMC per minute per mg protein (nmol/min/mg protein).

### Quantification of M20.7 in the culturing medium of vildagliptin-treated HL-60 cells

HL-60 cells were seeded at a density of 1 × 10^6^ cells/well in 12-well plates with the culture medium containing 100 μM vildagliptin with or without 100 μM sitagliptin and then incubated at 37 °C for 6, 12, 24, 36, or 48 h. The supernatants of culture medium were separated from the cells by centrifugation and then 100-μL of acetonitrile was added to a 100-μL portion of the culture medium. After removal of protein by centrifuged at 15,000 g for 5 min, a 10-μL portion of the sample was subjected to liquid chromatography/tandem mass spectrometry (LC-MS/MS) analysis to measure the concentration of M20.7.

### LC-MS/MS conditions

A Waters ACQUITY UPLC system (Waters, Milford, MA, USA) was connected to a Waters Xevo TQD mass spectrometer (Waters) operated in the positive electrospray ionization mode. M20.7 was separated on a Polaris 5 μm C18-A 50 × 2.0-mm column (25 °C) (Agilent Technologies, Amstelveen, The Netherlands) with a MetaGuard 2.0 mm Polaris 5 μm C18-A guard column (Agilent Technologies). The mobile phase of A/B (1:3, v/v) was used, where A was methanol/10 mM ammonium acetate, pH 8.0 (5:95, v/v), and B was acetonitrile/methanol (10:90, v/v). The flow rate was adjusted to 0.2 mL/min, and an eluent between 0 and 5 min was introduced into the mass spectrometer. The sample was analyzed during the multiple reaction monitoring mode of the mass spectrometer at a dwell time of 0.1 s per channel using m/z 323.1 > 173.3 as the transition. The ionization conditions were as follows: capillary voltage, 3.4 kV; cone voltage, 42 V; collision energy, 18 V; source temperature, 150 °C; desolvation temperature, 200 °C; collision gas, argon. Data acquisition, instrument control and data handling were performed with MassLynx Software (version 4.1; Waters).

### Statistical analysis

Data were presented as means ± S.D. and were assessed for statistical significance using the unpaired t-test or Dunnett’s test. A value of *P* < 0.05 was considered statistically significant.

## Additional Information

**How to cite this article**: Asakura, M. *et al*. Vildagliptin and its metabolite M20.7 induce the expression of S100A8 and S100A9 in human hepatoma HepG2 and leukemia HL-60 cells. *Sci. Rep.* **6**, 35633; doi: 10.1038/srep35633 (2016).

## Supplementary Material

Supplementary Information

Supplementary Information

## Figures and Tables

**Table 1:** Top 14 and 3 probes that were up-regulated more than 2-fold and 5-fold among the 276 and 466 genes.

	GeneSymbol	Gene name	Fold change (Vildagliptin/Control)
Up-regulated more than 2-fold*	Cabyr	Calcium-binding tyrosine phosphorylation regulated protein	**13.82**
	Pip5k1a	Phosphatidylinositol-4-phosphate 5-kinase, type 1 alpha	**6.56**
	Ciart	Circadian associated repressor of transcription	**6.55**
	Mt1	Metallothionein 1	**6.45**
	Fbf1	Fas (TNFRSF6) binding factor 1	**5.99**
	Lpin1	Lipin 1	**5.97**
	Pfkfb3	6-phosphofructo-2-kinase/fructose-2,6-biphosphatase 3	**5.76**
	Mbp	Myelin basic protein	**5.76**
	Usp2	Ubiquitin specific peptidase 2	**5.56**
	S100a9	S100 calcium binding protein A9 (calgranulin B)	**5.52**
	S100a8	S100 calcium binding protein A8 (calgranulin A)	**5.52**
	Plin4	Perilipin 4	**5.46**
	Mars2	Methionine-tRNA synthetase 2 (mitochondrial)	**5.30**
	Mt2	Metallothionein 2	**5.29**
Up-regulated more than 5-fold**	Smr3a	Submaxillary gland androgen regulated protein 3A	**102.79**
	Galntl5	Polypeptide N-acetylgalactosaminyltransferase-like 5	**66.62**
	Esrrb	Estrogen-related receptor beta	**66.20**

^*^Among probes that were detected in both the samples (the livers of control and vildagliptin-treated mice), the probes increased more than 2-fold were shown.

^**^Among probes that were detected in the liver of vildagliptin-treated mice and not detected in the liver of control mice, the probes increased more than 5-fold were shown.
