# Supplementary material for: Vildagliptin and its metabolite M20.7 induce the expression of S100A8 and S100A9 in human hepatoma HepG2 and leukemia HL-60 cells
Source: Sci Rep. 2016 Oct 19;6:35633. doi: 10.1038/srep35633 (PMC5069476; doi:10.1038/srep35633)
Supplement: Supplementary Information [file srep35633-s1.pdf]

Vildagliptin and its metabolite M20.7 induce the expression of S100A8 and S100A9 in human hepatoma HepG2 and leukemia HL-60 cells

Mitsutoshi Asakura, Fumika Karaki, Hideaki Fujii, Koichiro Atsuda, Tomoo Itoh & Ryoichi Fujiwara

Supplementary materials

Microarray data_Up276

Narabi_55681	ProbeName	LC(normalized)	LV(normalized)	Fold change	GeneSymbol	GeneName
6403	A_30_P01023827	0.089	1.476	**16.521**		
54219	A_66_P110769	0.362	5.000	**13.822**	Cabyr	calcium-binding tyrosine-(Y)-phosphorylation regulated (fibrousheathin 2)
44546	A_55_P2107070	0.553	5.978	**10.805**	Cabyr	calcium-binding tyrosine-(Y)-phosphorylation regulated (fibrousheathin 2)
54693	A_66_P120230	0.122	0.896	**7.343**	LOC102637016	Y-linked testis-specific protein 1-like
18862	A_51_P256549	0.134	0.928	**6.907**	Gm3527	predicted gene 3527
33963	A_55_P1999902	24.668	161.857	**6.561**	Pip5k1a	phosphatidylinositol-4-phosphate 5-kinase, type 1 alpha
27659	A_52_P63343	0.341	2.234	**6.553**	Ciart	circadian associated repressor of transcription
25953	A_52_P423810	3.027	19.533	**6.452**	Mt1	metallothionein 1
7542	A_30_P01024966	0.081	0.498	**6.165**		
36963	A_55_P2027879	0.122	0.728	**5.986**	Fbf1	Fas (TNFRSF6) binding factor 1
32434	A_55_P1986019	0.086	0.513	**5.974**		
42445	A_55_P2083474	0.099	0.590	**5.967**	Lpin1	lipin 1
45336	A_55_P2116165	2.591	14.912	**5.756**	Pfkfb3	6-phosphofructo-2-kinase/fructose-2,6-biphosphatase 3
25219	A_52_P329451	0.113	0.651	**5.755**	Mbp	myelin basic protein
32406	A_55_P1985788	3.254	18.106	**5.564**	Usp2	ubiquitin specific peptidase 2
50829	A_55_P2186395	0.133	0.736	**5.542**	LOC102640775	cyclin-dependent kinases regulatory subunit 1-like
33785	A_55_P1998471	1.076	5.942	**5.524**	S100a9	S100 calcium binding protein A9 (calgranulin B)
18866	A_51_P256827	0.309	1.708	**5.521**	S100a8	S100 calcium binding protein A8 (calgranulin A)
22845	A_51_P495780	0.795	4.343	**5.460**	Plin4	perilipin 4
12703	A_30_P01030127	0.086	0.457	**5.331**		
21175	A_51_P396385	0.091	0.484	**5.296**	Mars2	methionine-tRNA synthetase 2 (mitochondrial)
30128	A_55_P1965861	0.205	1.085	**5.291**	Zfp939	zinc finger protein 939
18702	A_51_P246317	124.381	658.034	**5.290**	Mt2	metallothionein 2
54273	A_66_P111660	170.017	897.855	**5.281**	Mt1	metallothionein 1
36519	A_55_P2023617	0.095	0.497	**5.221**	Kiss1r	KISS1 receptor

28211	A_52_P79506	0.140	0.713	**5.093**	Zfp667	zinc finger protein 667
38113	A_55_P2039061	0.234	1.179	**5.039**	Trim12c	tripartite motif-containing 12C
12723	A_30_P01030147	0.144	0.703	**4.894**		
34280	A_55_P2002773	0.123	0.601	**4.890**	1810053B23Rik	RIKEN cDNA 1810053B23 gene
6720	A_30_P01024144	0.154	0.733	**4.758**		
46418	A_55_P2129614	0.768	3.641	**4.740**	Itprip	inositol 1,4,5-triphosphate receptor interacting protein
42446	A_55_P2083481	5.208	24.671	**4.737**	Lpin1	lipin 1
38780	A_55_P2045581	0.109	0.516	**4.733**	Olfr901	olfactory receptor 901
44248	A_55_P2103603	0.220	1.032	**4.695**	Gm831	predicted gene 831
30764	A_55_P1971259	0.102	0.464	**4.562**	1700022I11Rik	RIKEN cDNA 1700022I11 gene
17284	A_51_P160787	0.123	0.560	**4.555**	Art5	ADP-ribosyltransferase 5
14282	A_30_P01031706	0.146	0.653	**4.473**		
9435	A_30_P01026859	0.252	1.126	**4.473**		
12654	A_30_P01030078	0.140	0.623	**4.467**		
47406	A_55_P2141841	0.091	0.403	**4.442**		
28266	A_52_P84027	3.497	15.452	**4.418**	Cyp7a1	cytochrome P450, family 7, subfamily a, polypeptide 1
20675	A_51_P367866	3.167	13.733	**4.336**	Egr1	early growth response 1
32157	A_55_P1983553	0.086	0.371	**4.328**	AF529169	cDNA sequence AF529169
12513	A_30_P01029937	0.098	0.422	**4.284**		
45197	A_55_P2114626	0.104	0.446	**4.277**		
14912	A_30_P01032336	0.172	0.730	**4.252**		
30463	A_55_P1968723	6.932	29.296	**4.226**	Ccrn4l	CCR4 carbon catabolite repression 4-like (S. cerevisiae)
54498	A_66_P116250	0.176	0.739	**4.205**	Efhd2	EF hand domain containing 2
33864	A_55_P1999162	0.089	0.374	**4.204**		
43417	A_55_P2094277	0.101	0.414	**4.085**	Gm5447	predicted gene 5447
45667	A_55_P2120308	0.111	0.453	**4.071**	Cacna1a	calcium channel, voltage-dependent, P/Q type, alpha 1A subunit
24264	A_52_P213889	0.781	3.153	**4.037**	Tmc7	transmembrane channel-like gene family 7
13845	A_30_P01031269	0.086	0.346	**4.026**		
33892	A_55_P1999344	0.119	0.477	**4.012**		
40585	A_55_P2064063	0.206	0.823	**3.993**	Casp14	caspase 14
54	A_30_P01017478	0.138	0.545	**3.947**		
45273	A_55_P2115330	0.615	2.359	**3.838**	Nrg4	neuregulin 4
54556	A_66_P117450	0.080	0.304	**3.800**	Olfr1317	olfactory receptor 1317
43619	A_55_P2096422	0.403	1.528	**3.791**	Inhbb	inhibin beta-B
29168	A_55_P1957922	3.854	14.598	**3.788**	Arrdc2	arrestin domain containing 2
30444	A_55_P1968593	0.103	0.390	**3.786**	Olfr1505	olfactory receptor 1505

55669	A_66_P140742	0.078	0.293	**3.747**	Adc	arginine decarboxylase
50793	A_55_P2185900	1.347	5.041	**3.743**	Nrg4	neuregulin 4
42906	A_55_P2088440	4.957	18.449	**3.722**	Arrdc2	arrestin domain containing 2
23716	A_52_P14456	0.222	0.826	**3.719**	Srrm4	serine/arginine repetitive matrix 4
31015	A_55_P1973352	23.072	85.464	**3.704**		
55459	A_66_P136186	0.646	2.384	**3.691**	Wee1	WEE 1 homolog 1 (S. pombe)
3590	A_30_P01021014	0.224	0.803	**3.590**		
36352	A_55_P2021953	0.122	0.429	**3.524**	Olfr700	olfactory receptor 700
9186	A_30_P01026610	0.174	0.609	**3.496**		
46029	A_55_P2124582	0.104	0.361	**3.487**	Xlr4a	X-linked lymphocyte-regulated 4A
8226	A_30_P01025650	0.084	0.288	**3.433**		
2809	A_30_P01020233	0.116	0.399	**3.430**		
37112	A_55_P2029176	0.139	0.475	**3.421**	Atr	ataxia telangiectasia and Rad3 related
4475	A_30_P01021899	0.139	0.474	**3.412**		
43361	A_55_P2093705	0.191	0.647	**3.395**	Meig1	meiosis expressed gene 1
7819	A_30_P01025243	0.079	0.268	**3.389**		
49059	A_55_P2162557	0.120	0.406	**3.386**	Txndc9	thioredoxin domain containing 9
28482	A_55_P1952379	6.474	21.469	**3.316**	Fkbp5	FK506 binding protein 5
12670	A_30_P01030094	0.162	0.537	**3.310**		
51772	A_55_P2269673	0.189	0.624	**3.298**	Cabp1	calcium binding protein 1
27468	A_52_P608322	0.860	2.817	**3.276**	Maff	v-maf musculoaponeurotic fibrosarcoma oncogene family, protein F (avian)
7431	A_30_P01024855	0.142	0.460	**3.234**		
9904	A_30_P01027328	0.148	0.478	**3.225**		
14805	A_30_P01032229	0.085	0.274	**3.218**		
41845	A_55_P2076861	0.220	0.706	**3.208**	Sema6d	sema domain, transmembrane domain (TM), and cytoplasmic domain, (semaphorin) 6D
44064	A_55_P2101508	0.119	0.381	**3.192**	Fgf8	fibroblast growth factor 8
42367	A_55_P2082658	0.448	1.428	**3.187**	LOC102633223	uncharacterized LOC102633223
24421	A_52_P230167	0.079	0.251	**3.187**	Setbp1	SET binding protein 1
25500	A_52_P366525	1.470	4.682	**3.185**	Coq10b	coenzyme Q10 homolog B (S. cerevisiae)
23653	A_52_P137371	0.766	2.429	**3.171**	Hmgcr	3-hydroxy-3-methylglutaryl-Coenzyme A reductase
36410	A_55_P2022524	0.113	0.356	**3.140**	Rbpj	recombination signal binding protein for immunoglobulin kappa J region
12728	A_30_P01030152	0.136	0.423	**3.116**		
44445	A_55_P2106039	0.328	1.019	**3.111**	Map3k6	mitogen-activated protein kinase kinase kinase 6
17198	A_51_P155873	1.627	5.040	**3.098**	Ppp1r3g	protein phosphatase 1, regulatory (inhibitor) subunit 3G
53947	A_66_P105497	0.238	0.724	**3.050**	Gm5429	predicted gene 5429

52817	A_55_P2372325	0.129	0.392	**3.045**	Zim3	zinc finger, imprinted 3
30613	A_55_P1970033	8.796	26.772	**3.044**	Per1	period circadian clock 1
24828	A_52_P278497	11.296	34.380	**3.044**	Eif4ebp3	eukaryotic translation initiation factor 4E binding protein 3
10534	A_30_P01027958	0.245	0.744	**3.032**		
17584	A_51_P179531	0.215	0.648	**3.017**	Flt4	FMS-like tyrosine kinase 4
42997	A_55_P2089488	2.705	8.132	**3.006**	Coq10b	coenzyme Q10 homolog B (S. cerevisiae)
23551	A_52_P122822	0.179	0.538	**3.002**	Wdfy2	WD repeat and FYVE domain containing 2
45187	A_55_P2114498	3.038	9.023	**2.970**	Gm8801	protein phosphatase 1, regulatory subunit 10 pseudogene
37618	A_55_P2034067	0.198	0.585	**2.959**	Myom3	myomesin family, member 3
2585	A_30_P01020009	0.378	1.119	**2.957**		
28847	A_55_P1955300	0.281	0.828	**2.951**	Gm4969	predicted gene 4969
14973	A_30_P01032397	0.086	0.255	**2.950**		
37288	A_55_P2030899	4.112	12.108	**2.945**		
20227	A_51_P340829	0.443	1.303	**2.940**	AA986860	expressed sequence AA986860
13496	A_30_P01030920	0.128	0.375	**2.930**		
19236	A_51_P279437	8.326	24.320	**2.921**	Mfsd2a	major facilitator superfamily domain containing 2A
21650	A_51_P424959	0.148	0.432	**2.918**	Bcl6b	B cell CLL/lymphoma 6, member B
42460	A_55_P2083649	154.035	447.820	**2.907**	Alas1	aminolevulinic acid synthase 1
23974	A_52_P177271	0.166	0.481	**2.897**	Mtl5	metallothionein-like 5, testis-specific (tesmin)
24846	A_52_P281659	0.225	0.651	**2.893**	Klf13	Kruppel-like factor 13
33962	A_55_P1999888	0.092	0.263	**2.867**	Chst4	carbohydrate (chondroitin 6/keratan) sulfotransferase 4
2512	A_30_P01019936	0.292	0.835	**2.862**		
47020	A_55_P2136906	0.120	0.342	**2.858**	Vpreb3	pre-B lymphocyte gene 3
30021	A_55_P1964896	0.132	0.377	**2.848**	Gm3014	predicted gene 3014
28066	A_52_P683336	0.121	0.343	**2.847**	AY074887	cDNA sequence AY074887
18758	A_51_P249286	19.014	53.789	**2.829**	Rgs16	regulator of G-protein signaling 16
33230	A_55_P1993180	0.146	0.410	**2.804**	Speer4c	spermatogenesis associated glutamate (E)-rich protein 4c
23012	A_51_P505662	0.118	0.327	**2.776**	Noxred1	NADP+ dependent oxidoreductase domain containing 1
51870	A_55_P2278093	0.116	0.320	**2.751**	AI480461	expressed sequence AI480461
17443	A_51_P170807	0.359	0.982	**2.735**	LOC102641377	uncharacterized LOC102641377
35087	A_55_P2009813	0.106	0.289	**2.732**	Gm5751	predicted gene 5751
41064	A_55_P2068892	24.893	68.000	**2.732**	Il6ra	interleukin 6 receptor, alpha
34309	A_55_P2002988	0.127	0.345	**2.719**	Usp17ld	ubiquitin specific peptidase 17-like D
39780	A_55_P2055607	0.280	0.756	**2.700**	Enpp1	ectonucleotide pyrophosphatase/phosphodiesterase 1
27818	A_52_P651248	0.081	0.218	**2.699**	Zdhhc2	zinc finger, DHHC domain containing 2
38842	A_55_P2046101	0.339	0.914	**2.697**	Xlr4b	X-linked lymphocyte-regulated 4B

7083	A_30_P01024507	0.091	0.244	**2.691**		
27952	A_52_P669922	126.857	340.488	**2.684**	Hamp	hepcidin antimicrobial peptide
39751	A_55_P2055316	0.423	1.134	**2.682**		
21686	A_51_P427530	0.194	0.520	**2.675**	Pgm1	phosphoglucomutase 1
31357	A_55_P1976351	4.321	11.438	**2.647**	Gpcpd1	glycerophosphocholine phosphodiesterase GDE1 homolog (S. cerevisiae)
52553	A_55_P2345776	0.109	0.288	**2.639**	N4bp2l1	NEDD4 binding protein 2-like 1
33403	A_55_P1994633	0.120	0.316	**2.635**	Gm711	predicted gene 711
53063	A_55_P2399499	0.106	0.278	**2.634**	B930025P03Rik	RIKEN cDNA B930025P03 gene
30148	A_55_P1966029	0.582	1.524	**2.617**	A930033H14Rik	RIKEN cDNA A930033H14 gene
50532	A_55_P2182452	0.200	0.521	**2.607**	Tnfsf15	tumor necrosis factor (ligand) superfamily, member 15
50794	A_55_P2185905	0.997	2.589	**2.597**	Nrg4	neuregulin 4
27944	A_52_P668478	0.337	0.868	**2.572**	Rhbdd2	rhomboid domain containing 2
51721	A_55_P2262753	1.406	3.601	**2.561**	9530053J19Rik	RIKEN cDNA 9530053J19 gene
38176	A_55_P2039622	0.103	0.264	**2.558**	Clec4a2	C-type lectin domain family 4, member a2
38717	A_55_P2044982	0.308	0.787	**2.556**	Zfp74	zinc finger protein 74
52758	A_55_P2367463	0.127	0.323	**2.549**	Acer2	alkaline ceramidase 2
41380	A_55_P2072080	0.139	0.353	**2.545**	Zfp142	zinc finger protein 142
17218	A_51_P157042	1.714	4.350	**2.538**	Ctgf	connective tissue growth factor
16270	A_51_P100991	0.165	0.419	**2.534**	Gucy2c	guanylate cyclase 2c
1684	A_30_P01019108	0.267	0.674	**2.526**		
10328	A_30_P01027752	0.210	0.524	**2.495**		
13454	A_30_P01030878	0.118	0.294	**2.489**		
42484	A_55_P2083919	0.141	0.351	**2.483**	Robo2	roundabout homolog 2 (Drosophila)
27771	A_52_P64707	3.854	9.549	**2.478**	Foxa3	forkhead box A3
22021	A_51_P447976	1.367	3.377	**2.471**	Fam46c	family with sequence similarity 46, member C
25850	A_52_P409675	0.296	0.730	**2.470**	Wdr11	WD repeat domain 11
37147	A_55_P2029574	67.740	166.350	**2.456**	Por	P450 (cytochrome) oxidoreductase
17524	A_51_P175580	9.175	22.504	**2.453**	Trp53inp1	transformation related protein 53 inducible nuclear protein 1
6741	A_30_P01024165	0.071	0.173	**2.446**		
9377	A_30_P01026801	0.105	0.256	**2.446**		
41975	A_55_P2078246	0.659	1.611	**2.445**	Ppp1r10	protein phosphatase 1, regulatory subunit 10
11372	A_30_P01028796	0.253	0.618	**2.440**		
10545	A_30_P01027969	0.083	0.202	**2.435**		
54689	A_66_P120125	1.050	2.555	**2.434**	Nrep	neuronal regeneration related protein
22613	A_51_P483473	35.737	86.935	**2.433**	St3gal5	ST3 beta-galactoside alpha-2,3-sialyltransferase 5
46030	A_55_P2124586	0.339	0.824	**2.432**	Xlr4a	X-linked lymphocyte-regulated 4A

30042	A_55_P1965101	1.490	3.621	**2.430**	Xlr3b	X-linked lymphocyte-regulated 3B
1984	A_30_P01019408	0.113	0.273	**2.418**		
53051	A_55_P2398270	0.081	0.195	**2.414**	5033423O07Rik	RIKEN cDNA 5033423O07 gene
52217	A_55_P2312654	0.247	0.595	**2.411**	BB070754	expressed sequence BB070754
39466	A_55_P2052425	7.359	17.716	**2.407**	Setd4	SET domain containing 4
201	A_30_P01017625	1.038	2.482	**2.390**		
27896	A_52_P661071	5.452	12.965	**2.378**	Snhg3	small nucleolar RNA host gene 3
29396	A_55_P1959683	0.673	1.597	**2.374**	Xlr3b	X-linked lymphocyte-regulated 3B
20187	A_51_P338443	80.499	189.656	**2.356**	Angptl4	angiopoietin-like 4
14250	A_30_P01031674	0.110	0.259	**2.356**		
18833	A_51_P254646	0.399	0.939	**2.353**	Jdp2	Jun dimerization protein 2
48150	A_55_P2150811	0.070	0.164	**2.351**	6330405D24Rik	RIKEN cDNA 6330405D24 gene
43304	A_55_P2092989	1.117	2.623	**2.349**		
25144	A_52_P319438	0.289	0.678	**2.344**	Ankrd37	ankyrin repeat domain 37
32843	A_55_P1989653	0.100	0.234	**2.343**	Slco4a1	solute carrier organic anion transporter family, member 4a1
16464	A_51_P112308	3.185	7.461	**2.342**	1810011O10Rik	RIKEN cDNA 1810011O10 gene
44450	A_55_P2106074	0.104	0.243	**2.339**	Wfikkn1	WAP, FS, Ig, KU, and NTR-containing protein 1
17139	A_51_P152990	2.395	5.558	**2.321**	Grem2	gremlin 2 homolog, cysteine knot superfamily (Xenopus laevis)
30532	A_55_P1969311	0.100	0.231	**2.316**	Gramd1b	GRAM domain containing 1B
29426	A_55_P1959985	4.175	9.637	**2.308**	Alas1	aminolevulinic acid synthase 1
52139	A_55_P2303310	0.369	0.851	**2.305**	C730036E19Rik	RIKEN cDNA C730036E19 gene
16835	A_51_P135037	0.097	0.223	**2.303**	Preb	prolactin regulatory element binding
20081	A_51_P331870	0.756	1.740	**2.303**	Rnf145	ring finger protein 145
16288	A_51_P102257	0.907	2.088	**2.302**	Tns1	tensin 1
39356	A_55_P2051322	6.049	13.780	**2.278**	Efhd2	EF hand domain containing 2
31620	A_55_P1978770	0.386	0.878	**2.274**	E030018B13Rik	RIKEN cDNA E030018B13 gene
21971	A_51_P444447	8.250	18.710	**2.268**	Cebpd	CCAAT/enhancer binding protein (C/EBP), delta
18232	A_51_P219483	0.798	1.807	**2.264**	Tsku	tsukushi
41159	A_55_P2069907	0.098	0.221	**2.258**	Acot3	acyl-CoA thioesterase 3
41773	A_55_P2076057	0.084	0.188	**2.253**	Hmga1	high mobility group AT-hook 1
12816	A_30_P01030240	0.207	0.462	**2.236**		
49412	A_55_P2167269	4.831	10.769	**2.229**	Pcsk4	proprotein convertase subtilisin/kexin type 4
42469	A_55_P2083806	0.133	0.297	**2.229**	Sp8	trans-acting transcription factor 8
45816	A_55_P2122020	0.530	1.179	**2.223**	Klf4	Kruppel-like factor 4 (gut)
19632	A_51_P304163	0.089	0.197	**2.220**	Serac1	serine active site containing 1
55590	A_66_P139159	0.075	0.167	**2.214**	Hras	Harvey rat sarcoma virus oncogene

46974	A_55_P2136410	0.129	0.286	**2.211**		
54140	A_66_P109183	0.251	0.554	**2.210**	Apold1	apolipoprotein L domain containing 1
37731	A_55_P2035315	4.348	9.595	**2.207**	Rasgef1b	RasGEF domain family, member 1B
6341	A_30_P01023765	0.135	0.299	**2.205**		
41144	A_55_P2069768	0.090	0.199	**2.205**	Kiss1	KiSS-1 metastasis-suppressor
22101	A_51_P453043	16.242	35.654	**2.195**	Aacs	acetoacetyl-CoA synthetase
14334	A_30_P01031758	0.082	0.179	**2.189**		
47788	A_55_P2146297	0.116	0.254	**2.183**	Lmx1b	LIM homeobox transcription factor 1 beta
33485	A_55_P1995427	0.094	0.206	**2.180**	Nxf2	nuclear RNA export factor 2
19825	A_51_P315904	11.922	25.986	**2.180**	Gadd45g	growth arrest and DNA-damage-inducible 45 gamma
47701	A_55_P2145262	0.073	0.158	**2.179**	Pex5l	peroxisomal biogenesis factor 5-like
52297	A_55_P2321453	0.291	0.634	**2.177**	D11Ertd726e	DNA segment, Chr 11, ERATO Doi 726, expressed
9482	A_30_P01026906	0.097	0.210	**2.172**		
24346	A_52_P222230	28.785	62.485	**2.171**		
18694	A_51_P245796	9.409	20.359	**2.164**	Ddit4	DNA-damage-inducible transcript 4
1823	A_30_P01019247	0.085	0.184	**2.162**		
16190	A_30_P01033614	0.254	0.549	**2.161**		
13564	A_30_P01030988	1.204	2.596	**2.157**		
17587	A_51_P179701	0.157	0.337	**2.154**	Hlx	H2.0-like homeobox
27915	A_52_P663526	1.121	2.404	**2.144**	Nmrk1	nicotinamide riboside kinase 1
18098	A_51_P210956	2.282	4.894	**2.144**	Vcam1	vascular cell adhesion molecule 1
13807	A_30_P01031231	0.105	0.226	**2.141**		
37656	A_55_P2034475	0.123	0.264	**2.138**	Serpinb7	serine (or cysteine) peptidase inhibitor, clade B, member 7
47810	A_55_P2146590	0.274	0.585	**2.137**	1810011O10Rik	RIKEN cDNA 1810011O10 gene
1250	A_30_P01018674	0.085	0.182	**2.133**		
22886	A_51_P498388	1.257	2.676	**2.128**	Sbk1	SH3-binding kinase 1
16997	A_51_P144264	2.871	6.093	**2.123**	Klf2	Kruppel-like factor 2 (lung)
37340	A_55_P2031471	0.116	0.246	**2.120**	Rufy4	RUN and FYVE domain containing 4
26700	A_52_P516034	4.458	9.439	**2.117**	Ptp4a1	protein tyrosine phosphatase 4a1
52805	A_55_P2371281	0.164	0.347	**2.114**	9030601B04Rik	RIKEN cDNA 9030601B04 gene
35027	A_55_P2009196	0.463	0.978	**2.114**	Gm5441	predicted gene 5441
47812	A_55_P2146636	0.094	0.198	**2.111**		
12121	A_30_P01029545	0.307	0.648	**2.107**		
20573	A_51_P361448	0.245	0.516	**2.107**	Scara5	scavenger receptor class A, member 5 (putative)
47821	A_55_P2146683	0.089	0.188	**2.105**	Gm4657	predicted gene 4657
3510	A_30_P01020934	0.398	0.837	**2.105**		

50816	A_55_P2186220	0.079	0.167	**2.104**		
23881	A_52_P163939	0.275	0.577	**2.101**	Cln6	ceroid-lipofuscinosis, neuronal 6
17340	A_51_P163476	0.117	0.246	**2.093**	5430403G16Rik	RIKEN cDNA 5430403G16 gene
35690	A_55_P2015753	10.243	21.382	**2.087**	Enho	energy homeostasis associated
18895	A_51_P258493	0.125	0.261	**2.087**	Per3	period circadian clock 3
26336	A_52_P474814	0.164	0.343	**2.085**	4921506M07Rik	RIKEN cDNA 4921506M07 gene
19288	A_51_P282760	0.348	0.723	**2.076**	Per2	period circadian clock 2
30009	A_55_P1964752	0.106	0.220	**2.073**	Slc23a3	solute carrier family 23 (nucleobase transporters), member 3
4766	A_30_P01022190	0.168	0.348	**2.069**		
429	A_30_P01017853	0.177	0.366	**2.068**		
49178	A_55_P2164075	1.065	2.198	**2.064**	Gm10804	predicted gene 10804
38960	A_55_P2047215	0.097	0.199	**2.054**	Slc37a4	solute carrier family 37 (glucose-6-phosphate transporter), member 4
37132	A_55_P2029420	0.107	0.220	**2.053**	2610318N02Rik	RIKEN cDNA 2610318N02 gene
7698	A_30_P01025122	0.317	0.649	**2.050**		
39336	A_55_P2051159	26.955	55.117	**2.045**	Upp2	uridine phosphorylase 2
50705	A_55_P2184811	0.407	0.831	**2.043**	Eif2s1	eukaryotic translation initiation factor 2, subunit 1 alpha
18779	A_51_P250445	2.834	5.785	**2.042**	Zfp276	zinc finger protein (C2H2 type) 276
24660	A_52_P257625	0.461	0.940	**2.038**	Esm1	endothelial cell-specific molecule 1
22170	A_51_P458384	17.960	36.585	**2.037**	Slc38a2	solute carrier family 38, member 2
21938	A_51_P442097	0.452	0.919	**2.036**	Slc41a3	solute carrier family 41, member 3
48911	A_55_P2160623	0.078	0.159	**2.033**	Kalrn	kalirin, RhoGEF kinase
53045	A_55_P2397854	0.138	0.281	**2.032**	4921518K17Rik	RIKEN cDNA 4921518K17 gene
23752	A_52_P150114	0.118	0.239	**2.030**		
10405	A_30_P01027829	0.168	0.341	**2.030**		
44212	A_55_P2103097	0.144	0.292	**2.026**	Ccr8	chemokine (C-C motif) receptor 8
18784	A_51_P250807	4.357	8.817	**2.024**	Spata2l	spermatogenesis associated 2-like
42227	A_55_P2081164	0.554	1.121	**2.022**	Cd300lf	CD300 antigen like family member F
46386	A_55_P2129309	2.441	4.935	**2.022**	LOC102636048	uncharacterized LOC102636048
41143	A_55_P2069765	0.350	0.707	**2.019**	Kiss1	KiSS-1 metastasis-suppressor
8737	A_30_P01026161	0.371	0.747	**2.017**		
40606	A_55_P2064333	0.384	0.775	**2.016**	Lama3	laminin, alpha 3
39200	A_55_P2049513	0.204	0.411	**2.012**		
34042	A_55_P2000595	0.106	0.212	**2.011**		
51815	A_55_P2274378	1.963	3.946	**2.010**	AW549542	expressed sequence AW549542
48353	A_55_P2153517	17.479	35.100	**2.008**	Enho	energy homeostasis associated
46097	A_55_P2125613	0.111	0.222	**2.005**	Fam71f2	family with sequence similarity 71, member F2

Microarray data_Up466

Narabi_55681	ProbeName	LC(normalized)	LV(normalized)	Fold change	GeneSymbol	GeneName
36561	A_55_P2024066	0.014	1.447	**102.794**	Smr3a	submaxillary gland androgen regulated protein 3A
51413	A_55_P2235832	0.017	1.730	**101.706**	Gm10278	predicted gene 10278
12097	A_30_P01029521	0.014	1.325	**95.213**		
35146	A_55_P2010366	0.014	1.287	**93.217**	Wdr95	WD40 repeat domain 95
4094	A_30_P01021518	0.015	1.153	**75.690**		
15749	A_30_P01033173	0.016	1.197	**72.833**		
49859	A_55_P2173233	0.015	1.021	**66.618**	Galntl5	UDP-N-acetyl-alpha-D-galactosamine:polypeptide N-acetylgalactosaminyltransferase-like 5
27618	A_52_P628212	0.017	1.141	**66.195**	Esrrb	estrogen related receptor, beta
39514	A_55_P2052913	0.018	0.997	**54.872**		
10338	A_30_P01027762	0.018	0.882	**48.504**		
31342	A_55_P1976142	0.017	0.829	**48.336**	Gm10609	predicted gene 10609
11204	A_30_P01028628	0.016	0.678	**42.384**		
4258	A_30_P01021682	0.015	0.636	**42.334**		
21343	A_51_P406796	0.018	0.744	**42.321**	Sult4a1	sulfotransferase family 4A, member 1
27637	A_52_P630463	0.017	0.713	**42.119**	Lce1g	late cornified envelope 1G
43458	A_55_P2094771	0.016	0.651	**41.189**	Olfr32	olfactory receptor 32
14982	A_30_P01032406	0.018	0.715	**40.654**		
3085	A_30_P01020509	0.014	0.569	**40.465**		
42995	A_55_P2089472	0.015	0.608	**40.142**	1700020A23Rik	RIKEN cDNA 1700020A23 gene
29058	A_55_P1957178	0.016	0.624	**40.030**	4930547H16Rik	RIKEN cDNA 4930547H16 gene
42865	A_55_P2088033	0.015	0.588	**38.687**	Gm6904	predicted gene 6904
32636	A_55_P1987800	0.017	0.629	**37.001**		
12167	A_30_P01029591	0.018	0.638	**36.418**		
28730	A_55_P1954384	0.019	0.682	**36.411**	Spin2d	spindlin family, member 2D
23801	A_52_P154858	0.015	0.502	**34.287**	A730049H05Rik	RIKEN cDNA A730049H05 gene
3529	A_30_P01020953	0.015	0.515	**34.031**		
52070	A_55_P2295856	0.016	0.530	**33.960**	AI594671	expressed sequence AI594671
12344	A_30_P01029768	0.014	0.491	**33.924**		
4536	A_30_P01021960	0.013	0.441	**32.968**		

15800	A_30_P01033224	0.014	0.471	**32.742**		
5940	A_30_P01023364	0.016	0.518	**32.251**		
32521	A_55_P1986823	0.014	0.439	**32.206**	Gm8075	predicted gene 8075
10760	A_30_P01028184	0.017	0.563	**32.193**		
38523	A_55_P2043127	0.014	0.442	**32.160**	Gm3045	predicted gene 3045
4935	A_30_P01022359	0.016	0.498	**31.834**		
52051	A_55_P2294273	0.013	0.421	**31.574**	C030006F08Rik	RIKEN cDNA C030006F08 gene
1163	A_30_P01018587	0.015	0.462	**30.854**		
45493	A_55_P2118081	0.018	0.532	**29.923**	Gm12171	predicted gene 12171
31923	A_55_P1981351	0.014	0.416	**29.601**	Vmn2r114	vomeronasal 2, receptor 114
10598	A_30_P01028022	0.016	0.485	**29.496**		
51037	A_55_P2200034	0.017	0.497	**29.226**	Dleu2	deleted in lymphocytic leukemia, 2
6820	A_30_P01024244	0.014	0.390	**28.306**		
52677	A_55_P2359985	0.030	0.847	**28.151**	3010033K07Rik	RIKEN cDNA 3010033K07 gene
6581	A_30_P01024005	0.017	0.484	**28.106**		
48930	A_55_P2160810	0.024	0.674	**28.052**	Foxd2	forkhead box D2
3952	A_30_P01021376	0.025	0.696	**27.993**		
6440	A_30_P01023864	0.017	0.477	**27.936**		
9265	A_30_P01026689	0.015	0.409	**27.663**		
55456	A_66_P136129	0.014	0.398	**27.527**	Gm7134	kinesin family member 18B pseudogene
175	A_30_P01017599	0.014	0.396	**27.506**		
1567	A_30_P01018991	0.014	0.386	**27.504**		
3003	A_30_P01020427	0.014	0.381	**27.385**		
9787	A_30_P01027211	0.014	0.387	**26.818**		
249	A_30_P01017673	0.013	0.353	**26.723**		
7847	A_30_P01025271	0.015	0.394	**26.393**		
27617	A_52_P628171	0.034	0.901	**26.274**	Nfatc2	nuclear factor of activated T cells, cytoplasmic, calcineurin dependent 2
43416	A_55_P2094272	0.023	0.584	**25.663**	Gm9694	predicted gene 9694
15833	A_30_P01033257	0.017	0.426	**25.659**		
43644	A_55_P2096742	0.015	0.384	**25.587**	Noto	notochord homolog (Xenopus laevis)
39216	A_55_P2049717	0.018	0.451	**25.487**	Agrp	agouti related protein
35967	A_55_P2018215	0.014	0.361	**25.385**		
48481	A_55_P2155264	0.014	0.353	**25.116**		
53488	A_65_P05456	0.017	0.420	**24.846**	Naa11	N(alpha)-acetyltransferase 11, NatA catalytic subunit
36656	A_55_P2024948	0.016	0.396	**24.324**	Hoxb8	homeobox B8
14718	A_30_P01032142	0.014	0.352	**24.307**		

42027	A_55_P2078795	0.020	0.482	**23.771**	Olfr55	olfactory receptor 55
23340	A_52_P1027874	0.019	0.451	**23.671**	A230083G16Rik	RIKEN cDNA A230083G16 gene
22590	A_51_P482148	0.018	0.434	**23.620**	Mecom	MDS1 and EVI1 complex locus
2144	A_30_P01019568	0.039	0.911	**23.544**		
33634	A_55_P1997110	0.014	0.327	**23.489**	Tmprss12	transmembrane (C-terminal) protease, serine 12
3069	A_30_P01020493	0.016	0.374	**23.424**		
43095	A_55_P2090733	0.016	0.382	**23.255**		
20865	A_51_P379083	0.018	0.416	**23.068**	Adam24	a disintegrin and metallopeptidase domain 24 (testase 1)
7032	A_30_P01024456	0.016	0.364	**22.998**		
5108	A_30_P01022532	0.020	0.445	**22.735**		
28839	A_55_P1955263	0.015	0.348	**22.656**		
39225	A_55_P2049835	0.014	0.320	**22.442**	Nxph1	neurexophilin 1
22044	A_51_P449233	0.017	0.386	**22.111**	Thsd4	thrombospondin, type I, domain containing 4
1753	A_30_P01019177	0.016	0.340	**21.865**		
20083	A_51_P332003	0.017	0.370	**21.684**	Aste1	asteroid homolog 1 (Drosophila)
34121	A_55_P2001267	0.014	0.299	**21.654**		
18024	A_51_P207380	0.017	0.369	**21.125**	Lkaaear1	LKAAEAR motif containing 1 (IKAAEAR murine motif)
34054	A_55_P2000688	0.014	0.289	**21.048**	Gm6958	predicted gene 6958
50672	A_55_P2184345	0.013	0.278	**20.811**	Gm7008	predicted gene 7008
19584	A_51_P301603	0.022	0.464	**20.713**	AI854517	expressed sequence AI854517
25884	A_52_P413394	0.015	0.304	**20.411**	Ppef2	protein phosphatase, EF hand calcium-binding domain 2
20014	A_51_P328001	0.015	0.299	**20.381**	Sftpc	surfactant associated protein C
22818	A_51_P494293	0.016	0.329	**20.051**	9130024F11Rik	RIKEN cDNA 9130024F11 gene
13646	A_30_P01031070	0.019	0.369	**19.536**		
5569	A_30_P01022993	0.031	0.600	**19.425**		
12583	A_30_P01030007	0.013	0.262	**19.398**		
1415	A_30_P01018839	0.018	0.341	**19.396**		
7234	A_30_P01024658	0.016	0.300	**19.321**		
37637	A_55_P2034311	0.017	0.329	**19.039**		
6837	A_30_P01024261	0.025	0.475	**19.021**		
46793	A_55_P2133997	0.023	0.431	**18.903**	Abca12	ATP-binding cassette, sub-family A (ABC1), member 12
13425	A_30_P01030849	0.018	0.344	**18.873**		
6189	A_30_P01023613	0.017	0.330	**18.873**		
47772	A_55_P2146111	0.014	0.273	**18.871**	Spink11	serine peptidase inhibitor, Kazal type 11
3818	A_30_P01021242	0.018	0.345	**18.737**		
31220	A_55_P1975115	0.017	0.315	**18.649**		

29611	A_55_P1961433	0.017	0.318	**18.597**		
17757	A_51_P190961	0.016	0.290	**18.596**	Gzmf	granzyme F
2940	A_30_P01020364	0.014	0.259	**18.338**		
26932	A_52_P545003	0.017	0.315	**18.240**	Cep72	centrosomal protein 72
8917	A_30_P01026341	0.019	0.337	**18.127**		
3585	A_30_P01021009	0.016	0.289	**17.946**		
35566	A_55_P2014460	0.013	0.238	**17.629**	Bdkrb1	bradykinin receptor, beta 1
14023	A_30_P01031447	0.015	0.260	**17.623**		
7578	A_30_P01025002	0.026	0.464	**17.566**		
13139	A_30_P01030563	0.017	0.303	**17.538**		
29128	A_55_P1957669	0.016	0.282	**17.489**	Defb5	defensin beta 5
49714	A_55_P2171066	0.018	0.307	**17.483**		
29817	A_55_P1963154	0.016	0.279	**17.373**	Folh1	folate hydrolase 1
9636	A_30_P01027060	0.015	0.255	**17.264**		
8799	A_30_P01026223	0.016	0.282	**17.224**		
10627	A_30_P01028051	0.014	0.235	**17.153**		
16788	A_51_P131840	0.016	0.269	**17.124**	Cst12	cystatin 12
23737	A_52_P148212	0.015	0.253	**17.115**	Mis18bp1	MIS18 binding protein 1
10182	A_30_P01027606	0.014	0.245	**17.025**		
6702	A_30_P01024126	0.015	0.252	**16.955**		
51032	A_55_P2199737	0.020	0.343	**16.891**	Dlgap2	discs, large (Drosophila) homolog-associated protein 2
1619	A_30_P01019043	0.018	0.299	**16.850**		
41126	A_55_P2069632	0.014	0.229	**16.606**	Olfr874	olfactory receptor 874
53707	A_66_P100496	0.021	0.347	**16.570**	Speer4b	spermatogenesis associated glutamate (E)-rich protein 4b
43343	A_55_P2093528	0.016	0.260	**16.519**		
42185	A_55_P2080733	0.025	0.411	**16.489**	Rhox3f	reproductive homeobox 3F
5741	A_30_P01023165	0.014	0.233	**16.481**		
28991	A_55_P1956652	0.016	0.259	**16.394**	Sfmbt2	Scm-like with four mbt domains 2
55260	A_66_P132082	0.014	0.225	**16.326**		
51884	A_55_P2279250	0.014	0.231	**16.273**	D8Ertd503e	DNA segment, Chr 8, ERATO Doi 503, expressed
40934	A_55_P2067632	0.018	0.283	**16.191**	Ush1c	Usher syndrome 1C
34194	A_55_P2002050	0.016	0.258	**16.122**	Scgb2b24	secretoglobin, family 2B, member 24
6528	A_30_P01023952	0.016	0.250	**16.117**		
24044	A_52_P186349	0.014	0.230	**15.958**	Gm6744	predicted gene 6744
12879	A_30_P01030303	0.017	0.266	**15.890**		
6262	A_30_P01023686	0.018	0.279	**15.876**		

45330	A_55_P2116094	0.045	0.716	**15.876**	Gsc2	goosecoid homebox 2
24712	A_52_P2645	0.016	0.260	**15.853**	Rftn2	raftlin family member 2
15275	A_30_P01032699	0.019	0.308	**15.824**		
28046	A_52_P680751	0.030	0.478	**15.768**	Cux1	cut-like homeobox 1
30220	A_55_P1966644	0.014	0.216	**15.722**	Trpc6	transient receptor potential cation channel, subfamily C, member 6
48002	A_55_P2148931	0.014	0.213	**15.520**	A730017C20Rik	RIKEN cDNA A730017C20 gene
4979	A_30_P01022403	0.015	0.227	**15.511**		
18990	A_51_P264084	0.017	0.269	**15.427**	Rab36	RAB36, member RAS oncogene family
20563	A_51_P360586	0.016	0.247	**15.335**	Hoxa6	homeobox A6
21446	A_51_P413597	0.014	0.209	**15.259**	Ranbp17	RAN binding protein 17
16445	A_51_P111068	0.017	0.258	**15.235**	Olfr478	olfactory receptor 478
4301	A_30_P01021725	0.014	0.213	**15.146**		
29371	A_55_P1959521	0.018	0.266	**15.095**	Etv4	ets variant gene 4 (E1A enhancer binding protein, E1AF)
14165	A_30_P01031589	0.015	0.220	**15.031**		
51525	A_55_P2244712	0.014	0.206	**14.929**	4933429O19Rik	RIKEN cDNA 4933429O19 gene
13526	A_30_P01030950	0.017	0.250	**14.918**		
29060	A_55_P1957188	0.016	0.238	**14.786**	Gm15104	predicted gene 15104
3772	A_30_P01021196	0.016	0.243	**14.768**		
31844	A_55_P1980699	0.013	0.196	**14.692**	Olfr624	olfactory receptor 624
45257	A_55_P2115216	0.016	0.230	**14.510**	Gm5622	predicted gene 5622
39030	A_55_P2047974	0.016	0.230	**14.290**		
8644	A_30_P01026068	0.017	0.244	**14.277**		
32625	A_55_P1987725	0.046	0.658	**14.206**	Gria4	glutamate receptor, ionotropic, AMPA4 (alpha 4)
12731	A_30_P01030155	0.016	0.232	**14.178**		
23952	A_52_P174721	0.017	0.243	**14.054**	Defb20	defensin beta 20
10019	A_30_P01027443	0.016	0.220	**13.958**		
55343	A_66_P133702	0.014	0.190	**13.920**	Gm12888	predicted gene 12888
28807	A_55_P1954990	0.014	0.197	**13.918**	Trpv1	transient receptor potential cation channel, subfamily V, member 1
15358	A_30_P01032782	0.016	0.229	**13.887**		
21055	A_51_P389834	0.013	0.182	**13.802**	Zpld1	zona pellucida like domain containing 1
48085	A_55_P2150013	0.017	0.240	**13.776**	Klra12	killer cell lectin-like receptor subfamily A, member 12
84	A_30_P01017508	0.049	0.675	**13.694**		
53766	A_66_P101664	0.019	0.263	**13.602**	Scai	suppressor of cancer cell invasion
6650	A_30_P01024074	0.016	0.212	**13.591**		
5972	A_30_P01023396	0.015	0.200	**13.566**		
12393	A_30_P01029817	0.017	0.228	**13.497**		

25	A_30_P01017449	0.013	0.180	**13.429**		
47875	A_55_P2147306	0.028	0.374	**13.383**	Robo3	roundabout homolog 3 (Drosophila)
367	A_30_P01017791	0.017	0.225	**13.296**		
52755	A_55_P2367350	0.017	0.226	**13.194**	4933403J19Rik	RIKEN cDNA 4933403J19 gene
43546	A_55_P2095716	0.026	0.340	**13.129**	Gnrh1	gonadotropin releasing hormone 1
623	A_30_P01018047	0.019	0.243	**13.056**		
11499	A_30_P01028923	0.015	0.189	**13.008**		
3165	A_30_P01020589	0.014	0.186	**12.897**		
16164	A_30_P01033588	0.016	0.203	**12.849**		
5307	A_30_P01022731	0.028	0.352	**12.654**		
6738	A_30_P01024162	0.016	0.198	**12.649**		
40961	A_55_P2067831	0.013	0.170	**12.648**	Grm1	glutamate receptor, metabotropic 1
30116	A_55_P1965767	0.013	0.170	**12.640**		
15132	A_30_P01032556	0.028	0.346	**12.519**		
8340	A_30_P01025764	0.013	0.162	**12.490**		
13293	A_30_P01030717	0.014	0.173	**12.348**		
5033	A_30_P01022457	0.036	0.437	**12.296**		
19063	A_51_P268603	0.017	0.210	**12.295**	Olfr881	olfactory receptor 881
4498	A_30_P01021922	0.027	0.327	**12.181**		
2245	A_30_P01019669	0.021	0.250	**12.179**		
55168	A_66_P130276	0.015	0.182	**12.095**		
40204	A_55_P2060155	0.014	0.169	**12.094**	Krtap4-6	keratin associated protein 4-6
47720	A_55_P2145511	0.015	0.179	**12.092**	LOC102639781	uncharacterized LOC102639781
3433	A_30_P01020857	0.015	0.180	**12.035**		
32810	A_55_P1989362	0.014	0.162	**11.927**	Gm14129	predicted gene 14129
44206	A_55_P2103011	0.077	0.922	**11.909**	Sema4d	sema domain, immunoglobulin domain (Ig), transmembrane domain (TM) and short cytoplasmic domain, (semaphorin) 4D
35461	A_55_P2013445	0.016	0.188	**11.858**	Obscn	obscurin, cytoskeletal calmodulin and titin-interacting RhoGEF
52356	A_55_P2326802	0.020	0.239	**11.842**		
3015	A_30_P01020439	0.014	0.170	**11.772**		
32590	A_55_P1987406	0.018	0.213	**11.725**		
11850	A_30_P01029274	0.027	0.315	**11.689**		
10541	A_30_P01027965	0.018	0.204	**11.604**		
23314	A_52_P1008476	0.014	0.159	**11.563**	Pou3f3	POU domain, class 3, transcription factor 3
54301	A_66_P112260	0.014	0.157	**11.540**	Gm10688	predicted gene 10688
32719	A_55_P1988526	0.013	0.153	**11.423**		

34790	A_55_P2007121	0.014	0.161	**11.391**	L3mbtl4	l(3)mbt-like 4 (Drosophila)
27033	A_52_P556462	0.017	0.192	**11.355**	Fancd2	Fanconi anemia, complementation group D2
51818	A_55_P2274508	0.016	0.180	**11.307**	4933416E03Rik	RIKEN cDNA 4933416E03 gene
7710	A_30_P01025134	0.016	0.178	**11.299**		
8254	A_30_P01025678	0.014	0.162	**11.288**		
52453	A_55_P2336078	0.014	0.159	**11.266**	4933404K13Rik	RIKEN cDNA 4933404K13 gene
50711	A_55_P2184889	0.017	0.191	**11.209**		
1465	A_30_P01018889	0.015	0.168	**11.150**		
1072	A_30_P01018496	0.049	0.547	**11.142**		
47585	A_55_P2143827	0.017	0.186	**11.047**		
34751	A_55_P2006747	0.016	0.175	**11.017**	Mfsd12	major facilitator superfamily domain containing 12
14071	A_30_P01031495	0.015	0.163	**11.009**		
9221	A_30_P01026645	0.017	0.186	**10.893**		
37348	A_55_P2031596	0.015	0.165	**10.870**		
15119	A_30_P01032543	0.013	0.142	**10.830**		
10243	A_30_P01027667	0.014	0.149	**10.797**		
52196	A_55_P2308363	0.013	0.145	**10.796**	AI851613	expressed sequence AI851613
40248	A_55_P2060552	0.014	0.149	**10.786**	Samt3	spermatogenesis associated multipass transmembrane protein 3
10859	A_30_P01028283	0.015	0.161	**10.744**		
391	A_30_P01017815	0.017	0.181	**10.705**		
17493	A_51_P173678	0.013	0.143	**10.700**	Slc10a6	solute carrier family 10 (sodium/bile acid cotransporter family), member 6
16096	A_30_P01033520	0.036	0.378	**10.458**		
16026	A_30_P01033450	0.015	0.156	**10.402**		
6886	A_30_P01024310	0.047	0.487	**10.396**		
35088	A_55_P2009817	0.016	0.168	**10.392**	Ssxb6	synovial sarcoma, X member B, breakpoint 6
44872	A_55_P2110778	0.015	0.157	**10.235**		
41989	A_55_P2078370	0.017	0.172	**10.204**	Rgs13	regulator of G-protein signaling 13
14007	A_30_P01031431	0.014	0.143	**10.180**		
43758	A_55_P2097967	0.053	0.540	**10.161**		
39826	A_55_P2056155	0.017	0.169	**10.056**	LOC102634959	leukocyte immunoglobulin-like receptor subfamily A member 5-like
40926	A_55_P2067538	0.016	0.156	**10.031**	Il1rl2	interleukin 1 receptor-like 2
4946	A_30_P01022370	0.014	0.145	**10.028**		
35407	A_55_P2012904	0.014	0.144	**9.963**		
5395	A_30_P01022819	0.013	0.133	**9.932**		
3322	A_30_P01020746	0.016	0.156	**9.901**		
34560	A_55_P2005165	0.014	0.140	**9.885**	Vmn1r8	vomeronasal 1 receptor 8

30273	A_55_P1967124	0.016	0.156	**9.845**	Ankef1	ankyrin repeat and EF-hand domain containing 1
22178	A_51_P459012	0.014	0.141	**9.778**	Zswim2	zinc finger SWIM-type containing 2
51088	A_55_P2204779	0.034	0.327	**9.746**	AU019990	expressed sequence AU019990
48469	A_55_P2155116	0.038	0.369	**9.744**	Gm5086	predicted gene 5086
47475	A_55_P2142605	0.024	0.237	**9.737**		
2138	A_30_P01019562	0.024	0.235	**9.718**		
13287	A_30_P01030711	0.015	0.145	**9.706**		
9611	A_30_P01027035	0.017	0.166	**9.693**		
27446	A_52_P604864	0.074	0.714	**9.661**	Il17rb	interleukin 17 receptor B
32961	A_55_P1990603	0.013	0.129	**9.636**	Olfr1448	olfactory receptor 1448
37174	A_55_P2029766	0.016	0.154	**9.623**	Gm13219	predicted gene 13219
45804	A_55_P2121886	0.045	0.428	**9.580**	Map3k9	mitogen-activated protein kinase kinase kinase 9
11285	A_30_P01028709	0.016	0.149	**9.526**		
37900	A_55_P2036994	0.014	0.137	**9.520**	Zic3	zinc finger protein of the cerebellum 3
11663	A_30_P01029087	0.019	0.179	**9.444**		
30696	A_55_P1970693	0.015	0.145	**9.440**		
6893	A_30_P01024317	0.030	0.282	**9.428**		
52828	A_55_P2373515	0.017	0.158	**9.416**		
25163	A_52_P322096	0.013	0.124	**9.377**	Patl2	protein associated with topoisomerase II homolog 2 (yeast)
14713	A_30_P01032137	0.024	0.224	**9.345**		
27969	A_52_P671676	0.015	0.143	**9.333**	Rasgrf1	RAS protein-specific guanine nucleotide-releasing factor 1
29395	A_55_P1959673	0.014	0.133	**9.322**	Gm4491	predicted gene 4491
39896	A_55_P2056846	0.017	0.159	**9.239**	Olfr525	olfactory receptor 525
26536	A_52_P496726	0.047	0.431	**9.144**	Rasd1	RAS, dexamethasone-induced 1
31939	A_55_P1981474	0.014	0.124	**9.098**		
22342	A_51_P468073	0.013	0.121	**9.096**	Ggt1	gamma-glutamyltransferase 1
28309	A_52_P87763	0.014	0.125	**9.009**	Olfr860	olfactory receptor 860
37454	A_55_P2032643	0.041	0.364	**8.939**	Ajap1	adherens junction associated protein 1
34155	A_55_P2001608	0.014	0.126	**8.903**	Klhl4	kelch-like 4
38465	A_55_P2042606	0.029	0.262	**8.893**	Pls1	plastin 1 (I-isoform)
35186	A_55_P2010877	0.016	0.143	**8.891**	Prrt2	proline-rich transmembrane protein 2
35944	A_55_P2018002	0.019	0.165	**8.828**	Abcg4	ATP-binding cassette, sub-family G (WHITE), member 4
44176	A_55_P2102763	0.013	0.117	**8.812**		
15111	A_30_P01032535	0.015	0.134	**8.802**		
51977	A_55_P2288047	0.014	0.127	**8.760**	Igkv6-15	immunoglobulin kappa variable 6-15
7283	A_30_P01024707	0.013	0.117	**8.758**		

7561	A_30_P01024985	0.048	0.415	**8.738**		
14475	A_30_P01031899	0.016	0.136	**8.710**		
32870	A_55_P1989842	0.015	0.130	**8.697**		
12923	A_30_P01030347	0.056	0.483	**8.666**		
45107	A_55_P2113391	0.030	0.257	**8.652**		
45457	A_55_P2117704	0.013	0.115	**8.634**	Pak7	p21 protein (Cdc42/Rac)-activated kinase 7
1822	A_30_P01019246	0.016	0.138	**8.602**		
45966	A_55_P2123907	0.019	0.160	**8.597**		
6522	A_30_P01023946	0.032	0.275	**8.595**		
14497	A_30_P01031921	0.015	0.127	**8.570**		
39720	A_55_P2055055	0.016	0.134	**8.564**	4930547M16Rik	RIKEN cDNA 4930547M16 gene
9047	A_30_P01026471	0.014	0.124	**8.552**		
2500	A_30_P01019924	0.016	0.134	**8.531**		
27056	A_52_P55876	0.016	0.137	**8.530**	Runx2	runt related transcription factor 2
50453	A_55_P2181301	0.018	0.150	**8.521**	Stpg2	sperm tail PG rich repeat containing 2
44304	A_55_P2104372	0.017	0.142	**8.500**	Gm12302	predicted gene 12302
44848	A_55_P2110532	0.015	0.131	**8.442**	4930578E11Rik	RIKEN cDNA 4930578E11 gene
17768	A_51_P191726	0.016	0.138	**8.409**	Efcab6	EF-hand calcium binding domain 6
31754	A_55_P1979943	0.014	0.114	**8.363**		
13945	A_30_P01031369	0.015	0.124	**8.345**		
14774	A_30_P01032198	0.016	0.132	**8.329**		
37490	A_55_P2032905	0.019	0.154	**8.306**		
55071	A_66_P128345	0.031	0.259	**8.283**	Slc5a11	solute carrier family 5 (sodium/glucose cotransporter), member 11
55313	A_66_P133148	0.014	0.119	**8.235**	Aoah	acyloxyacyl hydrolase
31381	A_55_P1976634	0.017	0.134	**7.948**	Olfr657	olfactory receptor 657
13984	A_30_P01031408	0.015	0.119	**7.932**		
44983	A_55_P2112142	0.021	0.163	**7.912**	Tmbim7	transmembrane BAX inhibitor motif containing 7
3558	A_30_P01020982	0.017	0.133	**7.893**		
8788	A_30_P01026212	0.016	0.124	**7.887**		
29729	A_55_P1962445	0.018	0.138	**7.867**	Skint11	selection and upkeep of intraepithelial T cells 11
19968	A_51_P325198	0.017	0.134	**7.848**	3110009E18Rik	RIKEN cDNA 3110009E18 gene
28487	A_55_P1952411	0.015	0.119	**7.841**		
11380	A_30_P01028804	0.062	0.488	**7.830**		
21334	A_51_P406306	0.016	0.121	**7.804**	Hecw2	HECT, C2 and WW domain containing E3 ubiquitin protein ligase 2
31179	A_55_P1974760	0.017	0.132	**7.799**	Duox2	dual oxidase 2
42096	A_55_P2079636	0.017	0.128	**7.764**	Scgb3a2	secretoglobin, family 3A, member 2

7627	A_30_P01025051	0.014	0.108	**7.758**		
26586	A_52_P502838	0.047	0.365	**7.739**	Mgat5	mannoside acetylglucosaminyltransferase 5
35509	A_55_P2013893	0.015	0.114	**7.731**	Cntn4	contactin 4
24671	A_52_P258796	0.016	0.120	**7.723**		
14998	A_30_P01032422	0.016	0.127	**7.697**		
3734	A_30_P01021158	0.027	0.206	**7.601**		
1642	A_30_P01019066	0.014	0.105	**7.598**		
985	A_30_P01018409	0.017	0.132	**7.585**		
40853	A_55_P2066737	0.022	0.169	**7.582**		
43935	A_55_P2099991	0.018	0.133	**7.558**	Gm10696	predicted gene 10696
24726	A_52_P265965	0.014	0.109	**7.545**	Trim36	tripartite motif-containing 36
53093	A_55_P2402781	0.031	0.229	**7.471**	B230216N24Rik	RIKEN cDNA B230216N24 gene
47154	A_55_P2138600	0.047	0.349	**7.431**	Tmprss3	transmembrane protease, serine 3
33336	A_55_P1994068	0.019	0.137	**7.384**	Rbp1	retinol binding protein 1, cellular
8588	A_30_P01026012	0.076	0.557	**7.358**		
40577	A_55_P2064014	0.013	0.099	**7.331**	Fam122c	family with sequence similarity 122, member C
28465	A_55_P1952212	0.015	0.106	**7.228**		
12311	A_30_P01029735	0.017	0.125	**7.206**		
46758	A_55_P2133617	0.056	0.399	**7.169**	1700016A09Rik	RIKEN cDNA 1700016A09 gene
10782	A_30_P01028206	0.014	0.100	**7.123**		
51535	A_55_P2245467	0.015	0.106	**7.116**	4930452A19Rik	RIKEN cDNA 4930452A19 gene
21257	A_51_P401386	0.015	0.104	**7.095**		
15380	A_30_P01032804	0.014	0.101	**7.013**		
32674	A_55_P1988063	0.024	0.165	**6.971**		
29646	A_55_P1961756	0.014	0.098	**6.961**	Vmn2r52	vomeronasal 2, receptor 52
3967	A_30_P01021391	0.014	0.096	**6.949**		
3640	A_30_P01021064	0.013	0.090	**6.934**		
33044	A_55_P1991451	0.016	0.113	**6.909**		
41820	A_55_P2076605	0.023	0.157	**6.899**	Plekho2	pleckstrin homology domain containing, family O member 2
3770	A_30_P01021194	0.014	0.100	**6.892**		
32838	A_55_P1989599	0.017	0.120	**6.864**	Spdyb	speedy homolog B (Xenopus laevis)
30136	A_55_P1965906	0.017	0.113	**6.809**	Gm9999	predicted gene 9999
51741	A_55_P2264762	0.022	0.146	**6.736**	4933407K13Rik	RIKEN cDNA 4933407K13 gene
14036	A_30_P01031460	0.014	0.094	**6.710**		
24179	A_52_P203316	0.016	0.104	**6.689**	Kcnf1	potassium voltage-gated channel, subfamily F, member 1
19864	A_51_P318510	0.023	0.154	**6.676**	Ptprn	protein tyrosine phosphatase, receptor type, N

9565	A_30_P01026989	0.014	0.096	**6.668**		
52804	A_55_P2371242	0.016	0.106	**6.652**	C80171	expressed sequence C80171
26837	A_52_P533030	0.022	0.143	**6.587**	Olfr237-ps1	olfactory receptor 237, pseudogene 1
48473	A_55_P2155161	0.013	0.086	**6.551**		
20390	A_51_P349413	0.013	0.087	**6.532**	Arl5c	ADP-ribosylation factor-like 5C
26383	A_52_P480266	0.066	0.429	**6.510**	Atat1	alpha tubulin acetyltransferase 1
24936	A_52_P294490	0.013	0.085	**6.502**	Odf1	outer dense fiber of sperm tails 1
54052	A_66_P107483	0.018	0.114	**6.495**	Treml2	triggering receptor expressed on myeloid cells-like 2
42951	A_55_P2088945	0.034	0.221	**6.491**	Vstm2b	V-set and transmembrane domain containing 2B
34780	A_55_P2007010	0.052	0.332	**6.438**	Frmd4b	FERM domain containing 4B
8036	A_30_P01025460	0.016	0.104	**6.436**		
23361	A_52_P104658	0.015	0.093	**6.429**	Krt6a	keratin 6A
45513	A_55_P2118412	0.014	0.093	**6.413**		
43298	A_55_P2092958	0.035	0.226	**6.408**	Traf2	TNF receptor-associated factor 2
38180	A_55_P2039681	0.015	0.097	**6.359**	Gpr35	G protein-coupled receptor 35
9757	A_30_P01027181	0.039	0.245	**6.348**		
25355	A_52_P348201	0.024	0.153	**6.320**	Wee2	WEE1 homolog 2 (S. pombe)
22954	A_51_P501990	0.013	0.083	**6.312**	Smok4a	sperm motility kinase 4A
9703	A_30_P01027127	0.050	0.316	**6.297**		
40677	A_55_P2064984	0.032	0.202	**6.270**	Cenpi	centromere protein I
5681	A_30_P01023105	0.014	0.088	**6.259**		
18675	A_51_P245038	0.013	0.082	**6.250**	1700011A15Rik	RIKEN cDNA 1700011A15 gene
232	A_30_P01017656	0.049	0.305	**6.237**		
12110	A_30_P01029534	0.015	0.093	**6.199**		
8591	A_30_P01026015	0.016	0.099	**6.178**		
52803	A_55_P2371237	0.053	0.330	**6.163**	Kcnip3	Kv channel interacting protein 3, calsenilin
11084	A_30_P01028508	0.039	0.238	**6.148**		
3024	A_30_P01020448	0.016	0.099	**6.145**		
25975	A_52_P425839	0.074	0.451	**6.116**	Retnlg	resistin like gamma
19209	A_51_P277756	0.028	0.174	**6.114**	Tas1r2	taste receptor, type 1, member 2
54972	A_66_P126110	0.016	0.098	**6.104**	Pcdh11x	protocadherin 11 X-linked
52535	A_55_P2344608	0.049	0.297	**6.100**	D7Wsu130e	DNA segment, Chr 7, Wayne State University 130, expressed
28250	A_52_P8251	0.015	0.089	**6.047**		
51119	A_55_P2207215	0.015	0.090	**6.032**	Gm10419	predicted gene 10419
20115	A_51_P334155	0.043	0.256	**6.014**	4930579F01Rik	RIKEN cDNA 4930579F01 gene
52801	A_55_P2370931	0.017	0.103	**5.998**	AU017674	expressed sequence AU017674

45275	A_55_P2115355	0.015	0.089	**5.984**	LOC102638714	uncharacterized LOC102638714
10135	A_30_P01027559	0.014	0.082	**5.940**		
20772	A_51_P373669	0.016	0.096	**5.923**	Mrvi1	MRV integration site 1
21329	A_51_P406157	0.017	0.103	**5.920**	Calcb	calcitonin-related polypeptide, beta
10587	A_30_P01028011	0.014	0.082	**5.911**		
39530	A_55_P2053127	0.017	0.101	**5.905**	Foxg1	forkhead box G1
51975	A_55_P2287910	0.036	0.212	**5.881**	Rragd	Ras-related GTP binding D
46213	A_55_P2127015	0.016	0.095	**5.871**	Olfr720	olfactory receptor 720
29244	A_55_P1958479	0.045	0.263	**5.867**	4930555G01Rik	RIKEN cDNA 4930555G01 gene
24956	A_52_P297176	0.014	0.082	**5.866**	E230001N04Rik	RIKEN cDNA E230001N04 gene
24003	A_52_P180917	0.014	0.081	**5.856**	Kcnq2	potassium voltage-gated channel, subfamily Q, member 2
3701	A_30_P01021125	0.024	0.140	**5.852**		
40745	A_55_P2065577	0.016	0.092	**5.844**	6430503K07Rik	RIKEN cDNA 6430503K07 gene
23518	A_52_P1190151	0.018	0.103	**5.812**		
23854	A_52_P161488	0.013	0.076	**5.788**	Clec4e	C-type lectin domain family 4, member e
41212	A_55_P2070366	0.034	0.196	**5.785**	Nlrp1a	NLR family, pyrin domain containing 1A
29324	A_55_P1959146	0.015	0.084	**5.765**	Gm13119	predicted gene 13119
25104	A_52_P314129	0.017	0.099	**5.761**	Pkia	protein kinase inhibitor, alpha
8594	A_30_P01026018	0.034	0.196	**5.759**		
41281	A_55_P2071112	0.029	0.170	**5.757**	Vmn2r23	vomeronasal 2, receptor 23
14443	A_30_P01031867	0.014	0.082	**5.738**		
48327	A_55_P2153116	0.084	0.480	**5.695**	Tsix	X (inactive)-specific transcript, opposite strand
55251	A_66_P131896	0.017	0.096	**5.682**	Olfr117	olfactory receptor 117
26845	A_52_P533270	0.017	0.095	**5.681**	2310005G13Rik	RIKEN cDNA 2310005G13 gene
21974	A_51_P444645	0.014	0.081	**5.649**	Lect1	leukocyte cell derived chemotaxin 1
43697	A_55_P2097333	0.027	0.153	**5.622**	Gm5334	tetraspanin 7 pseudogene
25480	A_52_P362917	0.062	0.349	**5.615**	Pfkfb3	6-phosphofructo-2-kinase/fructose-2,6-biphosphatase 3
6745	A_30_P01024169	0.020	0.112	**5.594**		
21667	A_51_P426195	0.049	0.274	**5.580**	Nppb	natriuretic peptide type B
18040	A_51_P208160	0.016	0.087	**5.578**	Slc7a13	solute carrier family 7, (cationic amino acid transporter, y+ system) member 13
4501	A_30_P01021925	0.013	0.074	**5.576**		
22470	A_51_P475704	0.017	0.095	**5.565**	Pcdh9	protocadherin 9
45570	A_55_P2118996	0.015	0.083	**5.561**	Foxe1	forkhead box E1
24066	A_52_P188746	0.017	0.094	**5.540**	Tmem67	transmembrane protein 67
32370	A_55_P1985490	0.016	0.086	**5.532**		
55004	A_66_P126586	0.035	0.196	**5.530**		

28499	A_55_P1952517	0.017	0.094	**5.521**	Sult2a1	sulfotransferase family 2A, dehydroepiandrosterone (DHEA)-preferring, member 1
43812	A_55_P2098609	0.015	0.084	**5.515**	Spag16	sperm associated antigen 16
14226	A_30_P01031650	0.017	0.093	**5.515**		
7499	A_30_P01024923	0.016	0.086	**5.510**		
28599	A_55_P1953316	0.034	0.187	**5.504**	Olfr1141	olfactory receptor 1141
33629	A_55_P1997081	0.017	0.092	**5.493**	Faxc	failed axon connections homolog (Drosophila)
5061	A_30_P01022485	0.015	0.079	**5.428**		
42884	A_55_P2088237	0.015	0.079	**5.426**	Cyp27b1	cytochrome P450, family 27, subfamily b, polypeptide 1
50257	A_55_P2178653	0.058	0.312	**5.424**		
48696	A_55_P2157794	0.013	0.073	**5.420**	Nyap2	neuronal tyrosine-phophorylated phosphoinositide 3-kinase adaptor 2
31190	A_55_P1974869	0.016	0.085	**5.405**	Hormad1	HORMA domain containing 1
29406	A_55_P1959788	0.014	0.074	**5.385**	Gm10697	predicted gene 10697
28156	A_52_P744179	0.031	0.164	**5.378**	Xirp2	xin actin-binding repeat containing 2
2620	A_30_P01020044	0.015	0.080	**5.340**		
50364	A_55_P2180173	0.015	0.079	**5.282**	Gm4201	predicted gene 4201
42559	A_55_P2084691	0.025	0.133	**5.271**		
25521	A_52_P368306	0.066	0.348	**5.259**	Tmem100	transmembrane protein 100
1048	A_30_P01018472	0.030	0.156	**5.249**		
11435	A_30_P01028859	0.025	0.132	**5.220**		
17004	A_51_P144543	0.017	0.088	**5.208**	1700063H04Rik	RIKEN cDNA 1700063H04 gene
33982	A_55_P2000052	0.017	0.091	**5.206**		
38537	A_55_P2043277	0.017	0.087	**5.186**	Siglec5	sialic acid binding Ig-like lectin 5
8337	A_30_P01025761	0.016	0.081	**5.171**		
14420	A_30_P01031844	0.015	0.077	**5.164**		
33372	A_55_P1994365	0.013	0.068	**5.162**	Gm5916	predicted gene 5916
1916	A_30_P01019340	0.016	0.084	**5.157**		
43260	A_55_P2092441	0.016	0.082	**5.151**		
37080	A_55_P2028883	0.043	0.219	**5.123**	Rnft2	ring finger protein, transmembrane 2
15297	A_30_P01032721	0.021	0.107	**5.115**		
22445	A_51_P474053	0.019	0.099	**5.095**	Erv3	endogenous retroviral sequence 3
1278	A_30_P01018702	0.014	0.070	**5.095**		
51850	A_55_P2276761	0.056	0.284	**5.084**	4930474N09Rik	RIKEN cDNA 4930474N09 gene
34221	A_55_P2002275	0.017	0.088	**5.051**	H2-T18	histocompatibility 2, T region locus 18
37055	A_55_P2028680	0.018	0.089	**5.049**	Spaca3	sperm acrosome associated 3
539	A_30_P01017963	0.014	0.068	**5.047**		
39711	A_55_P2054967	0.016	0.079	**5.042**	Lrrc49	leucine rich repeat containing 49

25145	A_52_P319541	0.015	0.075	**5.033**	Med12l	mediator complex subunit 12-like
41465	A_55_P2072995	0.013	0.067	**5.031**		
24227	A_52_P208416	0.017	0.084	**5.022**	Olfr1431	olfactory receptor 1431
50343	A_55_P2179884	0.016	0.080	**5.020**		
53685	A_66_P100113	0.016	0.079	**5.012**	Dynlt1a	dynein light chain Tctex-type 1A
19742	A_51_P310821	0.027	0.136	**5.005**	Hoxa5	homeobox A5

Microarray data_Down292

Narabi_55681	ProbeName	LC(normalized)	LV(normalized)	Fold change	GeneSymbol	GeneName
2413	A_30_P01019837	0.801	0.072	**0.090**		
32786	A_55_P1989149	0.712	0.070	**0.098**	Olfr394	olfactory receptor 394
21104	A_51_P392385	0.797	0.080	**0.101**	Smcp	sperm mitochondria-associated cysteine-rich protein
3033	A_30_P01020457	0.662	0.067	**0.101**		
6097	A_30_P01023521	0.661	0.068	**0.102**		
11170	A_30_P01028594	0.742	0.082	**0.111**		
25139	A_52_P318673	728.464	88.015	**0.121**	Saa1	serum amyloid A 1
41258	A_55_P2070869	29.224	3.539	**0.121**	Lcn2	lipocalin 2
51896	A_55_P2279927	0.535	0.065	**0.122**	5930430L01Rik	RIKEN cDNA 5930430L01 gene
33418	A_55_P1994807	760.859	93.361	**0.123**	Saa2	serum amyloid A 2
25415	A_52_P354965	0.581	0.072	**0.125**	Ric3	resistance to inhibitors of cholinesterase 3 homolog (C. elegans)
53220	A_55_P2414909	0.684	0.091	**0.133**	4933411B09Rik	RIKEN cDNA 4933411B09 gene
53154	A_55_P2408588	1.105	0.154	**0.139**	Arntl	aryl hydrocarbon receptor nuclear translocator-like
31168	A_55_P1974660	0.619	0.088	**0.143**		
12666	A_30_P01030090	0.469	0.067	**0.143**		
52898	A_55_P2381821	0.528	0.077	**0.146**	6430706H07Rik	RIKEN cDNA 6430706H07 gene
47201	A_55_P2139207	0.543	0.082	**0.152**	Ccdc142	coiled-coil domain containing 142
20670	A_51_P367423	0.651	0.100	**0.154**	Cldnd2	claudin domain containing 2
20410	A_51_P350817	0.563	0.089	**0.158**	Cnn1	calponin 1
33671	A_55_P1997424	0.695	0.112	**0.161**	Cwc22	CWC22 spliceosome-associated protein homolog (S. cerevisiae)
52314	A_55_P2322555	0.453	0.074	**0.164**	5930433N17Rik	RIKEN cDNA 5930433N17 gene
24113	A_52_P194851	0.680	0.115	**0.169**	2410089E03Rik	RIKEN cDNA 2410089E03 gene
37291	A_55_P2030938	0.354	0.061	**0.172**	Trim59	tripartite motif-containing 59
38899	A_55_P2046563	0.440	0.077	**0.174**	Cym	chymosin
43636	A_55_P2096630	0.854	0.149	**0.175**	Ssxb9	synovial sarcoma, X member B, breakpoint 9

51124	A_55_P2207498	0.627	0.114	**0.182**	Image:619641	uncharacterized LOC541456
54178	A_66_P109978	0.588	0.111	**0.189**	Bnip2	BCL2/adenovirus E1B interacting protein 2
44530	A_55_P2106901	0.754	0.145	**0.192**	Nkain4	Na+/K+ transporting ATPase interacting 4
19748	A_51_P311362	1.109	0.219	**0.198**	4930449E01Rik	RIKEN cDNA 4930449E01 gene
23651	A_52_P13730	2.721	0.539	**0.198**	Cbx2	chromobox 2
18542	A_51_P237106	0.558	0.114	**0.204**	Olfr1211	olfactory receptor 1211
51075	A_55_P2202558	0.750	0.156	**0.208**		
8749	A_30_P01026173	0.385	0.081	**0.210**		
33209	A_55_P1993001	0.585	0.126	**0.215**		
2425	A_30_P01019849	0.382	0.084	**0.220**		
55226	A_66_P131137	0.372	0.083	**0.223**		
13936	A_30_P01031360	0.362	0.082	**0.228**		
29892	A_55_P1963807	0.300	0.071	**0.236**	Actg2	actin, gamma 2, smooth muscle, enteric
13327	A_30_P01030751	0.380	0.091	**0.240**		
13743	A_30_P01031167	0.506	0.122	**0.241**		
25843	A_52_P408736	0.446	0.108	**0.241**	Slc16a7	solute carrier family 16 (monocarboxylic acid transporters), member 7
17217	A_51_P156955	0.391	0.095	**0.242**	Cfd	complement factor D (adipsin)
25911	A_52_P418644	1.488	0.362	**0.243**	Plp2	proteolipid protein 2
32058	A_55_P1982532	0.500	0.123	**0.245**		
46696	A_55_P2132932	0.675	0.166	**0.246**	Mageb1	melanoma antigen, family B, 1
19407	A_51_P289828	0.559	0.139	**0.248**	Ccdc138	coiled-coil domain containing 138
44493	A_55_P2106514	0.525	0.134	**0.254**	Scn2a1	sodium channel, voltage-gated, type II, alpha 1
22743	A_51_P490023	67.175	17.094	**0.254**	Tubb2a	tubulin, beta 2A class IIA
37311	A_55_P2031167	25.425	6.511	**0.256**	Efna1	ephrin A1
22133	A_51_P454993	0.267	0.069	**0.258**	Tmcc2	transmembrane and coiled-coil domains 2
27460	A_52_P606826	0.684	0.176	**0.258**	Pigh	phosphatidylinositol glycan anchor biosynthesis, class H
50016	A_55_P2175356	0.450	0.117	**0.260**	Uros	uroporphyrinogen III synthase
7962	A_30_P01025386	0.301	0.079	**0.263**		
13161	A_30_P01030585	1.023	0.276	**0.270**		
52792	A_55_P2370337	0.282	0.078	**0.278**	Cdk13	cyclin-dependent kinase 13
208	A_30_P01017632	0.371	0.103	**0.279**		
45547	A_55_P2118744	0.325	0.092	**0.281**	Sez6l	seizure related 6 homolog like
20450	A_51_P353232	0.331	0.094	**0.282**	Tnnc2	troponin C2, fast
23694	A_52_P141488	0.950	0.270	**0.284**	Grk5	G protein-coupled receptor kinase 5
42287	A_55_P2081780	0.883	0.252	**0.285**	Asb7	ankyrin repeat and SOCS box-containing 7
9304	A_30_P01026728	0.396	0.114	**0.288**		

19332	A_51_P285206	0.654	0.189	**0.288**	Cd3d	CD3 antigen, delta polypeptide
33613	A_55_P1996946	0.779	0.228	**0.293**	Cdc20	cell division cycle 20
36294	A_55_P2021423	0.280	0.083	**0.296**	Morn3	MORN repeat containing 3
12806	A_30_P01030230	0.385	0.114	**0.297**		
44637	A_55_P2108151	3.236	0.975	**0.301**	Hbb-b1	hemoglobin, beta adult major chain
19040	A_51_P267354	6.569	1.984	**0.302**	Lrfn3	leucine rich repeat and fibronectin type III domain containing 3
20677	A_51_P368009	1.442	0.437	**0.303**	E2f2	E2F transcription factor 2
46153	A_55_P2126368	0.218	0.066	**0.304**	Atad5	ATPase family, AAA domain containing 5
29124	A_55_P1957624	16.789	5.180	**0.309**	Insig2	insulin induced gene 2
45343	A_55_P2116310	0.294	0.091	**0.310**	Trim45	tripartite motif-containing 45
28053	A_52_P681391	14.757	4.597	**0.312**	G0s2	G0/G1 switch gene 2
15653	A_30_P01033077	0.916	0.285	**0.312**		
20682	A_51_P368394	0.235	0.074	**0.313**	Dnajb8	DnaJ (Hsp40) homolog, subfamily B, member 8
44960	A_55_P2111855	22.834	7.145	**0.313**	Gale	galactose-4-epimerase, UDP
21893	A_51_P439452	52.148	16.349	**0.314**	Insig2	insulin induced gene 2
46137	A_55_P2126072	0.328	0.103	**0.315**		
44776	A_55_P2109717	0.252	0.080	**0.317**	Kif20b	kinesin family member 20B
29167	A_55_P1957918	0.947	0.301	**0.318**	Asap2	ArfGAP with SH3 domain, ankyrin repeat and PH domain 2
42038	A_55_P2078955	7.657	2.433	**0.318**	Aqp8	aquaporin 8
29707	A_55_P1962303	62.825	20.201	**0.322**	Hba-a1	hemoglobin alpha, adult chain 1
32341	A_55_P1985286	0.368	0.119	**0.322**	Slc32a1	solute carrier family 32 (GABA vesicular transporter), member 1
23788	A_52_P15377	0.694	0.227	**0.328**	Wnt9b	wingless-type MMTV integration site 9B
19688	A_51_P307864	0.628	0.207	**0.330**	1700019N19Rik	RIKEN cDNA 1700019N19 gene
19955	A_51_P324633	44.363	14.721	**0.332**	Elovl3	elongation of very long chain fatty acids (FEN1/Elo2, SUR4/Elo3, yeast)-like 3
10223	A_30_P01027647	0.408	0.135	**0.332**		
47980	A_55_P2148684	0.292	0.097	**0.334**	Best1	bestrophin 1
10930	A_30_P01028354	0.669	0.226	**0.337**		
19596	A_51_P302139	0.367	0.124	**0.338**	Mastl	microtubule associated serine/threonine kinase-like
27964	A_52_P671062	0.296	0.100	**0.339**	Uba6	ubiquitin-like modifier activating enzyme 6
41932	A_55_P2077783	2.522	0.854	**0.339**	Tubb2a-ps2	tubulin, beta 2a, pseudogene 2
41809	A_55_P2076538	1.010	0.343	**0.339**	Whamm	WAS protein homolog associated with actin, golgi membranes and microtubules
14694	A_30_P01032118	0.408	0.140	**0.342**		
16191	A_30_P01033615	0.492	0.170	**0.345**		
53448	A_65_P01319	1.270	0.439	**0.346**	Pde4b	phosphodiesterase 4B, cAMP specific
20301	A_51_P344552	1.338	0.466	**0.348**	Irs4	insulin receptor substrate 4
3861	A_30_P01021285	0.317	0.111	**0.349**		

24143	A_52_P198239	0.533	0.186	**0.349**	Ube2u	ubiquitin-conjugating enzyme E2U (putative)
38067	A_55_P2038540	7.573	2.647	**0.349**	Hbb-b2	hemoglobin, beta adult minor chain
40500	A_55_P2063146	0.299	0.105	**0.350**		
16712	A_51_P126626	0.337	0.118	**0.350**	Zfp503	zinc finger protein 503
55673	A_66_P140856	0.232	0.082	**0.352**	LOC102634502	uncharacterized LOC102634502
52271	A_55_P2318584	128.176	45.184	**0.353**	Aqp8	aquaporin 8
23921	A_52_P169082	0.513	0.181	**0.354**	Dbil5	diazepam binding inhibitor-like 5
23882	A_52_P164136	6.870	2.438	**0.355**	Arrdc3	arrestin domain containing 3
611	A_30_P01018035	0.207	0.074	**0.357**		
34649	A_55_P2005883	0.200	0.072	**0.360**	Foxr1	forkhead box R1
31065	A_55_P1973809	28.682	10.328	**0.360**	Hbb-bt	hemoglobin, beta adult t chain
29706	A_55_P1962299	260.339	94.654	**0.364**	Hba-a2	hemoglobin alpha, adult chain 2
34322	A_55_P2003092	0.169	0.062	**0.367**	Sbpl	spermine binding protein-like
42971	A_55_P2089209	0.331	0.122	**0.369**		
9017	A_30_P01026441	0.202	0.075	**0.369**		
35095	A_55_P2009882	0.559	0.206	**0.369**	Ccbe1	collagen and calcium binding EGF domains 1
35693	A_55_P2015782	0.721	0.268	**0.372**	Bcas1	breast carcinoma amplified sequence 1
46225	A_55_P2127194	0.293	0.109	**0.372**		
51882	A_55_P2279140	5.341	2.006	**0.376**	F830014O18Rik	RIKEN cDNA F830014O18 gene
9537	A_30_P01026961	0.171	0.064	**0.376**		
19952	A_51_P324529	0.191	0.072	**0.378**	Calcoco2	calcium binding and coiled-coil domain 2
45411	A_55_P2117222	0.321	0.122	**0.380**	Lhfpl5	lipoma HMGIC fusion partner-like 5
22652	A_51_P485421	0.292	0.111	**0.380**		
5901	A_30_P01023325	0.271	0.104	**0.383**		
28320	A_52_P883557	1.918	0.736	**0.384**	Slc30a10	solute carrier family 30, member 10
20945	A_51_P383774	0.730	0.281	**0.385**	Gngt1	guanine nucleotide binding protein (G protein), gamma transducing activity polypeptide 1
32174	A_55_P1983768	0.177	0.068	**0.386**	Birc5	baculoviral IAP repeat-containing 5
52789	A_55_P2370210	0.575	0.222	**0.387**	1700025K04Rik	RIKEN cDNA 1700025K04 gene
24982	A_52_P300445	0.199	0.077	**0.387**	Atp4a	ATPase, H+/K+ exchanging, gastric, alpha polypeptide
19135	A_51_P272735	0.195	0.075	**0.387**	Gm7968	predicted gene 7968
19037	A_51_P267278	0.321	0.124	**0.387**	Slc15a2	solute carrier family 15 (H+/peptide transporter), member 2
21821	A_51_P435363	0.614	0.240	**0.390**	Lipe	lipase, hormone sensitive
44554	A_55_P2107182	79.921	31.270	**0.391**	Gm6484	predicted gene 6484
32677	A_55_P1988083	0.341	0.134	**0.393**	Prc1	protein regulator of cytokinesis 1
18589	A_51_P240269	0.330	0.130	**0.395**	Fam199x	family with sequence similarity 199, X-linked

7421	A_30_P01024845	0.373	0.147	**0.395**		
40413	A_55_P2062324	0.278	0.110	**0.396**	LOC102634570	Y-linked testis-specific protein 1-like
31727	A_55_P1979674	0.485	0.193	**0.399**	Ikzf4	IKAROS family zinc finger 4
25251	A_52_P33382	0.179	0.072	**0.401**		
2050	A_30_P01019474	0.562	0.226	**0.403**		
20440	A_51_P352594	1.445	0.582	**0.403**	St5	suppression of tumorigenicity 5
33339	A_55_P1994112	0.202	0.081	**0.403**	Sema5b	sema domain, seven thrombospondin repeats (type 1 and type 1-like), transmembrane domain (TM) and short cytoplasmic domain, (semaphorin) 5B
26446	A_52_P48681	0.963	0.388	**0.403**	Cldn1	claudin 1
1058	A_30_P01018482	0.206	0.083	**0.404**		
33362	A_55_P1994290	0.148	0.060	**0.406**	Gm10791	predicted gene 10791
26963	A_52_P549190	0.158	0.064	**0.408**	Cldn8	claudin 8
48542	A_55_P2156062	0.187	0.076	**0.408**	LOC102632642	galectin-related protein-like
33030	A_55_P1991295	0.739	0.302	**0.409**	BC029214	cDNA sequence BC029214
23220	A_51_P517051	0.223	0.091	**0.410**	Gatsl3	GATS protein-like 3
16432	A_51_P110371	0.816	0.338	**0.414**	Wwox	WW domain-containing oxidoreductase
21903	A_51_P440047	2.508	1.039	**0.414**	Lgalsl	lectin, galactoside binding-like
27786	A_52_P648688	0.643	0.267	**0.414**	Zc3h12d	zinc finger CCCH type containing 12D
39383	A_55_P2051656	0.234	0.097	**0.416**	Shank2	SH3/ankyrin domain gene 2
50903	A_55_P2187918	0.286	0.120	**0.418**	Cep55	centrosomal protein 55
21912	A_51_P440743	6.092	2.552	**0.419**	Celsr1	cadherin, EGF LAG seven-pass G-type receptor 1 (flamingo homolog, Drosophila)
21745	A_51_P430900	7.143	3.004	**0.421**	Dusp1	dual specificity phosphatase 1
40004	A_55_P2057984	0.550	0.232	**0.421**	Tbc1d19	TBC1 domain family, member 19
27414	A_52_P600535	0.336	0.141	**0.421**	Zfp39	zinc finger protein 39
32638	A_55_P1987811	0.470	0.198	**0.422**	LOC102638557	keratin-associated protein 20-2-like
16970	A_51_P142923	1.733	0.732	**0.422**	Chka	choline kinase alpha
53179	A_55_P2410626	0.800	0.339	**0.423**	A430061O12Rik	RIKEN cDNA A430061O12 gene
29525	A_55_P1960735	13.734	5.815	**0.423**	Gdf15	growth differentiation factor 15
50054	A_55_P2175905	0.447	0.191	**0.426**	Daw1	dynein assembly factor with WDR repeat domains 1
48563	A_55_P2156304	0.478	0.204	**0.427**	Kcnj16	potassium inwardly-rectifying channel, subfamily J, member 16
40440	A_55_P2062589	0.755	0.324	**0.428**	Mtnr1a	melatonin receptor 1A
26231	A_52_P462257	0.363	0.156	**0.429**		
39278	A_55_P2050495	0.163	0.070	**0.429**	Slc29a3	solute carrier family 29 (nucleoside transporters), member 3
53667	A_65_P20104	0.140	0.060	**0.430**	Mical2	microtubule associated monooxygenase, calponin and LIM domain containing 2
51390	A_55_P2233462	0.638	0.274	**0.430**	2700022O18Rik	RIKEN cDNA 2700022O18 gene
53321	A_55_P2424060	0.381	0.164	**0.430**	Nrg3os	neuregulin 3, opposite strand

34358	A_55_P2003438	0.296	0.127	**0.431**	Nr6a1	nuclear receptor subfamily 6, group A, member 1
16557	A_51_P116932	0.446	0.192	**0.431**	Lad1	ladinin
55508	A_66_P137285	0.266	0.115	**0.433**	A730045E13Rik	RIKEN cDNA A730045E13 gene
32495	A_55_P1986596	0.449	0.195	**0.433**	Cacna1h	calcium channel, voltage-dependent, T type, alpha 1H subunit
41763	A_55_P2075977	0.346	0.150	**0.434**		
47596	A_55_P2143923	3.390	1.476	**0.435**	Slc13a2	solute carrier family 13 (sodium-dependent dicarboxylate transporter), member 2
20184	A_51_P338262	0.301	0.131	**0.436**	Tnnt2	troponin T2, cardiac
38761	A_55_P2045380	0.606	0.264	**0.436**		
19957	A_51_P324651	0.451	0.197	**0.436**	Lphn1	latrophilin 1
28342	A_52_P89335	3.552	1.556	**0.438**	Tmie	transmembrane inner ear
25839	A_52_P408438	0.411	0.180	**0.439**	Arpp19	cAMP-regulated phosphoprotein 19
25627	A_52_P381303	0.190	0.083	**0.439**	Gins2	GINS complex subunit 2 (Psf2 homolog)
45635	A_55_P2119917	0.351	0.154	**0.439**	Ikzf4	IKAROS family zinc finger 4
51086	A_55_P2204491	0.892	0.393	**0.440**		
38134	A_55_P2039279	0.560	0.246	**0.440**	Kcnh3	potassium voltage-gated channel, subfamily H (eag-related), member 3
4586	A_30_P01022010	0.207	0.091	**0.440**		
41695	A_55_P2075263	2.764	1.219	**0.441**	Acnat2	acyl-coenzyme A amino acid N-acyltransferase 2
42244	A_55_P2081323	0.217	0.096	**0.442**	Gnas	GNAS (guanine nucleotide binding protein, alpha stimulating) complex locus
25948	A_52_P423247	0.344	0.152	**0.442**	Pde4b	phosphodiesterase 4B, cAMP specific
27502	A_52_P613643	0.336	0.149	**0.442**	Zfp3	zinc finger protein 3
34192	A_55_P2002033	0.193	0.085	**0.442**	Wdr89	WD repeat domain 89
47471	A_55_P2142575	0.248	0.110	**0.443**	Tdrd5	tudor domain containing 5
22387	A_51_P470715	4.423	1.960	**0.443**	Cish	cytokine inducible SH2-containing protein
41090	A_55_P2069251	0.142	0.063	**0.445**	Prr18	proline rich 18
18876	A_51_P257675	0.659	0.294	**0.447**	Tspyl4	TSPY-like 4
43389	A_55_P2094016	0.162	0.072	**0.448**	Slc17a4	solute carrier family 17 (sodium phosphate), member 4
2353	A_30_P01019777	0.141	0.063	**0.448**		
15444	A_30_P01032868	0.538	0.242	**0.449**		
40209	A_55_P2060188	0.712	0.320	**0.449**	Hira	histone cell cycle regulation defective homolog A (S. cerevisiae)
43781	A_55_P2098275	0.351	0.158	**0.450**		
39645	A_55_P2054342	2.332	1.049	**0.450**	2810408I11Rik	RIKEN cDNA 2810408I11 gene
11973	A_30_P01029397	1.129	0.508	**0.450**		
24763	A_52_P2710	13.123	5.921	**0.451**	Cml5	camello-like 5
18806	A_51_P252859	2.418	1.097	**0.454**	Cyr61	cysteine rich protein 61
18318	A_51_P224164	0.569	0.258	**0.454**	Slc26a4	solute carrier family 26, member 4
47465	A_55_P2142453	0.288	0.131	**0.455**	Gm3853	predicted gene 3853

19755	A_51_P311958	2.304	1.048	**0.455**	Orm3	orosomucoid 3
50732	A_55_P2185178	0.206	0.094	**0.455**	Tmem28	transmembrane protein 28
45747	A_55_P2121215	0.180	0.082	**0.455**	Clcc1	chloride channel CLIC-like 1
22442	A_51_P473940	0.552	0.252	**0.456**	Slc22a15	solute carrier family 22 (organic anion/cation transporter), member 15
22526	A_51_P478881	0.534	0.243	**0.456**	Ces4a	carboxylesterase 4A
55452	A_66_P136097	0.301	0.138	**0.457**	Trim13	tripartite motif-containing 13
28107	A_52_P70787	0.415	0.190	**0.457**	Brdt	bromodomain, testis-specific
17794	A_51_P193336	0.823	0.377	**0.458**	Nucb2	nucleobindin 2
22181	A_51_P459108	0.573	0.263	**0.458**	Insl6	insulin-like 6
53792	A_66_P102090	1.110	0.509	**0.458**	Pkmyt1	protein kinase, membrane associated tyrosine/threonine 1
25927	A_52_P420504	1.714	0.786	**0.459**	Acta2	actin, alpha 2, smooth muscle, aorta
1509	A_30_P01018933	0.251	0.115	**0.459**		
52672	A_55_P2359560	0.244	0.112	**0.460**	C230073G13Rik	RIKEN cDNA C230073G13 gene
30839	A_55_P1971889	1.712	0.788	**0.460**	F3	coagulation factor III
8780	A_30_P01026204	0.615	0.283	**0.460**		
25410	A_52_P354373	0.155	0.072	**0.461**	1190002F15Rik	RIKEN cDNA 1190002F15 gene
23799	A_52_P154741	1.320	0.610	**0.462**	Lgals6	lectin, galactose binding, soluble 6
33612	A_55_P1996941	0.255	0.118	**0.462**	Ube2c	ubiquitin-conjugating enzyme E2C
39342	A_55_P2051199	0.152	0.070	**0.462**	Gm13710	predicted gene 13710
9290	A_30_P01026714	0.200	0.093	**0.462**		
45087	A_55_P2113165	0.168	0.078	**0.463**	Camkk1	calcium/calmodulin-dependent protein kinase kinase 1, alpha
1535	A_30_P01018959	1.131	0.524	**0.463**		
54022	A_66_P106789	0.226	0.105	**0.463**		
39691	A_55_P2054743	0.572	0.265	**0.464**	Sh3bgrl2	SH3 domain binding glutamic acid-rich protein like 2
18011	A_51_P206445	0.173	0.080	**0.464**	Zfp773	zinc finger protein 773
15873	A_30_P01033297	0.180	0.083	**0.464**		
27097	A_52_P563375	0.171	0.079	**0.465**	Lgals2	lectin, galactose-binding, soluble 2
299	A_30_P01017723	0.625	0.291	**0.465**		
42222	A_55_P2081116	0.383	0.178	**0.465**	Fam89a	family with sequence similarity 89, member A
33307	A_55_P1993836	0.663	0.309	**0.466**	Dusp7	dual specificity phosphatase 7
25630	A_52_P381484	2.606	1.215	**0.466**	Spon2	spondin 2, extracellular matrix protein
704	A_30_P01018128	7.962	3.716	**0.467**		
16806	A_51_P133137	0.201	0.094	**0.468**	Kif20a	kinesin family member 20A
2566	A_30_P01019990	0.310	0.146	**0.470**		
26877	A_52_P537545	0.447	0.210	**0.470**	Smpd3	sphingomyelin phosphodiesterase 3, neutral
37481	A_55_P2032823	0.379	0.179	**0.471**	Trim2	tripartite motif-containing 2

326	A_30_P01017750	0.288	0.136	**0.472**		
51375	A_55_P2232325	0.179	0.085	**0.473**		
48192	A_55_P2151308	0.370	0.175	**0.473**		
20562	A_51_P360492	6.287	2.973	**0.473**	Mcm6	minichromosome maintenance deficient 6 (MIS5 homolog, S. pombe) (S. cerevisiae)
19791	A_51_P314186	0.743	0.352	**0.474**	Syne1	spectrin repeat containing, nuclear envelope 1
35525	A_55_P2014066	0.672	0.319	**0.474**		
2357	A_30_P01019781	0.366	0.174	**0.475**		
17348	A_51_P164014	0.318	0.151	**0.475**	Cenpe	centromere protein E
52562	A_55_P2346644	0.665	0.316	**0.475**	C730037M02Rik	RIKEN cDNA C730037M02 gene
33848	A_55_P1999022	0.598	0.284	**0.476**	Zfp760	zinc finger protein 760
35193	A_55_P2010936	1.430	0.683	**0.477**	Fbxo17	F-box protein 17
3657	A_30_P01021081	0.169	0.081	**0.479**		
3990	A_30_P01021414	0.133	0.064	**0.479**		
43898	A_55_P2099594	5.061	2.427	**0.480**	Scd3	stearoyl-coenzyme A desaturase 3
4205	A_30_P01021629	0.227	0.109	**0.480**		
31965	A_55_P1981719	0.136	0.065	**0.480**	Rreb1	ras responsive element binding protein 1
10682	A_30_P01028106	0.349	0.168	**0.480**		
24767	A_52_P27122	0.473	0.227	**0.481**	Ago4	argonaute RISC catalytic subunit 4
22269	A_51_P464308	0.225	0.108	**0.481**	Gnb4	guanine nucleotide binding protein (G protein), beta 4
32465	A_55_P1986341	41.985	20.192	**0.481**	Gm4956	predicted gene 4956
22264	A_51_P464146	0.529	0.255	**0.481**	Sowahb	sosondowah ankyrin repeat domain family member B
12601	A_30_P01030025	0.162	0.078	**0.482**		
26820	A_52_P530291	0.961	0.463	**0.482**	Pim1	proviral integration site 1
20887	A_51_P380178	12.989	6.280	**0.483**	Id3	inhibitor of DNA binding 3
5356	A_30_P01022780	0.319	0.154	**0.484**		
15379	A_30_P01032803	0.252	0.122	**0.485**		
10841	A_30_P01028265	0.315	0.153	**0.486**		
39707	A_55_P2054897	0.432	0.210	**0.486**	Rnd1	Rho family GTPase 1
19007	A_51_P265338	5.818	2.838	**0.488**	Nr0b2	nuclear receptor subfamily 0, group B, member 2
49250	A_55_P2165091	5.211	2.549	**0.489**	Acnat2	acyl-coenzyme A amino acid N-acyltransferase 2
4661	A_30_P01022085	0.305	0.149	**0.489**		
26790	A_52_P526852	0.892	0.437	**0.490**	Eri1	exoribonuclease 1
25937	A_52_P42194	0.334	0.164	**0.490**	Svil	supervillin
20936	A_51_P383194	3.666	1.799	**0.491**	Pde9a	phosphodiesterase 9A
22772	A_51_P491667	2.409	1.184	**0.491**	Derl3	Der1-like domain family, member 3
33066	A_55_P1991688	0.149	0.073	**0.492**	Rad51ap1	RAD51 associated protein 1

22019	A_51_P447785	2.647	1.302	**0.492**	Cyp2c55	cytochrome P450, family 2, subfamily c, polypeptide 55
51311	A_55_P2225246	0.148	0.073	**0.492**	4831440D22Rik	RIKEN cDNA 4831440D22 gene
20817	A_51_P376445	0.504	0.248	**0.493**	Rhox5	reproductive homeobox 5
2824	A_30_P01020248	0.360	0.178	**0.493**		
35644	A_55_P2015258	0.532	0.263	**0.495**	Trim13	tripartite motif-containing 13
36417	A_55_P2022604	0.338	0.167	**0.495**	Exoc3l4	exocyst complex component 3-like 4
37277	A_55_P2030752	7.433	3.685	**0.496**	Nedd4l	neural precursor cell expressed, developmentally down-regulated gene 4-like
37278	A_55_P2030756	0.413	0.205	**0.496**	Nedd4l	neural precursor cell expressed, developmentally down-regulated gene 4-like
51538	A_55_P2245904	0.153	0.076	**0.497**	9530003O04Rik	RIKEN cDNA 9530003O04 gene
20267	A_51_P342871	29.950	14.880	**0.497**	S100a10	S100 calcium binding protein A10 (calpactin)
46550	A_55_P2131190	1.938	0.964	**0.497**	Echdc3	enoyl Coenzyme A hydratase domain containing 3
18112	A_51_P211786	1.279	0.637	**0.498**	Chst13	carbohydrate (chondroitin 4) sulfotransferase 13
49652	A_55_P2170349	0.174	0.087	**0.499**	Klra22	killer cell lectin-like receptor subfamily A, member 22
53065	A_55_P2399688	0.122	0.061	**0.499**	Glipr2	GLI pathogenesis-related 2
22724	A_51_P488554	9.297	4.640	**0.499**	3010026O09Rik	RIKEN cDNA 3010026O09 gene
12742	A_30_P01030166	0.150	0.075	**0.500**		
18524	A_51_P235801	24.247	12.116	**0.500**	Zfp36l1	zinc finger protein 36, C3H type-like 1

Microarray data_Down478

Narabi_55681	ProbeName	LC(normalized)	LV(normalized)	Fold change	GeneSymbol	GeneName
44759	A_55_P2109564	2.166	0.018	**0.008**	AI504432	expressed sequence AI504432
52426	A_55_P2334157	1.064	0.012	**0.011**	A930038G18	uncharacterized protein A930038G18
39361	A_55_P2051377	0.999	0.012	**0.012**	Ccdc88c	coiled-coil domain containing 88C
12038	A_30_P01029462	1.120	0.014	**0.013**		
47660	A_55_P2144686	1.056	0.014	**0.013**	Dmbt1	deleted in malignant brain tumors 1
53419	A_55_P2459897	0.895	0.012	**0.014**	A2m	alpha-2-macroglobulin
5039	A_30_P01022463	1.490	0.021	**0.014**		
34470	A_55_P2004283	0.968	0.014	**0.014**		
41309	A_55_P2071354	0.916	0.013	**0.014**	Fam179a	family with sequence similarity 179, member A
9721	A_30_P01027145	0.938	0.015	**0.016**		
1608	A_30_P01019032	0.922	0.015	**0.016**		
14335	A_30_P01031759	0.835	0.014	**0.017**		
7599	A_30_P01025023	0.809	0.014	**0.017**		
52294	A_55_P2321004	3.259	0.059	**0.018**	Zfp882	zinc finger protein 882

30720	A_55_P1970876	0.846	0.016	**0.019**	Olfr389	olfactory receptor 389
43719	A_55_P2097543	0.719	0.014	**0.020**	Olfr1287	olfactory receptor 1287
14389	A_30_P01031813	0.757	0.015	**0.020**		
31401	A_55_P1976769	0.699	0.014	**0.020**	Olfr1093	olfactory receptor 1093
28530	A_55_P1952775	0.589	0.012	**0.020**	Olfr559	olfactory receptor 559
47482	A_55_P2142695	0.614	0.013	**0.021**	E330014E10Rik	RIKEN cDNA E330014E10 gene
35377	A_55_P2012624	0.652	0.013	**0.021**	Olfr1333	olfactory receptor 1333
29713	A_55_P1962334	0.573	0.012	**0.021**	Trim71	tripartite motif-containing 71
40153	A_55_P2059502	0.572	0.012	**0.021**	Zkscan16	zinc finger with KRAB and SCAN domains 16
10695	A_30_P01028119	0.568	0.012	**0.021**		
12386	A_30_P01029810	0.571	0.012	**0.022**		
20749	A_51_P372073	0.641	0.014	**0.022**	Slc6a18	solute carrier family 6 (neurotransmitter transporter), member 18
51800	A_55_P2272830	0.539	0.012	**0.023**		
39650	A_55_P2054372	0.643	0.015	**0.023**	Gm15299	predicted pseudogene 15299
2638	A_30_P01020062	0.512	0.012	**0.024**		
33246	A_55_P1993323	0.554	0.014	**0.025**	Olfr873	olfactory receptor 873
38233	A_55_P2040292	0.487	0.012	**0.025**	Gm16390	predicted gene 16390
44747	A_55_P2109445	0.625	0.016	**0.025**	Irf4	interferon regulatory factor 4
45990	A_55_P2124140	0.591	0.015	**0.025**		
18343	A_51_P225564	0.556	0.014	**0.025**	B3gnt4	UDP-GlcNAc:betaGal beta-1,3-N-acetylglucosaminyltransferase 4
402	A_30_P01017826	0.445	0.011	**0.026**		
36084	A_55_P2019367	0.470	0.012	**0.026**	Olfr1118	olfactory receptor 1118
6842	A_30_P01024266	0.465	0.012	**0.026**		
7676	A_30_P01025100	0.556	0.015	**0.026**		
9970	A_30_P01027394	0.541	0.014	**0.026**		
43864	A_55_P2099183	0.488	0.013	**0.026**	1700021F02Rik	RIKEN cDNA 1700021F02 gene
17546	A_51_P176583	0.530	0.014	**0.027**	1700109H08Rik	RIKEN cDNA 1700109H08 gene
45306	A_55_P2115832	0.554	0.015	**0.027**	Rbmy	RNA binding motif protein, Y chromosome
5359	A_30_P01022783	0.494	0.013	**0.027**		
7648	A_30_P01025072	0.518	0.014	**0.027**		
24791	A_52_P273865	0.548	0.015	**0.028**	Cdk14	cyclin-dependent kinase 14
2698	A_30_P01020122	0.487	0.014	**0.028**		
6236	A_30_P01023660	0.541	0.016	**0.029**		
40455	A_55_P2062667	0.981	0.028	**0.029**		
7813	A_30_P01025237	0.522	0.015	**0.029**		
11940	A_30_P01029364	0.492	0.015	**0.030**		

42795	A_55_P2087275	0.460	0.014	**0.030**	Vmn2r3	vomeronasal 2, receptor 3
23950	A_52_P174569	0.409	0.012	**0.030**	Hdhd1a	haloacid dehalogenase-like hydrolase domain containing 1A
36924	A_55_P2027416	0.510	0.015	**0.030**		
10936	A_30_P01028360	0.439	0.013	**0.030**		
28161	A_52_P74576	0.405	0.012	**0.031**	Ccdc65	coiled-coil domain containing 65
41751	A_55_P2075806	0.983	0.030	**0.031**	LOC102641274	disks large homolog 5-like
5794	A_30_P01023218	0.437	0.014	**0.031**		
50696	A_55_P2184641	0.391	0.012	**0.031**	Tunar	Tcl1 upstream neural differentiation associated RNA
5382	A_30_P01022806	0.458	0.014	**0.031**		
12551	A_30_P01029975	0.598	0.019	**0.031**		
7467	A_30_P01024891	0.468	0.015	**0.031**		
38515	A_55_P2043033	0.737	0.023	**0.032**	Pla2g3	phospholipase A2, group III
55482	A_66_P136749	0.467	0.015	**0.032**	Trim43c	tripartite motif-containing 43C
14838	A_30_P01032262	0.427	0.014	**0.032**		
28817	A_55_P1955084	0.389	0.013	**0.032**	Gm11757	predicted gene 11757
15452	A_30_P01032876	0.346	0.012	**0.033**		
51667	A_55_P2256770	0.357	0.012	**0.033**	4930555K05Rik	RIKEN cDNA 4930555K05 gene
12370	A_30_P01029794	0.438	0.015	**0.033**		
32943	A_55_P1990400	0.454	0.015	**0.033**	Htr4	5 hydroxytryptamine (serotonin) receptor 4
37365	A_55_P2031811	0.399	0.014	**0.034**		
49593	A_55_P2169544	0.370	0.013	**0.034**	LOC102640187	uncharacterized LOC102640187
40223	A_55_P2060323	0.352	0.012	**0.035**	3110062M04Rik	RIKEN cDNA 3110062M04 gene
47553	A_55_P2143406	0.441	0.015	**0.035**		
40728	A_55_P2065459	0.340	0.012	**0.035**	Olfr195	olfactory receptor 195
40944	A_55_P2067707	0.418	0.015	**0.035**	Mep1a	meprin 1 alpha
8623	A_30_P01026047	0.434	0.015	**0.035**		
23195	A_51_P515532	0.387	0.014	**0.035**	Tmem200a	transmembrane protein 200A
513	A_30_P01017937	0.400	0.014	**0.035**		
15227	A_30_P01032651	0.386	0.014	**0.035**		
5141	A_30_P01022565	0.636	0.022	**0.035**		
52121	A_55_P2301995	0.362	0.013	**0.035**	D930002L09Rik	RIKEN cDNA D930002L09 gene
32938	A_55_P1990369	0.429	0.015	**0.036**		
32444	A_55_P1986169	0.684	0.025	**0.036**		
13164	A_30_P01030588	0.381	0.014	**0.037**		
8392	A_30_P01025816	0.545	0.021	**0.039**		
15720	A_30_P01033144	0.319	0.012	**0.039**		

3751	A_30_P01021175	0.472	0.018	**0.039**		
19640	A_51_P304603	0.375	0.015	**0.039**	Gm10143	predicted gene 10143
46531	A_55_P2130997	0.350	0.014	**0.040**	D830050J10Rik	RIKEN cDNA D830050J10 gene
29308	A_55_P1959041	0.674	0.027	**0.040**	Fam115e	family with sequence similarity 115, member E
12809	A_30_P01030233	0.369	0.015	**0.041**		
14828	A_30_P01032252	0.689	0.028	**0.041**		
22551	A_51_P480136	0.294	0.012	**0.041**	Cryba2	crystallin, beta A2
36421	A_55_P2022629	1.155	0.048	**0.041**	Oxct2b	3-oxoacid CoA transferase 2B
35575	A_55_P2014565	0.328	0.014	**0.041**	Ces5a	carboxylesterase 5A
51243	A_55_P2217598	0.344	0.014	**0.042**	D10Ertd755e	DNA segment, Chr 10, ERATO Doi 755, expressed
8400	A_30_P01025824	0.472	0.020	**0.042**		
7509	A_30_P01024933	0.424	0.018	**0.042**		
46233	A_55_P2127254	0.294	0.012	**0.042**		
9076	A_30_P01026500	0.322	0.014	**0.044**		
8991	A_30_P01026415	0.287	0.013	**0.044**		
9624	A_30_P01027048	0.325	0.014	**0.044**		
25950	A_52_P42332	0.275	0.012	**0.044**	Mos	Moloney sarcoma oncogene
1374	A_30_P01018798	0.273	0.012	**0.044**		
3446	A_30_P01020870	0.282	0.013	**0.045**		
2491	A_30_P01019915	0.313	0.014	**0.045**		
5992	A_30_P01023416	0.342	0.015	**0.045**		
1767	A_30_P01019191	0.297	0.013	**0.045**		
6857	A_30_P01024281	0.361	0.016	**0.045**		
34120	A_55_P2001262	0.409	0.019	**0.045**		
5927	A_30_P01023351	0.277	0.013	**0.046**		
1083	A_30_P01018507	0.432	0.020	**0.046**		
50648	A_55_P2184023	0.243	0.011	**0.046**	Dnah7a	dynein, axonemal, heavy chain 7A
28311	A_52_P87804	0.321	0.015	**0.046**	Mfsd4	major facilitator superfamily domain containing 4
6224	A_30_P01023648	0.316	0.015	**0.047**		
47618	A_55_P2144155	0.272	0.013	**0.048**	Olfr518	olfactory receptor 518
48925	A_55_P2160737	0.269	0.013	**0.048**		
5637	A_30_P01023061	0.246	0.012	**0.049**		
50804	A_55_P2186051	0.355	0.018	**0.049**		
8648	A_30_P01026072	0.280	0.014	**0.049**		
33752	A_55_P1998169	0.611	0.030	**0.050**	Spef2	sperm flagellar 2
1422	A_30_P01018846	0.263	0.013	**0.050**		

13916	A_30_P01031340	0.256	0.013	**0.050**		
47032	A_55_P2137067	0.232	0.012	**0.052**		
29181	A_55_P1958018	0.254	0.013	**0.052**	LOC102637766	uncharacterized LOC102637766
15914	A_30_P01033338	0.228	0.012	**0.052**		
46441	A_55_P2129944	0.630	0.033	**0.053**		
47918	A_55_P2147941	0.287	0.015	**0.053**	Fam196b	family with sequence similarity 196, member B
13934	A_30_P01031358	0.426	0.023	**0.053**		
49946	A_55_P2174611	0.231	0.012	**0.054**		
18312	A_51_P223902	0.557	0.030	**0.055**		
12283	A_30_P01029707	0.208	0.012	**0.056**		
37936	A_55_P2037439	0.314	0.017	**0.056**	Depdc1a	DEP domain containing 1a
43944	A_55_P2100088	0.206	0.012	**0.056**	E130309D14Rik	RIKEN cDNA E130309D14 gene
52991	A_55_P2391534	0.521	0.029	**0.056**	4930459C07Rik	RIKEN cDNA 4930459C07 gene
35918	A_55_P2017799	0.251	0.014	**0.056**		
12014	A_30_P01029438	0.243	0.014	**0.057**		
13669	A_30_P01031093	0.212	0.012	**0.057**		
42667	A_55_P2085865	0.273	0.015	**0.057**		
27703	A_52_P639028	0.452	0.026	**0.058**	Myt1l	myelin transcription factor 1-like
40535	A_55_P2063486	0.424	0.025	**0.058**		
44811	A_55_P2110116	0.208	0.012	**0.058**		
225	A_30_P01017649	0.195	0.011	**0.058**		
47305	A_55_P2140487	0.223	0.013	**0.059**		
40532	A_55_P2063462	0.225	0.013	**0.059**		
24055	A_52_P187909	0.208	0.012	**0.060**	Slco1a6	solute carrier organic anion transporter family, member 1a6
9747	A_30_P01027171	0.248	0.015	**0.060**		
44823	A_55_P2110250	0.226	0.014	**0.060**	Rd3l	retinal degeneration 3-like
52223	A_55_P2313250	0.229	0.014	**0.060**	4921521D15Rik	RIKEN cDNA 4921521D15 gene
859	A_30_P01018283	0.438	0.026	**0.060**		
40122	A_55_P2059117	0.244	0.015	**0.061**	Skap1	src family associated phosphoprotein 1
36676	A_55_P2025203	0.200	0.012	**0.061**	Hrasls5	HRAS-like suppressor family, member 5
6354	A_30_P01023778	0.267	0.016	**0.061**		
14207	A_30_P01031631	0.626	0.039	**0.062**		
54764	A_66_P121512	0.207	0.013	**0.062**	Tekt3	tektin 3
1528	A_30_P01018952	0.228	0.014	**0.062**		
5281	A_30_P01022705	0.197	0.012	**0.063**		
5607	A_30_P01023031	0.240	0.015	**0.063**		

11332	A_30_P01028756	0.438	0.028	**0.064**		
1140	A_30_P01018564	0.220	0.014	**0.064**		
2194	A_30_P01019618	0.215	0.014	**0.064**		
14749	A_30_P01032173	0.222	0.014	**0.065**		
1751	A_30_P01019175	0.225	0.015	**0.065**		
13308	A_30_P01030732	0.216	0.014	**0.065**		
55675	A_66_P140874	0.216	0.014	**0.065**		
16647	A_51_P122855	0.214	0.014	**0.065**	Pax5	paired box 5
3474	A_30_P01020898	0.673	0.044	**0.066**		
51739	A_55_P2264483	0.207	0.014	**0.066**	Kif26b	kinesin family member 26B
40247	A_55_P2060547	0.228	0.015	**0.066**	Gm7897	predicted gene 7897
1167	A_30_P01018591	0.207	0.014	**0.066**		
53306	A_55_P2422383	0.187	0.012	**0.067**	E230024E03Rik	RIKEN cDNA E230024E03 gene
39793	A_55_P2055717	0.179	0.012	**0.067**	Vmn1r43	vomeronasal 1 receptor 43
35344	A_55_P2012281	0.208	0.014	**0.068**		
15182	A_30_P01032606	0.333	0.023	**0.068**		
38720	A_55_P2045007	0.174	0.012	**0.068**	Hrh1	histamine receptor H1
13585	A_30_P01031009	0.207	0.014	**0.068**		
51968	A_55_P2287105	0.190	0.013	**0.069**	4933407C09Rik	RIKEN cDNA 4933407C09 gene
47457	A_55_P2142376	0.492	0.034	**0.069**		
46277	A_55_P2127864	0.196	0.014	**0.070**	Spata17	spermatogenesis associated 17
29828	A_55_P1963259	0.171	0.012	**0.071**		
38249	A_55_P2040411	0.199	0.014	**0.071**		
2596	A_30_P01020020	0.181	0.013	**0.071**		
6687	A_30_P01024111	0.327	0.023	**0.072**		
42178	A_55_P2080598	0.294	0.021	**0.072**	5430416O09Rik	RIKEN cDNA 5430416O09 gene
46988	A_55_P2136606	0.677	0.049	**0.072**	Crnn	cornulin
37296	A_55_P2030996	0.164	0.012	**0.072**	9830107B12Rik	RIKEN cDNA 9830107B12 gene
7468	A_30_P01024892	0.442	0.032	**0.073**		
36518	A_55_P2023612	0.183	0.013	**0.073**	Ikzf1	IKAROS family zinc finger 1
51883	A_55_P2279225	0.182	0.013	**0.073**	4930516K23Rik	RIKEN cDNA 4930516K23 gene
31517	A_55_P1977870	0.179	0.013	**0.073**	Gpr112	G protein-coupled receptor 112
34884	A_55_P2007955	0.161	0.012	**0.074**	4933402N22Rik	RIKEN cDNA 4933402N22 gene
8650	A_30_P01026074	0.439	0.033	**0.074**		
29805	A_55_P1963046	0.176	0.013	**0.074**	Prl3c1	prolactin family 3, subfamily c, member 1
35512	A_55_P2013933	0.182	0.014	**0.075**		

20258	A_51_P342549	0.283	0.021	**0.075**	Hand1	heart and neural crest derivatives expressed transcript 1
5430	A_30_P01022854	0.161	0.012	**0.075**		
52404	A_55_P2331709	0.174	0.013	**0.075**	D630028G08Rik	RIKEN cDNA D630028G08 gene
26354	A_52_P476535	0.162	0.012	**0.075**	Defa23	defensin, alpha, 23
32656	A_55_P1987953	0.328	0.025	**0.076**	Gm5938	predicted gene 5938
46041	A_55_P2124761	0.157	0.012	**0.076**		
41966	A_55_P2078138	0.422	0.032	**0.076**	Otop1	otopetrin 1
5792	A_30_P01023216	0.198	0.015	**0.076**		
28015	A_52_P676819	0.173	0.013	**0.077**	Bnc1	basonuclin 1
14586	A_30_P01032010	0.176	0.014	**0.077**		
36955	A_55_P2027812	0.148	0.011	**0.077**	Arxes2	adipocyte-related X-chromosome expressed sequence 2
49150	A_55_P2163596	0.170	0.013	**0.077**		
51626	A_55_P2253279	0.162	0.013	**0.078**		
13804	A_30_P01031228	0.181	0.014	**0.078**		
48295	A_55_P2152672	0.394	0.031	**0.079**	LOC102637189	uncharacterized LOC102637189
17064	A_51_P148478	0.179	0.014	**0.080**	Olfr510	olfactory receptor 510
5276	A_30_P01022700	0.186	0.015	**0.080**		
11622	A_30_P01029046	0.154	0.012	**0.081**		
6215	A_30_P01023639	0.204	0.017	**0.082**		
11600	A_30_P01029024	0.311	0.026	**0.083**		
32427	A_55_P1985950	0.146	0.012	**0.083**	Xpnpep2	X-prolyl aminopeptidase (aminopeptidase P) 2, membrane-bound
48801	A_55_P2159155	0.146	0.012	**0.083**	Nsg2	neuron specific gene family member 2
35384	A_55_P2012694	0.249	0.021	**0.083**	Kcnh5	potassium voltage-gated channel, subfamily H (eag-related), member 5
24172	A_52_P201558	0.340	0.028	**0.084**	Rgs7bp	regulator of G-protein signalling 7 binding protein
9729	A_30_P01027153	0.140	0.012	**0.084**		
49927	A_55_P2174313	0.168	0.014	**0.084**	Olfr1045	olfactory receptor 1045
18216	A_51_P218041	0.215	0.018	**0.085**	Tatdn1	TatD DNase domain containing 1
7750	A_30_P01025174	0.143	0.012	**0.085**		
48441	A_55_P2154744	0.231	0.020	**0.085**		
11651	A_30_P01029075	0.130	0.011	**0.085**		
12730	A_30_P01030154	0.164	0.014	**0.086**		
26797	A_52_P527697	0.137	0.012	**0.086**	Olfr934	olfactory receptor 934
48931	A_55_P2160815	0.141	0.012	**0.086**	Foxd3	forkhead box D3
5275	A_30_P01022699	0.160	0.014	**0.087**		
40425	A_55_P2062464	0.148	0.013	**0.087**	Col12a1	collagen, type XII, alpha 1
50169	A_55_P2177473	0.162	0.014	**0.087**		

6624	A_30_P01024048	0.140	0.012	**0.088**		
19425	A_51_P290937	0.155	0.014	**0.088**	Cylc1	cylicin, basic protein of sperm head cytoskeleton 1
55246	A_66_P131662	0.173	0.015	**0.088**	Olfr1273-ps	olfactory receptor 1273, pseudogene
37184	A_55_P2029895	0.169	0.015	**0.088**		
3608	A_30_P01021032	0.174	0.015	**0.089**		
49357	A_55_P2166613	0.173	0.015	**0.089**		
11404	A_30_P01028828	0.134	0.012	**0.089**		
3366	A_30_P01020790	0.471	0.042	**0.089**		
29737	A_55_P1962503	0.332	0.030	**0.089**	Nup62cl	nucleoporin 62 C-terminal like
37624	A_55_P2034195	0.140	0.013	**0.090**	Sprr3	small proline-rich protein 3
9717	A_30_P01027141	0.149	0.013	**0.090**		
20606	A_51_P363642	0.492	0.044	**0.090**	1700088E04Rik	RIKEN cDNA 1700088E04 gene
49995	A_55_P2175145	0.151	0.014	**0.090**	LOC102638466	uncharacterized LOC102638466
12443	A_30_P01029867	0.134	0.012	**0.091**		
14736	A_30_P01032160	0.633	0.058	**0.091**		
10874	A_30_P01028298	0.145	0.013	**0.091**		
54531	A_66_P116873	0.686	0.063	**0.092**	Gm1070	predicted gene 1070
6205	A_30_P01023629	0.379	0.035	**0.092**		
15971	A_30_P01033395	0.153	0.014	**0.093**		
53135	A_55_P2406802	0.138	0.013	**0.093**	4930511J24Rik	RIKEN cDNA 4930511J24 gene
52077	A_55_P2296320	0.123	0.011	**0.093**	Gm13112	predicted gene 13112
3482	A_30_P01020906	0.165	0.015	**0.094**		
19582	A_51_P301336	0.142	0.013	**0.094**	Olfr1453	olfactory receptor 1453
21290	A_51_P403564	0.147	0.014	**0.094**	Lhx5	LIM homeobox protein 5
37830	A_55_P2036290	0.472	0.044	**0.094**	Gm10432	predicted gene 10432
12169	A_30_P01029593	0.140	0.013	**0.094**		
292	A_30_P01017716	0.119	0.011	**0.094**		
22788	A_51_P492456	0.308	0.029	**0.095**	Has1	hyaluronan synthase1
15316	A_30_P01032740	0.139	0.013	**0.096**		
31616	A_55_P1978735	0.310	0.030	**0.096**	Slx4ip	SLX4 interacting protein
38404	A_55_P2041988	0.119	0.011	**0.096**		
11646	A_30_P01029070	0.394	0.038	**0.096**		
21990	A_51_P445985	0.121	0.012	**0.096**	Slc12a3	solute carrier family 12, member 3
2616	A_30_P01020040	0.298	0.029	**0.096**		
2240	A_30_P01019664	0.139	0.014	**0.098**		
26669	A_52_P512519	0.139	0.014	**0.098**	Cdc7	cell division cycle 7 (S. cerevisiae)

31003	A_55_P1973264	0.150	0.015	**0.098**	Spint5	serine protease inhibitor, Kunitz type 5
27035	A_52_P5567	0.190	0.019	**0.099**	Fam50b	family with sequence similarity 50, member B
54959	A_66_P125873	0.447	0.045	**0.101**	Nup93	nucleoporin 93
51958	A_55_P2285817	0.173	0.018	**0.101**	4932441J04Rik	RIKEN cDNA 4932441J04 gene
14014	A_30_P01031438	0.270	0.027	**0.102**		
8563	A_30_P01025987	0.251	0.026	**0.102**		
7201	A_30_P01024625	0.128	0.013	**0.102**		
41877	A_55_P2077181	0.123	0.013	**0.103**		
37359	A_55_P2031736	0.142	0.015	**0.103**	Npas3	neuronal PAS domain protein 3
30826	A_55_P1971774	0.133	0.014	**0.103**	Fsip1	fibrous sheath-interacting protein 1
5942	A_30_P01023366	0.116	0.012	**0.103**		
9327	A_30_P01026751	0.245	0.025	**0.104**		
33029	A_55_P1991278	0.146	0.015	**0.104**	Gm10826	predicted gene 10826
37841	A_55_P2036392	0.148	0.015	**0.104**	Slc39a12	solute carrier family 39 (zinc transporter), member 12
44974	A_55_P2112070	0.134	0.014	**0.105**		
52326	A_55_P2324182	0.400	0.043	**0.107**	C730002L08Rik	RIKEN cDNA C730002L08 gene
7978	A_30_P01025402	0.116	0.012	**0.107**		
43967	A_55_P2100331	0.444	0.048	**0.107**	Tcp11l1	t-complex 11 like 1
3695	A_30_P01021119	0.136	0.015	**0.107**		
25883	A_52_P413193	0.116	0.012	**0.107**	Gpr173	G-protein coupled receptor 173
42828	A_55_P2087607	0.465	0.050	**0.108**	Cp	ceruloplasmin
14266	A_30_P01031690	0.280	0.030	**0.108**		
48165	A_55_P2150972	0.111	0.012	**0.108**		
39921	A_55_P2057118	0.104	0.011	**0.109**	Mid1	midline 1
13820	A_30_P01031244	0.364	0.040	**0.109**		
11981	A_30_P01029405	0.108	0.012	**0.109**		
23704	A_52_P143037	0.112	0.012	**0.109**	Gabra5	gamma-aminobutyric acid (GABA) A receptor, subunit alpha 5
2680	A_30_P01020104	0.124	0.014	**0.110**		
27550	A_52_P619215	0.298	0.033	**0.110**	Ubqlnl	ubiquilin-like
6338	A_30_P01023762	0.135	0.015	**0.110**		
9427	A_30_P01026851	0.228	0.025	**0.110**		
42726	A_55_P2086522	0.110	0.012	**0.110**	Ssxb7	synovial sarcoma, X member B, breakpoint 7
39397	A_55_P2051787	0.123	0.014	**0.110**	Gbx1	gastrulation brain homeobox 1
29698	A_55_P1962224	0.280	0.031	**0.111**	Afap1l2	actin filament associated protein 1-like 2
44511	A_55_P2106700	0.108	0.012	**0.111**	Vmn2r59	vomeronasal 2, receptor 59
48051	A_55_P2149714	0.121	0.014	**0.112**	Olfr1258	olfactory receptor 1258

12491	A_30_P01029915	0.132	0.015	**0.112**		
4380	A_30_P01021804	0.116	0.013	**0.113**		
50576	A_55_P2183035	0.111	0.013	**0.113**	Exoc7	exocyst complex component 7
52978	A_55_P2390604	0.177	0.020	**0.114**	4930448A20Rik	RIKEN cDNA 4930448A20 gene
32237	A_55_P1984343	0.110	0.012	**0.114**	Rhox2h	reproductive homeobox 2H
6844	A_30_P01024268	0.107	0.012	**0.115**		
13035	A_30_P01030459	0.097	0.011	**0.116**		
12428	A_30_P01029852	0.105	0.012	**0.116**		
18423	A_51_P230098	0.105	0.012	**0.117**	Pbk	PDZ binding kinase
11709	A_30_P01029133	0.317	0.037	**0.117**		
7166	A_30_P01024590	0.100	0.012	**0.117**		
44201	A_55_P2102988	0.104	0.012	**0.118**		
40496	A_55_P2063082	0.114	0.014	**0.118**		
55070	A_66_P128342	0.158	0.019	**0.118**		
12933	A_30_P01030357	0.125	0.015	**0.119**		
9121	A_30_P01026545	0.108	0.013	**0.119**		
14134	A_30_P01031558	0.124	0.015	**0.119**		
45936	A_55_P2123486	0.157	0.019	**0.120**	Map3k10	mitogen-activated protein kinase kinase kinase 10
46589	A_55_P2131642	0.148	0.018	**0.120**	Trim50	tripartite motif-containing 50
8192	A_30_P01025616	0.097	0.012	**0.120**		
25775	A_52_P401484	0.098	0.012	**0.120**	Inha	inhibin alpha
18453	A_51_P232281	0.325	0.039	**0.121**	Pla2g2d	phospholipase A2, group IID
10818	A_30_P01028242	0.107	0.013	**0.121**		
41959	A_55_P2078084	0.362	0.044	**0.122**	Gm13011	predicted gene 13011
48840	A_55_P2159705	0.109	0.013	**0.122**	Acvr1	activin A receptor, type 1
53747	A_66_P101393	0.111	0.014	**0.122**	A530001N23Rik	RIKEN cDNA A530001N23 gene
42542	A_55_P2084547	0.355	0.044	**0.123**	9130019O22Rik	RIKEN cDNA 9130019O22 gene
19648	A_51_P305230	0.528	0.065	**0.124**	Elavl3	ELAV (embryonic lethal, abnormal vision, Drosophila)-like 3 (Hu antigen C)
55556	A_66_P138298	0.098	0.012	**0.124**	Gm4371	eukaryotic translation initiation factor 3, subunit I pseudogene
43182	A_55_P2091496	0.119	0.015	**0.124**	Dppa3	developmental pluripotency-associated 3
35829	A_55_P2017060	0.111	0.014	**0.125**	Pou1f1	POU domain, class 1, transcription factor 1
17919	A_51_P201490	0.203	0.025	**0.125**	Olfr242	olfactory receptor 242
26840	A_52_P533129	0.099	0.012	**0.125**	Mixl1	Mix1 homeobox-like 1 (Xenopus laevis)
44184	A_55_P2102833	0.461	0.058	**0.126**	LOC102634746	uncharacterized LOC102634746
37949	A_55_P2037568	0.121	0.015	**0.126**		
45923	A_55_P2123283	0.106	0.014	**0.127**	Kcna6	potassium voltage-gated channel, shaker-related, subfamily, member 6

26712	A_52_P517224	0.095	0.012	**0.128**	Trim63	tripartite motif-containing 63
36871	A_55_P2026950	0.119	0.015	**0.128**	C530030P08Rik	RIKEN cDNA C530030P08 gene
44274	A_55_P2103927	0.093	0.012	**0.128**		
7088	A_30_P01024512	0.091	0.012	**0.128**		
5713	A_30_P01023137	0.163	0.021	**0.128**		
28924	A_55_P1955861	0.185	0.024	**0.128**	Cux2	cut-like homeobox 2
5120	A_30_P01022544	0.126	0.016	**0.129**		
24151	A_52_P199557	0.097	0.012	**0.129**	1700034J05Rik	RIKEN cDNA 1700034J05 gene
1913	A_30_P01019337	0.108	0.014	**0.129**		
15669	A_30_P01033093	0.142	0.018	**0.130**		
44063	A_55_P2101506	0.329	0.043	**0.130**	Fgf8	fibroblast growth factor 8
343	A_30_P01017767	0.113	0.015	**0.130**		
47031	A_55_P2137049	0.107	0.014	**0.131**	AA467197	expressed sequence AA467197
8691	A_30_P01026115	0.089	0.012	**0.131**		
36905	A_55_P2027235	0.135	0.018	**0.131**	BC048679	cDNA sequence BC048679
15550	A_30_P01032974	0.087	0.011	**0.132**		
42887	A_55_P2088268	0.109	0.014	**0.133**	Olfr782	olfactory receptor 782
12106	A_30_P01029530	0.540	0.072	**0.133**		
35785	A_55_P2016647	0.444	0.060	**0.134**	Cdk1	cyclin-dependent kinase 1
40824	A_55_P2066448	0.105	0.014	**0.134**	Frmd7	FERM domain containing 7
14305	A_30_P01031729	0.130	0.018	**0.135**		
14048	A_30_P01031472	0.112	0.015	**0.135**		
44032	A_55_P2101137	0.426	0.058	**0.135**	Olfr1123	olfactory receptor 1123
28360	A_52_P91043	0.145	0.020	**0.136**	Tprg	transformation related protein 63 regulated
52936	A_55_P2386095	0.091	0.012	**0.137**	9630039A02Rik	RIKEN cDNA 9630039A02 gene
28282	A_52_P85334	0.085	0.012	**0.137**	Olfr146	olfactory receptor 146
51756	A_55_P2267240	0.097	0.013	**0.137**	4930471G03Rik	RIKEN cDNA 4930471G03 gene
28364	A_52_P91454	0.144	0.020	**0.137**	1700106J16Rik	RIKEN cDNA 1700106J16 gene
13173	A_30_P01030597	0.428	0.059	**0.138**		
16175	A_30_P01033599	0.182	0.025	**0.138**		
42940	A_55_P2088785	0.171	0.024	**0.139**	Tceal7	transcription elongation factor A (SII)-like 7
51058	A_55_P2201395	0.089	0.012	**0.139**	2410017I17Rik	RIKEN cDNA 2410017I17 gene
54762	A_66_P121495	0.257	0.036	**0.139**	Psat1	phosphoserine aminotransferase 1
51070	A_55_P2202184	0.087	0.012	**0.140**	4933400F21Rik	RIKEN cDNA 4933400F21 gene
31301	A_55_P1975752	0.088	0.012	**0.140**		
28429	A_52_P98614	0.093	0.013	**0.140**	Slc6a2	solute carrier family 6 (neurotransmitter transporter, noradrenalin), member 2

34507	A_55_P2004624	0.078	0.011	**0.141**		
34167	A_55_P2001764	0.079	0.011	**0.141**		
51895	A_55_P2279852	0.148	0.021	**0.141**	AU019202	expressed sequence AU019202
50407	A_55_P2180824	0.225	0.032	**0.141**		
45084	A_55_P2113141	0.140	0.020	**0.141**	Star	steroidogenic acute regulatory protein
10308	A_30_P01027732	0.078	0.011	**0.142**		
53142	A_55_P2407367	0.378	0.054	**0.143**	9130001E16Rik	RIKEN cDNA 9130001E16 gene
45698	A_55_P2120689	0.205	0.029	**0.143**		
50611	A_55_P2183572	0.224	0.032	**0.143**		
40060	A_55_P2058557	0.287	0.041	**0.143**	Dnajb5	DnaJ (Hsp40) homolog, subfamily B, member 5
50877	A_55_P2187030	0.096	0.014	**0.143**		
49159	A_55_P2163729	0.150	0.022	**0.144**	Tvp23a	trans-golgi network vesicle protein 23A
20011	A_51_P327934	0.080	0.011	**0.144**	Rfx2	regulatory factor X, 2 (influences HLA class II expression)
27238	A_52_P580634	0.269	0.039	**0.144**	Rbm46	RNA binding motif protein 46
35413	A_55_P2012979	0.090	0.013	**0.146**	Itgb2l	integrin beta 2-like
22032	A_51_P448478	0.098	0.014	**0.146**	Slc10a4	solute carrier family 10 (sodium/bile acid cotransporter family), member 4
33273	A_55_P1993517	0.246	0.036	**0.146**	Tbc1d14	TBC1 domain family, member 14
39309	A_55_P2050843	0.084	0.012	**0.147**	Olfr969	olfactory receptor 969
26716	A_52_P517762	0.099	0.015	**0.148**	Slc44a4	solute carrier family 44, member 4
23433	A_52_P111845	0.084	0.012	**0.148**	Nxph2	neurexophilin 2
5029	A_30_P01022453	0.100	0.015	**0.148**		
6431	A_30_P01023855	0.087	0.013	**0.149**		
43540	A_55_P2095663	0.085	0.013	**0.150**	Pgr	progesterone receptor
26810	A_52_P52910	0.077	0.012	**0.150**	Fam151a	family with sequence simliarity 151, member A
3360	A_30_P01020784	0.217	0.033	**0.150**		
2611	A_30_P01020035	0.123	0.019	**0.150**		
230	A_30_P01017654	0.082	0.012	**0.150**		
33427	A_55_P1994877	0.090	0.014	**0.151**	Olfr1022	olfactory receptor 1022
598	A_30_P01018022	0.102	0.015	**0.151**		
14096	A_30_P01031520	0.102	0.015	**0.151**		
12536	A_30_P01029960	0.081	0.012	**0.152**		
13591	A_30_P01031015	0.101	0.015	**0.152**		
37782	A_55_P2035817	0.095	0.014	**0.152**	Gm1043	predicted gene 1043
29487	A_55_P1960441	0.092	0.014	**0.152**		
10456	A_30_P01027880	0.120	0.018	**0.153**		
39535	A_55_P2053168	0.079	0.012	**0.153**		

53210	A_55_P2414159	0.077	0.012	**0.153**	Vwa2	von Willebrand factor A domain containing 2
39127	A_55_P2048808	0.101	0.016	**0.153**	4930588K23Rik	RIKEN cDNA 4930588K23 gene
6924	A_30_P01024348	0.089	0.014	**0.154**		
4235	A_30_P01021659	0.080	0.012	**0.154**		
6930	A_30_P01024354	0.083	0.013	**0.154**		
13330	A_30_P01030754	0.206	0.032	**0.156**		
42028	A_55_P2078810	0.113	0.018	**0.156**	Olfr95	olfactory receptor 95
14506	A_30_P01031930	0.282	0.044	**0.157**		
30297	A_55_P1967310	0.075	0.012	**0.157**	Gm10437	predicted gene 10437
45727	A_55_P2121023	0.426	0.067	**0.157**	4930413G21Rik	RIKEN cDNA 4930413G21 gene
52533	A_55_P2344593	0.139	0.022	**0.157**	4930554G24Rik	RIKEN cDNA 4930554G24 gene
20776	A_51_P373901	0.080	0.013	**0.157**	Rsph1	radial spoke head 1 homolog (Chlamydomonas)
28123	A_52_P71756	0.074	0.012	**0.158**	Prb1	proline-rich protein BstNI subfamily 1
21504	A_51_P416858	0.090	0.014	**0.158**	Myl1	myosin, light polypeptide 1
35446	A_55_P2013316	0.196	0.031	**0.158**	Ankrd53	ankyrin repeat domain 53
32175	A_55_P1983769	0.079	0.013	**0.158**	Birc5	baculoviral IAP repeat-containing 5
28283	A_52_P85765	0.077	0.012	**0.159**	Stard6	StAR-related lipid transfer (START) domain containing 6
11641	A_30_P01029065	0.196	0.031	**0.159**		
7870	A_30_P01025294	0.093	0.015	**0.159**		
2617	A_30_P01020041	0.092	0.015	**0.160**		
6017	A_30_P01023441	0.076	0.012	**0.160**		
14231	A_30_P01031655	0.082	0.013	**0.160**		
52733	A_55_P2364841	0.081	0.013	**0.161**	4930461C15Rik	RIKEN cDNA 4930461C15 gene
46487	A_55_P2130497	0.093	0.015	**0.161**		
7910	A_30_P01025334	0.085	0.014	**0.162**		
48974	A_55_P2161353	0.069	0.011	**0.163**	Kcnmb4	potassium large conductance calcium-activated channel, subfamily M, beta member 4
32520	A_55_P1986818	0.069	0.011	**0.164**		
34799	A_55_P2007210	0.091	0.015	**0.164**	Abca15	ATP-binding cassette, sub-family A (ABC1), member 15
4524	A_30_P01021948	0.081	0.013	**0.164**		
42944	A_55_P2088860	0.077	0.013	**0.165**		
23842	A_52_P160397	0.094	0.016	**0.166**	2410137M14Rik	RIKEN cDNA 2410137M14 gene
47159	A_55_P2138703	0.121	0.020	**0.166**		
1489	A_30_P01018913	0.092	0.015	**0.166**		
28400	A_52_P95294	0.073	0.012	**0.166**	Tcerg1l	transcription elongation regulator 1-like
50464	A_55_P2181404	0.090	0.015	**0.167**	Dnah9	dynein, axonemal, heavy chain 9

15682	A_30_P01033106	0.112	0.019	**0.168**		
25051	A_52_P307904	0.070	0.012	**0.168**	Gpr128	G protein-coupled receptor 128
49695	A_55_P2170817	0.108	0.018	**0.169**	AI847159	expressed sequence AI847159
51044	A_55_P2200523	0.070	0.012	**0.169**	8030456M14Rik	RIKEN cDNA 8030456M14 gene
14893	A_30_P01032317	0.066	0.011	**0.170**		
914	A_30_P01018338	0.255	0.044	**0.172**		
11303	A_30_P01028727	0.085	0.015	**0.172**		
53958	A_66_P105771	0.115	0.020	**0.175**	Gm10548	ribosomal protein L29 pseudogene
36034	A_55_P2018863	0.085	0.015	**0.176**	Defa-rs7	defensin, alpha, related sequence 7
39925	A_55_P2057168	0.182	0.032	**0.177**		
44854	A_55_P2110579	0.136	0.024	**0.178**		
49258	A_55_P2165210	0.231	0.041	**0.178**	Lrrtm4	leucine rich repeat transmembrane neuronal 4
23163	A_51_P513530	0.087	0.016	**0.180**	Spag5	sperm associated antigen 5
16979	A_51_P143296	0.088	0.016	**0.181**	Myh8	myosin, heavy polypeptide 8, skeletal muscle, perinatal
32413	A_55_P1985825	0.117	0.021	**0.181**		
3586	A_30_P01021010	0.131	0.024	**0.182**		
13206	A_30_P01030630	0.092	0.017	**0.182**		
53231	A_55_P2415576	0.298	0.055	**0.184**	4930592A05Rik	RIKEN cDNA 4930592A05 gene
10777	A_30_P01028201	0.119	0.022	**0.186**		
33732	A_55_P1997997	0.103	0.019	**0.187**	Klra23	killer cell lectin-like receptor subfamily A, member 23
54102	A_66_P108468	0.228	0.043	**0.188**	2610528A11Rik	RIKEN cDNA 2610528A11 gene
30081	A_55_P1965462	0.132	0.025	**0.189**		
44721	A_55_P2109152	0.237	0.045	**0.190**		
26896	A_52_P539250	0.077	0.015	**0.191**	Adam30	a disintegrin and metallopeptidase domain 30
55388	A_66_P134808	0.151	0.029	**0.191**	Nos1ap	nitric oxide synthase 1 (neuronal) adaptor protein
15255	A_30_P01032679	0.119	0.023	**0.192**		
1993	A_30_P01019417	0.123	0.024	**0.192**		
51838	A_55_P2275590	0.091	0.018	**0.192**	A630012P03Rik	RIKEN cDNA A630012P03 gene
35405	A_55_P2012894	0.334	0.064	**0.193**	Sbspon	somatomedin B and thrombospondin, type 1 domain containing
24565	A_52_P246703	0.164	0.032	**0.194**	Ak7	adenylate kinase 7
24416	A_52_P229943	0.280	0.055	**0.195**	Slc51b	solute carrier family 51, beta subunit
43563	A_55_P2095899	0.269	0.053	**0.197**	2310057B04Rik	RIKEN cDNA 2310057B04 gene
2368	A_30_P01019792	0.225	0.045	**0.200**		
