# Supplementary material for: Vildagliptin and its metabolite M20.7 induce the expression of S100A8 and S100A9 in human hepatoma HepG2 and leukemia HL-60 cells
Source: Sci Rep. 2016 Oct 19;6:35633. doi: 10.1038/srep35633 (PMC5069476; doi:10.1038/srep35633)
Supplement: Supplementary Information [file srep35633-s2.pdf]

Vildagliptin and its metabolite M20.7 induce the expression of S100A8 and S100A9 in human hepatoma HepG2 and leukemia HL-60 cells

Mitsutoshi Asakura, Fumika Karaki, Hideaki Fujii, Koichiro Atsuda, Tomoo Itoh & Ryoichi Fujiwara

Supplementary materials

Supplemental Table S1. Top 10 probes that were down-regulated more than 2-fold and 5-fold among the 292 and 478 genes.

	GeneSymbol	Gene name	Fold change (Vildagliptin/Control)
Down-regulated more than 2-fold*	Olfr394	Olfactory receptor 394	**0.098**
	Smcp	Sperm mitochondria-associated cysteine-rich protein	**0.101**
	Saa1	Serum amyloid A 1	**0.121**
	Lcn2	Lipocalin 2	**0.121**
	Saa2	Serum amyloid A 2	**0.123**
	Ric3	Resistance to inhibitors of cholinesterase 3 homolog (C. elegans)	**0.125**
	Arntl	Aryl hydrocarbon receptor nuclear translocator-like	**0.139**
	Ccdc142	Coiled-coil domain containing 142	**0.152**
	Cldnd2	Claudin domain containing 2	**0.154**
	Cnn1	Calponin 1	**0.158**
Down-regulated more than 5-fold**	Ccdc88c	Coiled-coil domain containing 88C	**0.012**
	Dmbt1	Deleted in malignant brain tumors 1	**0.013**
	A2m	Alpha-2-macroglobulin	**0.014**
	Fam179a	Family with sequence similarity 179, member A	**0.014**
	Zfp882	Zinc finger protein 882	**0.018**
	Olfr389	Olfactory receptor 389	**0.019**
	Olfr1287	Olfactory receptor 1287	**0.020**
	Olfr1093	Olfactory receptor 1093	**0.020**
	Olfr559	Olfactory receptor 559	**0.020**
	Olfr1333	Olfactory receptor 1333	**0.021**

*Among probes that were detected in both the samples (the livers of control and vildagliptin-treated mice), the probes decreased more than 2-fold were shown.

**Among probes that were detected in the liver of control mice and not detected in the liver of vildagliptin-treated mice, the probes decreased more than 5-fold were shown.

Supplemental Table S2. Primer sequences used for real-time reverse transcription-polymerase chain reaction analyses.

	Target	Forward primers (5' to 3')	Reverse primers (5' to 3')
Human	CABYR	CTGCGGGGCTATAAAAACGC	TGAGGCCATAGGGTACGACA
	PIP5K1A	CGTTGGGAAGATTCGATTCCG	CAAGGTACAGGAAGGGACCG
	CIART	TCATCATTGGGTGGTGGCAA	GTGGTGTGGATCCAGGTGAG
	MT1	CGTGCGCCTTATAGCCTCTC	TCTTCTTGCAGGAGGTGCAT
	FBF1	CAACTGCACGAGAAAGACCG	TTTCTGGGTGCTCGGGAAAC
	LPIN1	GATGTCAATGCACCCTGAGA	GTGTTTGCAATACAAAGGCG
	PFKFB3	AAGAAGGGACCTAACCCGCT	CCGGGAGCCTTTCATGTTTT
	MBP	AGGACTGTCCCTGAGCAGAT	TGGGTGATCCAGAGCGACTA
	USP2	AACGAGGTGAACCGAGTGAC	GTACACGTCAGCGAGCTCTT
	S100A9	GCAGCTGGAACGCAACATAG	TGTGTCCAGGTCCTCCATGA
	S100A8	GCCGTCTACAGGGATGACCT	CCACGCCCATCTTTATCACCA
	PLIN4	GCTGCAACCTTCGGAAAAGC	TAAGTGCAGACCGAGTGGTG
	MARS2	GGGCGTCTATGAAGGTTGGT	CGGAACTGGGAAAGCCTGAA
	MT2	ATGCACCTCCTGCAAGAAAAG	CGGTCACGGTCAGGGTTGTA
	SMR3A	GGAGATTTCCACCACCCCTTT	TAGGGAGGGCAGGATCAGTT
	GALNTL5	GGTGTGCCCCCTGATAGATG	ACATTGCAGGTGACCGGATT
	ESRRB	AGTGCCCATGCACAAACTCT	GGGAGTTGCTCTGATCCCTG
	GAPDH*	CCAGGGCTGCTTTTAACTC	GCTCCCCCCTGCAAATGA
Mouse	Mt1	GAATGGACCCCAACTGCTCC	GTTCGTCACATCAGGCACAG
	Mt2	GTGCTGGCCATATCCCTTGA	GCGGTTGAAGATCGACGAGA
	S100a8	AGGAAATCACCATGCCCTCTAC	TGAGATGCCACACCCACTTTT
	S100a9	GACTCTTTAGCCTTGAAGAGCAAG	AAAGGTTGCCAACTGTGCTTC
	CPH**	CAGACGCCACTGTCGCTTT	TGTCTTTGGAACTTTGTCTGCAA

* Nakamura, A., Nakajima, M., Yamanaka, H., Fujiwara, R. & Yokoi, T. Expression of UGT1A and UGT2B mRNA in human normal tissues and various cell lines. *Drug Metab. Dispos.* **36**, 1461–1464 (2008).

** Fujiwara, R., Chen, S., Karin, M. & Tukey, R. H. Reduced expression of UGT1A1 in intestines of humanized UGT1 mice via inactivation of NF-κB leads to hyperbilirubinemia. *Gastroenterology* **142**, 109–118 (2012).

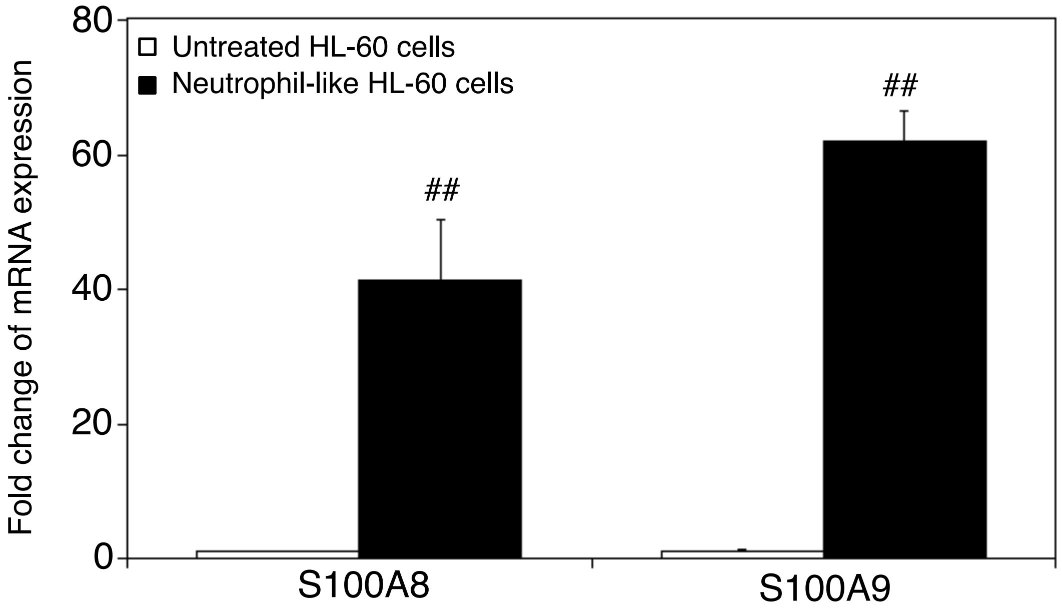

Supplementary Figure S1. The mRNA expression levels of S100A8 and S100A9 in untreated and neutrophil-like HL-60 cells. To confirm HL-60 cells were differentiated to neutrophil-like HL-60 cells, the mRNA expression levels of S100A8 and S100A9 in untreated and neutrophil-like HL-60 cells were measured by real-time RT-PCR analysis. Expression was normalized with the expression of glyceraldehyde-3-phosphate dehydrogenase (GAPDH). Data represent the means ± S.D. of three independent experiments. $^{\#\#}$, $P < 0.01$, compared with the untreated HL-60 cells.

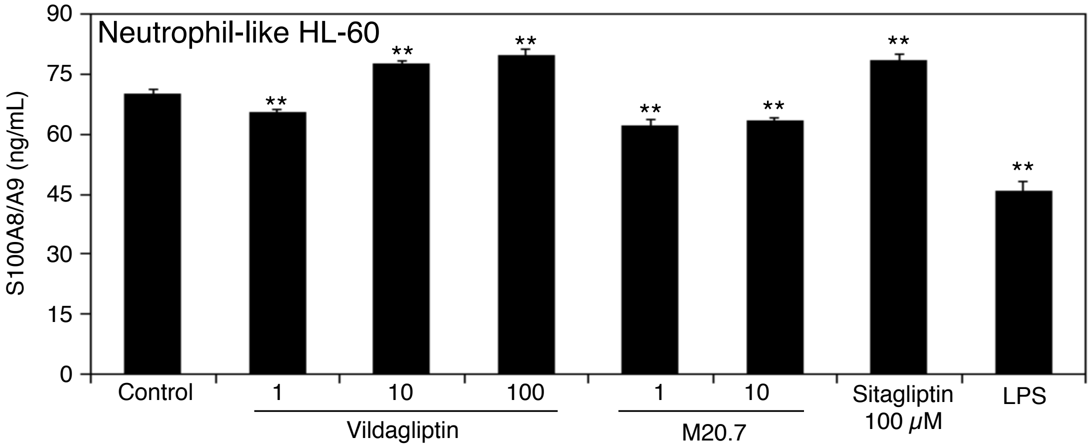

Supplementary Figure S2. Effects of vildagliptin, M20.7, and sitagliptin on the release of S100A8/A9 complex from neutrophil-like HL-60 cells. HL-60 cells were differentiated to neutrophil-like HL-60 cells following treatment with 1.3% DMSO for 3 days and then the neutrophil-like HL-60 cells were treated with the indicated concentration (μM) of vildagliptin, M20.7, sitagliptin, or LPS (10 μg/mL) for 24 h. The final concentration of methanol in the culture medium was 0.1%. The release of S100A8/A9 complex in the culturing medium was measured by ELISA. Data represent the means ± S.D. of three independent experiments. **, $P < 0.01$, compared with control (0.1% methanol).
